# Commercial Essential Oils as Potential Antimicrobials to Treat Skin Diseases

**DOI:** 10.1155/2017/4517971

**Published:** 2017-05-04

**Authors:** Ané Orchard, Sandy van Vuuren

**Affiliations:** Department of Pharmacy and Pharmacology, Faculty of Health Sciences, University of the Witwatersrand, 7 York Road, Parktown 2193, South Africa

## Abstract

Essential oils are one of the most notorious natural products used for medical purposes. Combined with their popular use in dermatology, their availability, and the development of antimicrobial resistance, commercial essential oils are often an option for therapy. At least 90 essential oils can be identified as being recommended for dermatological use, with at least 1500 combinations. This review explores the fundamental knowledge available on the antimicrobial properties against pathogens responsible for dermatological infections and compares the scientific evidence to what is recommended for use in common layman's literature. Also included is a review of combinations with other essential oils and antimicrobials. The minimum inhibitory concentration dilution method is the preferred means of determining antimicrobial activity. While dermatological skin pathogens such as* Staphylococcus aureus* have been well studied, other pathogens such as* Streptococcus pyogenes*,* Propionibacterium acnes*,* Haemophilus influenzae*, and* Brevibacterium *species have been sorely neglected. Combination studies incorporating oil blends, as well as interactions with conventional antimicrobials, have shown that mostly synergy is reported. Very few viral studies of relevance to the skin have been made. Encouragement is made for further research into essential oil combinations with other essential oils, antimicrobials, and carrier oils.

## 1. Introduction

The skin is the body's largest mechanical barrier against the external environment and invasion by microorganisms. It is responsible for numerous functions such as heat regulation and protecting the underlying organs and tissue [1, 2]. The uppermost epidermal layer is covered by a protective keratinous surface which allows for the removal of microorganisms via sloughing off of keratinocytes and acidic sebaceous secretions. This produces a hostile environment for microorganisms. In addition to these defences, the skin also consists of natural microflora which offers additional protection by competitively inhibiting pathogenic bacterial growth by competing for nutrients and attachment sites and by producing metabolic products that inhibit microbial growth. The skin's natural microflora includes species of* Corynebacterium*, staphylococci, streptococci,* Brevibacterium*, and* Candida *as well as* Propionibacterium* [3–8].

In the event of skin trauma from injuries such as burns, skin thinning, ulcers, scratches, skin defects, trauma, or wounds, the skin's defence may be compromised, allowing for microbial invasion of the epidermis resulting in anything from mild to serious infections of the skin. Common skin infections caused by microorganisms include carbuncles, furuncles, cellulitis, impetigo, boils* (Staphylococcus aureus)*, folliculitis* (S. aureus*,* Pseudomonas aeruginosa)*, ringworm (*Microsporum *spp.*, Epidermophyton *spp., and* Trichophyton *spp.), acne* (P*.* acnes)*, and foot odour (*Brevibacterium* spp.) [3, 8–11]. Environmental exposure, for example, in hospitals where nosocomial infections are prominent and invasive procedures make the patient vulnerable, may also create an opportunity for microbial infection. For example, with the addition of intensive therapy and intravascular cannulae,* S. epidermidis* can enter the cannula and behave as a pathogen causing bloodborne infections. Noninfective skin diseases such as eczema can also result in pathogenic infections by damaging the skin, thus increasing the risk of secondary infection by herpes simplex virus and/or* S. aureus* [5, 8, 12].

Skin infections constitute one of the five most common reasons for people to seek medical intervention and are considered the most frequently encountered of all infections. At least six million people worldwide are affected by chronic wounds and up to 17% of clinical visits are a result of bacterial skin infections and these wounds are a frequent diagnosis for hospitalised patients. These are experienced daily and every doctor will probably diagnose at least one case per patient. Furthermore, skin diseases are a major cause of death and morbidity [8, 13, 14]. The healing rate of chronic wounds is affected by bacterial infections (such as* S. aureus*,* E. coli*, and* P. aeruginosa*), pain, inflammation, and blood flow, and thus infection and inflammation control may assist in accelerating healing [15–17].

Topical skin infections typically require topical treatment; however, due to the ability of microbes to evolve and due to the overuse and incorrect prescribing of the current available conventional antimicrobials, there has been emergence of resistance in common skin pathogens such as* S. aureus* resulting as methicillin-resistant* Staphylococcus aureus* (MRSA) and other such strains. Treatment has therefore become a challenge and is often not successful [8, 18, 19]. In some regions of the world, infections are unresponsive to all known antibiotics [20]. This threat has become so severe that simple ulcers now require treatment with systemic antibiotics [21]. A simple cut on the finger or a simple removal of an appendix could result in death by infection. The World Health Organization (WHO) has warned that common infections may be left without a cure as we are headed for a future without antibiotics [22]. Therefore, one of the solutions available is to make use of one of the oldest forms of medicine, natural products, to treat skin infections and wounds [18, 23].

Complementary and alternative medicines (CAMs) are used by 60–80% of developing countries as they are one of the most prevalent sources of medicine worldwide [24–27]. Essential oils are also one of the most popular natural products, with one of their main applications being for their use in dermatology [28–30]. In fact, of all CAMs, essential oils are the most popular choice for treating fungal skin infections [13, 31]. Their use in dermatology, in the nursing profession, and in hospitals has been growing with great popularity worldwide, especially in the United States and the United Kingdom [1, 27, 32–35]. Furthermore, the aromatherapeutic literature [1, 2, 26, 32, 36–43] identifies numerous essential oils for dermatological use, the majority of which are recommended for infections. This brought forth the question as to the efficacy of commercial essential oils against the pathogens responsible for infections. The aim of this review was to collect and summarise the* in vivo*,* in vitro*, and clinical findings of commercial essential oils that have been tested against infectious skin diseases and their pathogens and, in doing so, offer aromatherapists and dermatologists valuable information regarding the effectiveness of essential oils for dermatological infections.

**Figure 1:**
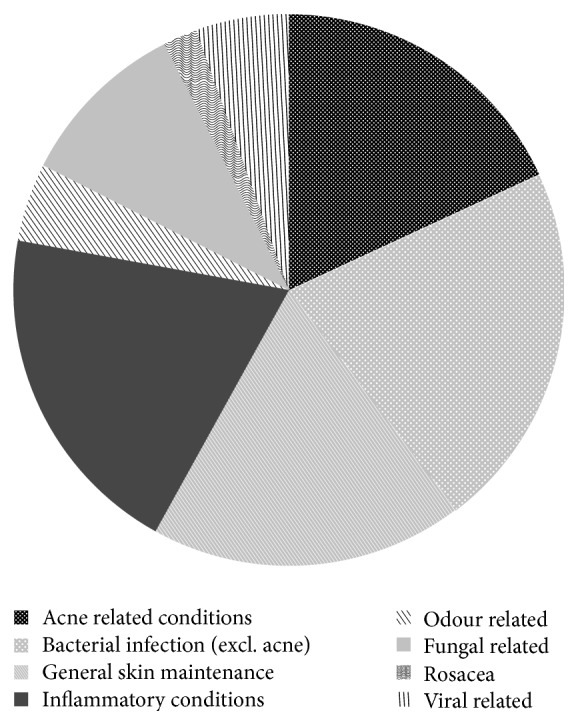
Summary of categorised dermatological conditions in which essential oils are used.

The readily available aromatherapeutic literature has reported over 90 (Table 1) commercial essential oils that may be used for treating dermatological conditions [1, 2, 26, 32, 36–43]. An overview of the skin related uses can be seen in Figure 1. Essential oils are mostly used for the treatment of infections caused by bacteria, fungi, or viruses (total 62%). This is followed by inflammatory skin conditions (20%) such as dermatitis, eczema, and lupus and then general skin maintenance (18%) such as wrinkles, scars, and scabs, which are the third most common use of essential oils. Other applications include anti-inflammatory and wound healing applications (Figure 1). Of the 98 essential oils recommended for dermatological use, 88 are endorsed for treating skin infections. Of these, 73 are used for bacterial infections, 49 specifically for acne, 34 for fungal infections, and 16 for viral infections.

## 2. Materials and Methods

### 2.1. Searching Strategy/Selection of Papers

The aim of the comparative review was to identify the acclaimed dermatological commercial essential oils according to the aromatherapeutic literature and then compare and analyse the available published literature. This will serve as a guideline in selecting appropriate essential oils in treating dermatological infections. The analysed papers were selected from three different electronic databases: PubMed, ScienceDirect, and Scopus, accessed during the period 2014–2016. The filters used included either “essential oils”, “volatile oils”, or “aromatherapy” or the scientific or common name for each individual essential oil listed in Table 1 and the additional filters “antimicrobial”, “antibacterial”, “skin”, “infection”, “dermatology”, “acne”, “combinations”, “fungal infections”, “dermatophytes”, “Brevibacteria”, “odour”, “antiviral”, “wounds”, “dermatitis”, “allergy”, “toxicity”, “sentitisation”, or “phototoxicity”.

### 2.2. Inclusion Criteria

In order to effectively understand the possible implications and potential of essential oils, the inclusion criteria were broad, especially with this being the first review to collate this amount of scientific evidence with the aromatherapeutic literature. Inclusion criteria included the following:

- Type of* in vitro* studies for bacterial and fungal pathogens by means of the microdilution assay, macrodilution assay, or the agar dilution assay
- *In vivo* studies
- Antiviral studies
- Case reports
- Animal studies
- All clinical trials

### 2.3. Exclusion Criteria

Papers or pieces of information were excluded for the following reasons:

- Lack of accessibility to the publication
- If the incorrect* in vitro* technique (diffusion assays) was employed
- Indigenous essential oils with no relevance to commercial oils
- If they were in a language not understood by the authors of the review
- Pathogens studied not relevant to skin disease

### 2.4. Data Analysis

The two authors (Ané Orchard and Sandy van Vuuren) conducted their own data extraction independently, after which critical analysis was applied. Information was extrapolated and recorded and comments were made. Observations were made and new recommendations were made as to future studies.

## 3. Results

### 3.1. Description of Studies

**Figure 2:**
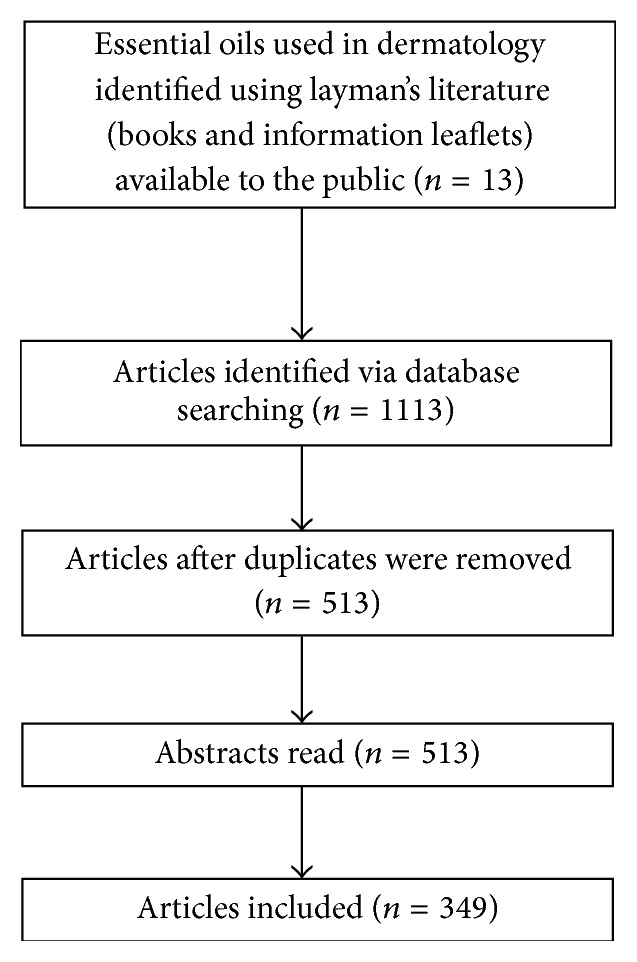
Flow diagram of the review approach.

After the initial database search, 1113 reports were screened. Duplicates were removed, which brought the article count down to 513, after which the abstracts were then read and additional reports removed based on not meeting the inclusion criteria. A final number of 349 articles were read and reviewed. Of these, 143 were* in vitro* bacterial and fungal studies (individual oil and 45 combinations), two* in vivo* studies, 15 antiviral studies, 19 clinical trials, and 32 toxicity studies. The process that was followed is summarised in Figure 2.

### 3.2. Experimental Approaches

#### 3.2.1. Chemical Analysis

Essential oils are complex organic (carbon containing) chemical entities, which are generally made up of hundreds of organic chemical compounds in combination that are responsible for the essential oil's many characteristic properties. These characteristics may include medicinal properties, such as anti-inflammatory, healing, or antimicrobial activities, but may also be responsible for negative qualities such as photosensitivity and toxicity [37].

Even with the high quality grade that is strived for in the commercial sector of essential oil production, it must be noted that it is still possible for essential oil quality to display discrepancies, changes in composition, or degradation. The essential oil composition may even vary between the same species [1, 44]. This may be due to a host of different factors such as the environment or location that the plants are grown in, the harvest season, which part of the plant was used, the process of extracting the essential oil, light or oxygen exposure, the storage of the oil, and the temperature the oil was exposed to [45–51].

Gas chromatography in combination with mass spectrometry (GCMS) is the preferred technique for analysis of essential oils [52]. This is a qualitative and quantitative chemical analysis method which allows for the assurance of the essential oil quality through the identification of individual compounds that make up an essential oil [1, 45, 53]. It has clearly been demonstrated that there is a strong correlation between the chemical composition and antimicrobial activity [51, 54, 55]. Understanding the chemistry of essential oils is essential for monitoring essential oil composition, which then further allows for a better understanding of the biological properties of essential oils. It is recommended to always include the chemical composition in antimicrobial studies [56].

### 3.3. Antimicrobial Investigations

Several methods exist that may be employed for antimicrobial analysis, with two of the most popular methods being the diffusion and the dilution methods [56–59].

#### 3.3.1. Diffusion Method

There are two types of diffusion assays. Due to the ease of application, the disc diffusion method is one of the most commonly used methods [60]. This is done by applying a known concentration of essential oil onto a sterile filter paper disc. This is then placed onto agar which has previously been inoculated with the microorganism to be tested, or it is spread on the surface. If necessary, the essential oil may also be dissolved in an appropriate solvent. The other diffusion method is the agar diffusion method, where, instead of discs being placed, wells are made in the agar into which the essential oil is instilled. After incubation, antimicrobial activity is then interpreted from the zone of inhibition (measured in millimetres) using the following criteria: weak activity (inhibition zone ≤ 12 mm), moderate activity (12 mm < inhibition zone < 20 mm), and strong activity (inhibition zone ≤ 20 mm) [24, 60–62].

Although this used to be a popular method, it is more suitable to antibiotics rather than essential oils as it does not account for the volatile nature of the essential oils. Essential oils also diffuse poorly through an aqueous medium as they are hydrophobic. Thus, the results are less reliable as they are influenced by the ability of the essential oil to diffuse through the agar medium, resulting in variable results, false negatives, or a reduction in antimicrobial activity [24, 63]. The results have been found to vary significantly when tested this way and are also influenced by other factors such as disc size, amount of compound applied to the disc, type of agar, and the volume of agar [57, 59, 64–68]. It has thus been recommended that results are only considered where the minimum inhibitory concentration (MIC) or cidal concentration values have been established [65].

#### 3.3.2. Dilution Methods

The dilution assays are reliable, widely accepted, and promising methods for determining an organism's susceptibility to inhibitors. The microdilution method is considered the “gold standard” [64, 68–70]. This is a quantitative method that makes it possible to calculate the MIC and allows one to understand the potency of the essential oil [68, 71]. With one of the most problematic characteristics of essential oils being their volatility, the microdilution technique allows for an opportunity to work around this problem as it allows for less evaporation due to the essential oil being mixed into the broth [67].

This microdilution method makes use of a 96-well microtitre plate under aseptic conditions where the essential oils (diluted in a solvent to a known concentration) are serially diluted. Results are usually read visually with the aid of an indicator dye. The microdilution results can also be interpreted by reading the optical density [72, 73]; however, the shortcoming of this method is that the coloured nature of some oils may interfere with accurate turbidimetric readings [74].

Activity is often classified differently according to the quantitative method followed. van Vuuren [56] recommended 2.00 mg/mL and less for essential oils to be considered as noteworthy, Agarwal et al. [75] regarded 1.00% and less, and Hadad et al. [76] recommended ≤250.00 *μ*g/mL. On considering the collection of data and frequency of certain MIC values, this review recommends MIC values of ≤1.00 mg/mL as noteworthy.

The macrodilution method employs a similar method to that of the microdilution method, except that, instead of a 96-well microtitre plate being used, multiple individual test tubes are used. Although the results are still comparable, this is a time-consuming and a tedious method, whereas the 96-well microtitre plate allows for multiple samples to be tested per plate, allowing for speed, and it makes use of smaller volumes which adds to the ease of its application [77, 78]. The agar dilution method is where the essential oil is serially diluted, using a solvent, into a known amount of sterile molten agar in bottles or tubes and mixed with the aid of a solvent. The inoculum is then added and then the agar is poured into plates for each dilution and then incubated. The absence of growth after incubation is taken as the MIC [79–81].

#### 3.3.3. The Time-Kill Method

The time-kill (or death kinetic) method is a labour intensive assay used to determine the relationship between the concentration of the antimicrobial and the bactericidal activity [82]. It allows for the presentation of a direct relationship in exposure of the pathogen to the antimicrobial and allows for the monitoring of a cidal effect over time [74]. The selected pathogen is exposed to the antimicrobial agent at selected time intervals and aliquots are then sampled and serially diluted. These dilutions are then plated out onto agar and incubated at the required incubation conditions for the pathogen. After incubation, the colony forming units (CFU) are counted. These results are interpreted from a logarithmic plot of the amount of remaining viable cells against time [74, 82, 83]. This is a time-consuming method; however, it is very useful for deriving real-time exposure data.

### 3.4. Summary of Methods

The variation in essential oil test methods makes it difficult to directly compare results [24, 58]. Numerous studies were found to employ the use of a diffusion method due to its acclaimed “ease” and “time saving” ability of the application. Researchers tend to use this as a screening tool whereby results displaying interesting outcomes are further tested using the microdilution method [84–87]. The shortcoming of this method is that firstly, due to the discussed factors affecting the diffusion methods, certain essential oils demonstrate no inhibition against the pathogen, and thus further studies with the oils are overlooked. Secondly, the active oils are then investigated further using the microdilution method. Therefore, the researchers have now doubled the amount of time required to interpret the quantitative data. Thirdly, the method may be believed to be a faster method if one considers the application; however, if one considers the preparation of the agar plates and their risk of contamination as well as the overall process of this method, there is very little saving of time and effort.

It is recommended to follow the correct guidelines as set out by the Clinical and Laboratory Standards Institute M38-A (CLSI) protocol [88] and the standard method proposed by the Antifungal Susceptibility Testing Subcommittee of the European Committee on Antibiotic Susceptibility Testing (AFST-EUCAST) [89] for testing with bacteria and filamentous fungi.

Other factors that may affect results and thus make it difficult to compare published pharmacological results of essential oils are where data is not given on the chemical composition, the microbial strain number, temperature and length of incubation, inoculum size, and the solvent used. The use of appropriate solvents helps address the factor of poor solubility of essential oils. Examples include Tween, acetone, dimethylformamide (DMF), dimethylsulfoxide (DMSO), and ethanol. Tween, ethanol, and DMSO have, however, been shown to enhance antimicrobial activity of essential oils [24, 53, 90]. Soković et al. [91] tested antimicrobial activity with ethanol as the solvent and Tween. When the essential oils were diluted with Tween, it resulted in a greater antifungal activity; however, Tween itself does not display its own antimicrobial activity [92]. Eloff [93] identified acetone as the most favourable solvent for natural product antimicrobial studies.

The inoculum is a representative of the microorganisms present at the site of infection [94]. When comparing different articles, the bacterial inoculum load ranges from 5 × 10^2^ to 5 × 10^8^ CFU/mL. The antibacterial activity is affected by inoculum size [62, 95–99]. If this concentration is too weak, the effect of the essential oils strengthens; however, this does not allow for a good representation of the essential oil's activity. If the inoculum is too dense, the effect of the essential oil weakens and the inoculum becomes more prone to cross contamination [100]. Future studies should aim to keep the inoculum size at the recommended 5 × 10^6^ CFU/mL [99].

## 4. Pathogenesis of Wounds and Skin Infections and the Use of Essential Oils

The pathogenesis of the different infections that are frequently encountered in wounds and skin infections is presented in Table 2. A more in-depth analysis of essential oils and their use against these dermatological pathogens follows.

### 4.1. Gram-Positive Bacteria

The Gram-positive bacterial cell wall is comprised of a 90–95% peptidoglycan layer that allows for easy penetration of lipophilic molecules into the cells. This thick lipophilic cell wall also results in essential oils making direct contact with the phospholipid bilayer of the cell membrane which allows for a physiological response to occur on the cell wall and in the cytoplasm [183, 184].

#### 4.1.1. *Staphylococcus aureus*

*Staphylococcus aureus* is a common Gram-positive bacterium that can cause anything from local skin infections to fatal deep tissue infections. The pathogen is also found colonising acne and burn wounds [185–187]. Methicillin-resistant* S. aureus* (MRSA) is one of the most well-known and widespread “superbugs” and is resistant to numerous antibiotics [158]. Methicillin-resistant* S. aureus* strains can be found to colonise the skin and wounds of over 63%–90% of patients and have been especially infamous as being the dreaded scourge of hospitals for several years [22, 188–190].* Staphylococcus aureus* has developed resistance against erythromycin, quinolones, mupirocin, tetracycline, and vancomycin [190–192].

Table 3 shows some of the antimicrobial* in vitro* studies undertaken on commercial essential oils and additional subtypes against this most notorious infectious agent of wounds. Of the 98 available commercial essential oils documented from the aromatherapeutic literature for use for dermatological infections, only 54 oils have been tested against* S. aureus* and even fewer against the resistant* S. aureus* strain. This is troubling, especially if one considers the regularity of* S. aureus* resistance. It should be recommended that resistant* S. aureus* strains always be included with every study.

When considering the antimicrobial activity of the tested essential oils, it can be noted how the main compounds influence overall antimicrobial activity.* Melaleuca alternifolia *(tea tree), rich in terpinen-4-ol, showed noteworthy activity, and* Anthemis aciphylla* var.* discoidea* (chamomile) containing *α*-pinene and terpinen-4-ol displayed noteworthy activity (1.00 mg/mL) [114], whereas the essential oil predominantly containing terpinen-4-ol displayed an MIC value of 0.50 mg/mL. The* Origanum *spp. (*Origanum scabrum* and* Origanum vulgare*) were shown to display rather impressive antimicrobial activity, which appeared to predominantly be related to the amount of carvacrol [163]. Geraniol also appears to be a compound that influences antimicrobial activity against the* staphylococci* spp. as can be seen for* Backhousia citriodora* (lemon myrtle) and* Cymbopogon martinii* (palmarosa) (geraniol 61.6%) [125, 126].* Cymbopogon martinii*, with lower levels of geranial (44.80%), showed moderate antimicrobial activity [99].* Mentha piperita* (peppermint) had higher antimicrobial activity for oils with higher concentrations of menthol [128, 155].* Laurus nobilis* (bay),* Styrax benzoin*, and* Cinnamomum zeylanicum* (cinnamon), each rich in eugenol, were found to have notable activity [99].

It is interesting to consider the essential oils investigated and to compare them to what is recommended in the aromatherapeutic literature. For example,* Lavandula angustifolia *(lavender) is recommended for abscesses, carbuncles, and wounds [2, 26, 32, 36–43], which all involve* S. aureus*; however,* in vitro* activity was found to discount this oil as an antimicrobial [99, 136, 139, 140]. The same could be said about essential oils such as* Achillea millefolium *(yarrow) [112],* Anthemis nobilis* (Roman chamomile) [99],* Boswellia carteri *(frankincense) [116],* Citrus aurantifolia* (lime) [80],* Foeniculum vulgare *(fennel) [132, 133], and* Melissa officinalis *(lemon balm) [139].

Some clinical studies included the evaluation of the effects of essential oils on malodorous necrotic ulcers of cancer patients. The use of an essential oil combination (mostly containing* Eucalyptus globulus *(eucalyptus)) resulted in a decrease in inflammation, reduction of the odour, and improved healing rates [193]. Edwards-Jones et al. [194] performed a clinical study with a wound dressing containing essential oils to decrease infection risk. Ames [195] found* Melaleuca alternifolia* (tea tree) to be effective in treating wounds; and* Matricaria recutita *(German chamomile) with* L. angustifolia *at a 50 : 50 ratio diluted in calendula oil was found to improve leg ulcers and pressure sores.

Methicillin-resistant* S. aureus* hinders the rate of wound healing, which may lead to chronic wounds [196]. Delayed wound healing has been proven to lead to psychological stress and social isolation [197, 198]. A randomised controlled trial, consisting of 32 patients (16 in control group, 16 in placebo group) with stage II and above MRSA-colonised wounds that were not responding to treatment, was undertaken where the control group was treated with a 10% topical* M. alternifolia *preparation and was found to effectively decrease colonising MRSA in 87.5% of patients and result in a 100% healing rate within 28 days [196]. These studies lead to the high recommendation of the incorporation of this essential oil combination in palliative care.

Methicillin-resistant* S. aureus* may potentially be carried and propagated by hospital staff and patients, which is an acknowledged risk for hospital-acquired infections [147, 189]. Therefore, successful decolonisation of MRSA from patients and good hygiene may improve the microbial load, number of reinfections, and ultimately therapeutic outcomes of patients [199]. A topical preparation containing* M. alternifolia *essential oil has been considered for assistance in eradicating MRSA in hospitals, due to its reported efficacy [200]. The largest randomised trial against MRSA colonisation included 224 patients where the control group was treated with 2% nasal mupirocin applied three times a day, 4% chlorhexidine gluconate soap used at least once a day, and 1% silver sulfadiazine cream applied to skin infections once a day. The study group was treated with 10%* M. alternifolia* oil nasal cream applied three times a day and 5%* M. alternifolia *oil body wash used at least once daily with a 10%* M. alternifolia *cream applied to skin infections. The results showed that 41% of patients in the study group were cleared as opposed to 49% of patients on the standard therapy [200]. A small three-day pilot study was designed by Caelli et al. [189] to observe whether daily washing with a 5%* M. alternifolia *oil would clear MRSA colonisation which may result in ICU patient outcome improvement [199]. The test group made use of 4%* M. alternifolia *nasal ointment and 5%* M. alternifolia *oil body wash and was compared to a conventional treatment consisting of 2% mupirocin nasal ointment and triclosan body wash. The test group overall was found to have more improvement at the infection site when compared to the control group. Although the pilot study was too small to be statistically significant, the researchers did find that the* M. alternifolia *oil performed better than the conventional treatment and was effective, nontoxic, and well tolerated [189]. Messager et al. [90] tested 5%* M. alternifolia *ex vivo in a formulation, where it again was proven to decrease the pathogenic bacteria on the skin. In another study,* M. alternifolia* oil was investigated to determine the influence on healing rates [201]. The patients were treated with water-miscible tea tree oil (3.30%) solution applied as part of the wound cleansing regimen. This study used this oil as a wash only three times a week which is not how this oil is prescribed and hence the results were not positive. A more accurate method of study was shown by Chin and Cordell [202], where* M. alternifolia* oil was used in a dressing for wound healing abilities. All patients, except for one, were found to have an accelerated healing rate of abscessed wounds and cellulitis. The concluding evidence shows that there is definitely potential for the use of* M. alternifolia* (tea tree) oil as an additional/alternative treatment to standard wound treatments [203].

The healing potential of* Commiphora guidotti* (myrrh) was investigated via excisions of rats. The authors could confidently report on an increased rate in wound contraction and candid wound healing activity that was attributed to the antimicrobial and anti-inflammatory effects of this oil [204].* Ocimum gratissimum* (basil) was also found by Orafidiya et al. [205] to promote wound healing by eradicating the infectious pathogens and by inducing early epithelialisation and moderate clotting formation, thereby accelerating scab formation, contraction, and granulation.

From these studies, clearly,* M. alternifolia *has shown great promise against* S. aureus*. However, considering the potential of essential oils in clinical practice and comparing them to essential oils with promising* in vitro* activity, other oils such as* Cymbopogon citratus *(lemongrass),* Santalum album* (sandalwood), and* Vetiveria zizanioides/Andropogon muricatus *(vetiver) should in the future be paid the same amount of attention.

#### 4.1.2. Pathogens Involved in Acne

Pathogens associated with acne include* Propionibacterium acnes*,* Propionibacterium granulosum*, and* Staphylococcus epidermidis* [206–208]. Methicillin-resistant* S. epidermidis *(MRSE) have become extensively problematic microorganisms in the recent years due to their antimicrobial resistance and* P. acnes *has developed resistance to tetracycline, erythromycin, and clindamycin. Both have also shown multidrug resistance, including against quinolones [158, 188, 206].  Table 4 displays the* in vitro* antimicrobial efficacies of commercial essential oils against bacteria involved in the pathogenesis of acne. When observing the number of commercial essential oils that are recommended for acne treatment, less than half of the commercial oils have actually focused on* S. epidermidis*,* P. granulosum*, and* P. acnes*. Overall, the acne pathogens have been sorely neglected in essential oil studies.

For* Anthemis aciphylla* var.* discoidea* (chamomile) 0.13–0.25 mg/mL, initially, it appeared that higher *α*-pinene and lower terpinen-4-ol showed higher antimicrobial activity. However, the sample with terpinen-4-ol predominantly as its main component displayed the best activity at 0.06 mg/mL. This makes *α*-pinene appear as an antimicrobial antagonist.* Cinnamomum zeylanicum*,* Rosa centifolia* (rose),* L. angustifolia*, and* Syzygium aromaticum *(clove) displayed noteworthy antimicrobial activity against both* S. epidermidis *and* P. acnes.* Only the latter two are, however, recommended in the aromatherapeutic literature for the treatment of acne.* Leptospermum scoparium* (manuka) showed noteworthy activity for both* P. acnes* and* S. epidermidis*; however, Tween 80 was used as a solvent, which may overexaggerate the antimicrobial activity. Another study also found* L. scoparium* to effectively inhibit* P. acnes*. As was seen against* S. aureus*,* O. scabrum* and* O. vulgare *also notably inhibited* S. epidermidis*. Unfortunately, these oils were not studied against* P. acnes*.* Cymbopogon citratus* was shown to effectively inhibit* P. acnes*; however, no data was available against* S. epidermidis*. Essential oils such as* S. album, V. zizanioides, Viola odorata *(violet),* Citrus aurantium *var.* amara *(petitgrain), and* Citrus bergamia *(bergamot) are a few that are recommended for the treatment of acne and other microbial infections [2, 26, 32, 36, 37, 40–43] in the aromatherapeutic literature that are yet to be investigated.

Some clinical studies have shown promising results. A four-week trial comparing* O. gratissimum* oil with 10% benzoyl peroxide and a placebo was conducted and was aimed at reducing acne lesions in students. The 2% and 5%* O. gratissimum* oils in the hydrophilic cetomacrogol base were found to reduce acne lesions faster than standard therapy, and they were well tolerated. The 5% preparation, despite being highly effective, caused skin irritation. Overall,* O. gratissimum* oil showed excellent potential in the management of acne as it was as effective as benzoyl peroxide, although it was less popular with patients due to the unpleasant odour [217].

*Melaleuca alternifolia *oil demonstrated* in vitro* antimicrobial and anti-inflammatory activity against* P. acnes* and* S. epidermidis* and is in fact the essential oil on which most clinical trials have been undertaken. Bassett et al. [218] performed one of the first rigorous single-blinded randomised (RCT) controlled trials consisting of 124 patients that assessed the efficacy of 5%* M. alternifolia *gel in comparison to 5% benzoyl peroxide lotion in the management of mild to moderate acne. Both treatments showed equal improvement in the acne lesions. Enshaieh et al. [219] evaluated the efficacy of 5%* M. alternifolia *on mild to moderate acne vulgaris. The 5%* M. alternifolia *oil was found to be effective in improving the number of papules in both inflammatory and noninflammatory acne lesions and was found to be more effective than the placebo. Proven efficacy has made* M. alternifolia *preparations popular in acne products.

Other oil studies included a gel formulation containing acetic acid,* Citrus sinensis* (orange), and* Ocimum basilicum* (sweet basil) essential oils, which was tested in acne patients. The combination of these antimicrobial essential oils and the keratolytic agent resulted in a 75% improvement in the rate of acne lesion healing [220].

If one examines the results displayed in Table 4, essential oils such as* Anthemis aciphylla* var.* discoidea *(chamomile),* C. zeylanicum*,* Citrus aurantium *(bitter orange),* O. vulgare *(oregano), and* S. aromaticum *displayed higher antimicrobial activity* in vitro* than* M. alternifolia*, yet these essential oils have to be investigated clinically.

#### 4.1.3. Gram-Negative Bacteria

The Gram-negative bacterial cell wall consists of a 2-3 nm thick peptidoglycan layer (thinner than Gram-positive bacteria), which means that the cell wall consists of a very small percentage of the bacteria. The cell wall is further surrounded by an outer membrane (OM) which is comprised of a double layer of phospholipids that are linked to an inner membrane by lipopolysaccharides (LPS). This OM protects the bacteria from lipophilic particles; however, it makes them more vulnerable to hydrophilic solutes due to the abundance of porin proteins that serve as hydrophilic transmembrane channels [184, 221, 222].

Gram-negative pathogens present a serious threat with regard to drug resistance, especially* Escherichia coli* and* Pseudomonas aeruginosa* [190, 192]. These pathogens that are found to colonise wounds often cause multidrug resistance [166, 223]. *β*-Lactamase-positive* E. coli* is appearing frequently among nonhospital patients [224].* Pseudomonas aeruginosa* is a regular cause of opportunistic nosocomial infections [187]. It is often involved in localised skin infections, green nail syndrome, and interdigital infection, colonises burn wounds, and may expand into a life-threatening systemic illness [225].

A number of essential oils display antimicrobial activity against* E. coli *and* P. aeruginosa *with the predominant studies having been done against* E. coli *(Table 5). The Gram-negative pathogens appear to be a lot more resistant to essential oil inhibition than the Gram-positive bacteria, but this a known fact.

*Aniba rosaeodora* (rosewood) was found to inhibit* E. coli* at an MIC value of 0.40 mg/mL. No GC-MS data was given [85].* Anthemis aciphylla* var.* discoidea *(chamomile) also displayed notable inhibition against* E. coli* and* P. aeruginosa*; however, the highest activity was seen for the essential oil containing high levels of *α*-pinene (39.00%) and terpinen-4-ol (32.10%) [114].* Cinnamomum zeylanicum*, with the main compound cinnamaldehyde, was shown to have inhibited these two Gram-negative pathogens at noteworthy MIC values [80]. Noteworthy activity was also reported for* Commiphora myrrha *(myrrh) and* Thymus numidicus *(thyme) [99].* Syzygium aromaticum* and* S. album* were reported to effectively inhibit* P. aeruginosa* [99]; and* Thymus vulgaris *(thyme) inhibits* E. coli *(including multidrug-resistant strains) [182].

#### 4.1.4. Other Bacterial Pathogens

*Brevibacterium* spp. form part of the Coryneform bacteria and are involved in foul body odour [3, 103]. Insufficient quantitative studies have been conducted using commercial essential oils to treat problems caused by these microorganisms, even though there have been some earlier studies using the diffusion assays against* B. linen* [226–228]. One quantitative study reported on the activity of* Ziziphora persica* against* B. agri *(125 *μ*g/mL) and* B. brevis *(250 *μ*g/mL), in addition to* Ziziphora clinopodioides *against* B. agri *(31.25 *μ*g/mL) and* B. brevis *(125 *μ*g/mL) [229]. In another study, essential oils of* Kunzea ericoides* (Kānuka) and* L. scoparium* were able to inhibit three species of* Brevibacterium* (MIC: 0.06–1.00 mg/mL) [138]. Clearly, the lack of attention to this neglected group of microorganisms warrants further attention, especially considering that, to the best of our knowledge, not one essential oil recommended for odour has been investigated against relevant pathogens* in vitro*.

The *β*-hemolytic* Streptococcus (S. pyogenes)* is a threatening pathogen that needs to be considered when investigating wound infections [166]. Group A* Streptococcus *(GAS) is usually involved in impetigo and necrotising fasciitis (“flesh-eating” disease). This pathogen has developed resistance to erythromycin, azithromycin, clarithromycin, clindamycin, and tetracycline [188, 190]. Group B* Streptococcus *is also involved in skin infections and has developed resistance to clindamycin, erythromycin, azithromycin, and vancomycin [190]. Periorbital cellulitis is a common occurrence in children and is caused by* Haemophilus influenzae *[106], and* Clostridium* spp. (*C. perfringens, C. septicum, C. tertium, C. oedematiens*, and* C. histolyticum*) are involved in gas green/gangrene infections.  Table 6 summarises the antimicrobial activity of essential oils that have been studied and shown to have some* in vitro* efficacy against these pathogens. The lack of studies against* S. pyogenes*,* C. perfringens*, and* H. influenza* highlights the need to investigate these sorely neglected dermatologically important pathogens, especially since the few available studies have shown these organisms to be highly susceptible to essential oil inhibition. These are also pathogens that cause deeper skin infections, so, with the enhanced penetration offered by essential oils, they may prove beneficial.

#### 4.1.5. Fungal Infections: Yeasts

Yeasts may act as opportunistic pathogens and can result in infection if presented with the opportunity, the most common pathogen being* Candida albicans*.* Candida* spp. can cause candidiasis at several different anatomical sites [230].* Candida *has started developing resistance to first-line and second-line antifungal treatment agents such as fluconazole [190]. Essential oils demonstrating noteworthy activity against this organism are shown in Table 7.* Candida albicans* has been quite extensively investigated and most oils used in dermatology have been tested against this pathogen.

*Cymbopogon citratus*,* C. martinii*,* L. nobilis*,* M. piperita*,* P. graveolens*,* Santolina chamaecyparissus *(santolina), and* Thymus *spp. are essential oils recommended in the aromatherapeutic literature for the treatment of fungal infections that have* in vitro* evidence confirming the effectiveness as antifungals.* Cananga odorata* (ylang-ylang),* Cinnamomum cassia* (cinnamon),* C. zeylanicum*,* Coriandrum sativum *(coriander),* Cymbopogon nardus* (citronella),* Matricaria chamomilla *(German chamomile), and* S. benzoin* also displayed* in vitro* noteworthy activity; however, these are interestingly not recommended in the aromatherapeutic literature.

In an* in vivo* study,* L. angustifolia* was found to effectively inhibit growth of* C. albicans* isolated from 20 patients, which was comparative to the inhibition observed by clotrimazole [272].

#### 4.1.6. Fungal Infections: Dermatophytes

Infection with these organisms results in dermatophytosis, which affects the skin, nails, or hair [230, 273, 274]. There is a 10–20% risk of a person acquiring a dermatophyte infection [29], and although the symptoms do not necessarily pose a threat, the treatment is costly and onerous due to resistance and side effects [29]. Essential oils present an excellent option for treating superficial human fungal infections, especially when one is confronted with the effective antifungal results found in previous studies (Table 8). This is encouraging considering the difficulty and challenges faced in treating these infections.

The ability of topical formulations to penetrate the skin is crucial for the effective treatment of subcutaneous infections [108].* Melaleuca alternifolia *oil has displayed* in vitro* activity against* M. mycetomatis *and* M. furfur*, proving its potential in treating eumycetoma, pityriasis, and seborrheic dermatitis, not only because of its antifungal activity, but also because of its ability to penetrate the skin due to its main compound (terpinen-4-ol) [108, 109, 275, 276].

Onychomycosis is generally resilient to topical treatment of any kind; thus, there is a poor cure rate. It is usually treated systemically due to its infrequency in responding to topical treatments [277, 278]. With onychomycosis being the most frequent cause of nail disease, Buck et al. [279] aimed to treat onychomycosis in clinical trials whereby 60% of patients were treated with* M. alternifolia *oil and 61% of patients were treated with 1% clotrimazole. There was only a 1% difference between the two study groups. What would be interesting for future studies is to determine what the results would be when testing the same treatments against resistant strains.

Tinea pedis is often treated topically, which presents an opportunity for essential oil use [280].* Melaleuca alternifolia* oil was evaluated in two trials for treating tinea pedis. In the first trial by Tong et al. [281], the patients were treated with either a 10%* M. alternifolia *oil in sorbolene, 1% tolnaftate, or a placebo (sorbolene). The patients on* M. alternifolia *oil treatment had a mycological cure rate of 30%. Mycological cure rates of 21% were seen in the placebo group and of 85% in patients receiving tolnaftate, proving the essential oil to not be as effective. The second trial tested two solutions of 25% and 50%* M. alternifolia *oil in ethanol and polyethylene glycol. This was compared to a placebo containing only the vehicle in a double-blinded randomised controlled trial [282]. The placebo group showed a clinical response in 39% of patients.* Melaleuca alternifolia *oil test groups showed a 72% improvement. A higher concentration of the oil is thus required for treating this type of infection.

In spite of the dermatophytes showing susceptibility to essential oils, there are few studies dedicated to these pathogens. One would expect more essential oil treatments considering the difficulty in treating these infections which require expensive prolonged treatment. An essential oil with superior activity certainly warrants further investigation, particularly as essential oils work well on skin surfaces and are shown to display good penetration capabilities [283, 284].* Madurella mycetomatis *and* Malassezia furfur* are sorely neglected pathogens in research. Possibly their fastidious nature acts as a barrier for further research. As far as clinical studies are concerned, essential oils against fungal pathogens have also been neglected. Only* M. alternifolia *oil has been clinically studied extensively with investigations incorporating onychomycosis, tinea pedis, and dandruff [275, 279, 281, 282, 285]. It would be interesting to observe the antidermatophytic property of essential oils that have shown to be noteworthy* in vitro* antifungal activity such as for* Apium nodiflorum* (celery),* Cedrus atlantica *(cedar wood),* C. citratus*,* Juniperus oxycedrus* ssp.* oxycedrus* (cade),* Pelargonium graveolens *(geranium),* S. aromaticum*, and* Thymus *spp.

## 5. Essential Oil Combinations

Other than the use of oils within carrier oils, most essential oils are used in blends or combinations of two or more oils [32]. These blends are considered to be an art where the oils are carefully selected and combined with the intention of holistically healing the “whole” individual according to his/her symptoms. The goal of blending is to create a synergistic therapeutic effect where the combination of essential oils is greater than the sum of the individual oil [37, 40, 286]. The beneficial value of synergy has been notorious and used since antiquity [74]. Synergy can be achieved if the compounds in the oil are able to affect different target sites, or they may interact with one another to increase solubility thereby enhancing bioavailability [287–289]. Mechanisms that can lead to pharmacological synergy are (1) multitarget effect where multiple target sites of the bacterial cell are affected; (2) solubility and bioavailability enhancement; (3) the mechanism where the essential oil may inhibit the mutation mechanism of bacteria to the antimicrobial; or (4) the mechanism where the essential oil may inhibit the efflux pump of bacteria, thus allowing for the antimicrobial to accumulate inside the bacteria [11, 288, 290]. The goal is for a multitargeted treatment to decrease pathogen mutation and thus retard the development of resistance. The combined formulation also has the potential to decrease toxicity and adverse side effects by lowering the required dose [290–292]. This is not an infallible method, however, as even the combined penicillin with clavulanic acid has become prone to resistance [293, 294].

When blends are created, the intention is to create therapeutic synergy [2, 26, 32]. The reasoning for the combinations is to produce a forceful blend that has more than one mode of action. For example, in the treatment of abscesses,* C. bergamia *and* L. angustifolia* may be used in combination.* C. bergamia *is used for its antiseptic properties and* L. angustifolia* for antiseptic and anti-inflammatory effects.* Anthemis nobilis* is also often used for anti-inflammatory effects [2, 26, 32, 37]. The theory is sound and not too far off considering that numerous essential oils have been proven to possess additional pharmacological properties. For example,* P. graveolens* is known for antiseptic and anti-inflammatory properties. It is often used for the ability to balance sebum secretions and clear oily and sluggish skin [295].* Eucalyptus globulus *(eucalyptus) may be used for its proven antimicrobial and anti-inflammatory activity [296, 297]. Often used on acne prone skin because of its antiseptic properties is* L. angustifolia* [298, 299].* Anthemis nobilis* is believed to ease inflammation and* L. angustifolia* assists with healing and regeneration [25].* Citrus aurantium *(neroli) flower oil has displayed antioxidant activity [120], and the main component of* M. alternifolia *(terpinen-4-ol) has the ability to hinder tumour necrosis factor (TNF), interleukin-1, interleukin-8, and interleukin-10, and prostaglandin E_2_ [300]. The anti-inflammatory activity of* C. bergamia *has been proven by several studies* in vitro* or on animal models [301, 302]. This supports the theory behind therapeutic synergy; however, the mistaken belief that any essential oil blend will result in synergy is not fully accurate [33]. It is a complex area, because although a certain combination may have a synergistic therapeutic effect, it does not necessarily translate into antimicrobial synergy and this needs further investigation.

By reviewing the aromatherapeutic literature [1, 2, 26, 32, 36–43], at least 1500 possible combinations (made up of two oils) could be identified for dermatology alone. This brings forth the question as to the antimicrobial effect of the overall combination. After all, if essential oils are to be investigated as options to curb antimicrobial resistance, the aim of combination therapy should be to broaden the spectrum of the antimicrobial activity and prevent development of additional resistance occurring [96]. The risk of resistance emerging against essential oils should not be disregarded because suboptimal doses of essential oils may impact these phenomena [303]. Sublethal concentration exposure to* M. alternifolia *has been proven to result in slightly lowered bacterial susceptibility to* M. alternifolia *and a larger decrease in susceptibility to conventional antimicrobials. The study concluded that essential oil products containing sublethal concentrations may result in stress-hardened (mutated)* S. aureus* isolates and possible treatment failure [146]. This highlights that although therapeutic synergy is strived for, these must still be verified in a controlled environment [288].

Studies have proven that essential oils, whether in combination with other essential oils [99] or in combination with conventional antimicrobials [304], can initiate a synergistic antimicrobial effect. This effect, however, is limited to the studied pathogen [290]. de Rapper et al. [99] demonstrated that even when essential oils displayed synergistic blends against one pathogen, the same could not be said against other pathogens. This highlights how the assumption should not be made that all synergistic blends are the same against all pathogens.

The fractional inhibitory concentration index (∑FIC or FICI) is the commonly accepted mathematical method employed to interpret interactions in 1 : 1 combinations [74]. ∑FIC is determined from the sum of all individual FICs of each of the test agents within the combination [305]. This then allows for the determination of their individual interactions in the combination [306]. The results are interpreted as synergistic (∑FIC ≤ 0.5), additive (∑FIC > 0.5–1.0), indifferent ∑FIC (>1.0 ≤ 4.0), or antagonistic (∑FIC > 4.0) [74]. Although using ∑FIC calculations is an easy method, it is not without its limitation. When examining 1 : 1 ratios between two essential oils, it is assumed that half the concentration will only offer half the effect. This is not necessarily the case between agents, as two agents may not have the same dose response at the same concentrations [307]. An interactive assessment of the different ratio combinations is mostly carried out using the isobole method [308, 309]. This method allows for more accurate valuation of the combination contribution made by each agent on a mathematical level line where all points are collected on a surface that lies at a specific value [288, 305, 310]. There are, however, other complex methods that can also be used [311, 312].

### 5.1. Essential Oils in Combination with Other Essential Oils

Although combinations are frequently mentioned in aromatherapy to treat skin ailments, only a handful of studies documenting essential oil combinations were found against skin pathogens (Table 9). The combination studies are predominantly limited to* S. aureus*,* P. aeruginosa, C. albicans*, and, to a lesser extent,* E. coli*. Even fewer studies were found against the dermatophytes and acne pathogens. This is rather abysmal considering the amount of combinations and the regularity of their use. An interesting observation was made even in an early study [316], where it was shown that synergy found in the 1 : 1 combinations was apparent irrespective of the poor efficacy displayed by the individual oils. This indicates that essential oils do not necessarily have to be combined based purely on independent noteworthy antimicrobial activity.

One of the largest studies on combinations was done by de Rapper et al. [99], where 45 essential oils were combined with* L. angustifolia*, which is one of the most popular essential oils used in combination. What could be observed was that there was no predictive pattern as to what the combined FIC index would be. There were a few synergistic interactions, most of which against* C. albicans *and some antagonism; however, the majority of the combinations resulted in an indifferent or additive interaction. A study investigated the antimicrobial activity of the popular commercial product containing essential oils (Olbas). The individual essential oils were tested separately and then in the combined product [128]. The combination of the four oils showed no further enhancement in the antimicrobial. The combination of* Syzygium aromaticum *(clove) and* Rosmarinus officinalis *(rosemary) has also displayed synergy against* C. albicans*, at ratios of 1 : 5, 1 : 7, and 1 : 9 [169]. Synergy was observed with a combination of commercially popular* L. angustifolia* and* M. alternifolia *essential oils against dermatophytes* T. rubrum* and* T. mentagrophytes *var.* interdigitale* in various combinations [303]. Unfortunately, only a few essential oil combinations have been investigated in clinical settings.

Essential oil combinations have proven efficacy in clinical settings.* L. angustifolia* and* Matricaria recutita* (German chamomile) were investigated in a small trial involving eight patients with chronic leg ulcers. Five received a 6% mixture of the two essential oils mixed in* Vitis vinifera* (grape seed) carrier oil, and three received conventional wound care. It was noted that four of the five patients in the control group had complete healing of the wounds with the fifth patient making progress towards a recovery [317]. Another successful essential oil combination included* L. angustifolia*,* Artemisia vulgaris* (mugwort), and* Salvia officinalis* (sage) in treating chronic wounds such as venous ulcers, pressure sores, skin tears, and abrasions. It was speculated that the essential oils had increased circulation and vascular permeability resulting in accelerated angiogenesis [318]. An* in vivo* study by Mugnaini et al. [266] made use of a mixture composed of 5%* O. vulgare*, 5%* R. officinalis*, and 2%* Thymus serpyllum *(Breckland thyme), diluted in* Prunus dulcis* (sweet almond), and this was topically administered on* M. canis* lesions. A 71% success rate in treatment was observed.

### 5.2. Essential Oils in Combination with Conventional Antimicrobials

In an effort to prevent resistance and increase antimicrobial efficacy against multidrug-resistant bacteria, the combination of essential oils with antibiotics has been investigated [182, 319–321]. Certain studies are based on the assumption that the antimicrobial and essential oils attack at different sites of the pathogen [304], while others believe this is due to the increase in chemical complexity, together with the added advantage of enhanced skin penetration by the essential oil components [322], or the hope that the essential oils will improve antibiotic diffusion across the bactericidal cell membranes and/or inhibit the Gram-negative efflux pump [323]. Conventional medication in combination with essential oils (bought over the counter or shelves) is also common among patients [183]; therefore, unknowingly, they may be causing enhancement or failure.

Table 10 displays the studies validating the improvement of antimicrobial activity from the combined use of antimicrobials with essential oils. The majority of the studies have shown essential oils to enhance antimicrobial activity of antibiotics and antifungals [81, 346, 347].* Origanum vulgare* oil displayed synergy (FICs 0.4–0.5) when combined with doxycycline, florfenicol, or sarafloxacin against an ESBL producing* E. coli* [324]. This presents a possible solution for *β*-lactamase antibiotic-resistant bacteria.* Origanum vulgare* essential oils were investigated and shown to improve the activity of *β*-lactam antibiotics against both Gram-positive and Gram-negative *β*-lactamase-producing bacteria [77, 324].* Helichrysum italicum* (everlasting) (2.5%) reduced the multidrug resistance of Gram-negative bacteria,* E. coli* and* P. aeruginosa*, to chloramphenicol [320].

Four community-associated methicillin-resistant* S. aureus* (CA-MRSA) isolates were used to compare benzethonium chloride 0.2% with* M. alternifolia *and* T. vulgaris* combination with conventional antimicrobials (neomycin with polymyxin B sulphate and polymyxin B sulphate with gramicidin). The essential oil-antibiotic combination was found to be more effective than conventional medicines on their own [348]. In another study, however, where* M. piperita*,* M. alternifolia, T. vulgaris*, and* R. officinalis* were each individually combined with amphotericin B against* C. albicans*, antagonism was observed [304], indicating that there may still be risks present when combining essential oils with antimicrobials.* Cinnamomum cassia *showed potentiation of amphotericin B activity against* C. albicans*. The increased activity was attributed to the essential oil because synergy increased with an increase in essential oil concentration; however, antagonism was observed for combinations with a lower concentration of essential oil [238].

Although there have been some studies* in vitro* on essential oil combinations with antibiotics and antifungals, little attention has been paid to* in vivo* studies or clinical trials. Syed et al. [285] tested a 2% butenafine hydrochloride combination with a 5%* M. alternifolia *oil cream in a clinical trial, consisting of 60 patients, treating toenail onychomycosis. The control group showed an 80% cure rate compared to 0% by the placebo group containing* M. alternifolia *alone, allowing the study to conclude clinical effectiveness of butenafine hydrochloride and* M. alternifolia *in combination. However, in order to determine whether the same could be said for butenafine, a control group should have also been allowed for this product to allow for comparison.

## 6. Antiviral Studies

Viral infections are a worldwide threat, firstly due to the lack of effective treatments available and secondly due to resistance [333]. Essential oils are a potential source for novel medicines in this regard [30]. Certain essential oils have previously displayed antiviral activity [30, 334], with the best viral inhibitors specifically acting on the steps involved in viral biosynthesis. These work by inhibiting viral replication, thereby limiting viral progeny production [30]. It is advantageous that the viral replication cycle consists of a complex sequence of different steps because it increases the chance of interference from antiviral agents [30].

Less than half of the essential oils recommended for skin infections have been studied for antiviral activity.  Table 11 records the readily available studies. The most studied virus is the herpes simplex virus (HSV) and the most studied essential oil is* M. alternifolia*.

Antiviral studies encompass an extensive process where the cytotoxicity and antiviral activity need to be determined. Antiviral activity is usually tested via the plaque reduction assay on* Vero* (African green monkey kidney cells) cells infected with the virus. This assay determines the effective concentration inhibiting 50% of virus growth (IC_50_). The selective indicator or selectivity index is calculated with the equation of CC_50_/IC_50_. An essential oil with a SI value greater than four is considered suitable as an antiviral agent [332, 333]. Besides the criteria being made for the SI, no criteria for the IC_50_ have been made. According to the results reviewed, an IC_50_ value of less than 0.0010% or 1.00 *μ*g/mL should be considered as noteworthy.

Essential oils recommended in the aromatherapeutic literature, with supporting* in vitro* evidence, include* Citrus limon* (lemon),* Lavandula latifolia *(lavender),* M. piperita*,* Santolina insularis *(santolina),* M. alternifolia*,* E. globulus*, and* S. officinalis*. Of these oils, the latter three are not ideally suited for antiviral use against HSV-1, due firstly to the IC_50_ values being weaker than what is recommended (less than 0.0010% or 1.00 *μ*g/mL) and due to their low selectivity index (below 4) [331, 332, 334, 340, 341]. Essential oils still to be studied according to the literature include* C. zeylanicum, C. bergamia, Pelargonium odoratissimum* (geranium), and* Tagetes minuta* (Mexican marigold).

In a small pilot study, consisting of 18 patients undergoing treatment of recurrent herpes labialis, a 6%* M. alternifolia* oil gel applied five times daily was compared to a placebo gel [349]. Reepithelialisation occurred after nine days for the test group compared to the placebo group where reepithelialisation occurred only after 12.5 days. Millar and Moore [350], undertook a case study of a patient with six reoccurring warts (human papillomavirus) after countless treatments with 12% w/w salicylic acid and lactic acid (4% w/w) for several weeks. Alternative treatment consisted of 100% topical* M. alternifolia *oil applied each evening straight after bathing and prior to bedtime. After five days, a significant reduction in wart size was observed, and, after an additional seven days, all warts were cleared, with complete reepithelialisation of the infected areas and no recurrence. The main shortfall of the two studies is the small sample size. It should also be recommended that any trial involving viral pathogens include a one-, two-, and six-month follow-up after the discontinuation of treatment, the reason being due to the tendency of viral pathogens remaining dormant for an extended period. It can then be observed how effective the essential oil is for long-term effects.

The nonenveloped (such as HPV) viruses have thus far been shown to be more resilient to essential oils [30] compared to the enveloped viruses (HSV) which are more susceptible to essential oils that could dissolve the lipid membrane [5]. Essential oil studies against viruses are clearly lacking. The most studied virus is HSV, which is one of the most prevalent viruses [351], and the most studied essential oil is* M. alternifolia*. Although numerous studies have proven efficacy of tea tree oil, the problem with a few of the studies is that these were compared to a placebo, which is expected to display poor activity.

Although these studies demonstrate some antiviral activity, other viral pathogens (e.g., varicella zoster, herpes zoster, human papillomavirus, and* Molluscum contagiosum*) associated with skin infections have clearly been neglected and warrant further study.

## 7. Essential Oil Toxicity

Plants used for therapeutic purposes are normally assumed to be safe and free of toxicity. This misconception is mainly due to the long-term usage of medicinal plants for the treatment of diseases based on basic knowledge accumulated and shared from generation to generation over many centuries. However, scientific studies and reports have highlighted the toxic effects of essential oils used to treat skin ailments, which are known to produce adverse effects such as allergic contact dermatitis, skin irritation, or photosensitization [300]. Phenols and aldehyde containing oils may often cause irritation [352]. Furanocoumarin containing essential oils (such as* C. bergamia*) have been proven to induce phototoxicity [353–355]. The evidence based review on botanicals in dermatology by Reuter et al. [18] identifies certain medicinal plants which have been used for dermatological purposes, which have also reported toxic effects. These include* C. bergamia* and* M. recutita*.* Mentha piperita* oil has been reported to cause dermal irritation [356]. Prashar et al. [357] have shown in an* in vitro* study that* L. angustifolia* oil and linalool (one of the main compounds) are cytotoxic to human fibroblast and endothelial cells [357]. There have also been a few case reports on* L. angustifolia* use resulting in contact dermatitis [358–360].

Stonehouse and Studdiford [361] determined that nearly 5% of patients that use* M. alternifolia* oil will experience allergic contact dermatitis. Centred on a patch test study of 311 volunteers, it was determined that neat 5% tea tree oil can cause irritancy (mean irritancy score of 0.25) [362]. In contrast, however, the study of 217 patients from a dermatology clinic, subjected to a patch test with 10%* M. alternifolia* oil, showed no irritation [363]. Two additional studies tested the* M. alternifolia* in patch tests at concentrations of 5% and 10%; 0.15–1.8% of patients experienced allergic contact dermatitis [364, 365]. However, considering that patch tests exaggerate real-world product use [366, 367], they do not necessarily give a good indication of products containing the essential oils. This is evident in the discussed clinical trials using* M. alternifolia* oil where only mild reactions were observed [189, 200, 219, 281, 282, 285]. Increasing the oil concentration to 25–100%, however, resulted in an increased risk of contact dermatitis in 2–8% of patients [275, 279]. Several additional reports exist reporting contact dermatitis and one systemic hypersensitivity reaction, from the use of* M. alternifolia *[368–371].

As the prospective use of these essential oils may be for topical application, it is necessary to test toxicity against skin fibroblasts and human skin cell lines F1-73 [115].* Backhousia citriodora *oil at a concentration of 1.00% showed low toxicity to human skin cells and skin fibroblasts [115], whereas neat* B. citriodora* oil and citral were shown to be toxic to human skin cells (F1-73) and skin fibroblasts [115].* Thymus quinquecostatus*,* w*hen tested against fibroblast cells for cytotoxicity, showed low cytotoxicity at concentrations below 12.5 *μ*g/mL in fibroblast cells and thus may be suitable for topical treatment [207].* Mentha piperita* is one of the most popularly used essential oils [372]; however, there have been reports that* M. piperita* oil can cause both dermal irritations [356]. A review by Reichling et al., containing more information regarding essential oil toxicity, is available [30].

## 8. Conclusion

Of all the skin pathogens studied, dermatophytes were found to be the most sensitive to essential oil inhibition, followed by the yeast* C. albicans* and then Gram-positive bacteria (anaerobes more than aerobes), with Gram-negative bacteria being the most resistant, especially* P. aeruginosa *[168, 181]. The most frequently studied organisms are* E. coli, P. aeruginosa*,* C. albicans*, and* S. aureus*. However, less attention has been paid to pathogens such as* S. epidermidis*,* H. influenzae*,* S. pyogenes*,* P. acnes*,* Clostridium* spp.,* Brevibacterium* spp., and the dermatophytes. The reason for this may be due to the difficulty in performing such studies on fastidious pathogens and the lack of a perceived threat. Furthermore, many of these pathogens are slow growing and, combined with the volatile nature of oils, may prove difficult in retaining the oil with the pathogen during the incubation period. Where possible, resistant strains should be included in essential oil studies, along with the reference strain [56, 147]. Antiviral studies should extend to the neglected viruses. These should also report on which part of the cycle the inhibition occurred. The focus should be directed towards the aromatherapeutic recommendation of the essential oil and the responsible pathogens connected to the type of infection, together with the inclusion of the microorganism strain number, the solvent, essential oil composition, and the reason for testing. This is especially relevant for combination studies where it is ill advised to just randomly test different combinations.

Regardless of the frequency of the therapeutic claims made for essential oils and the proven* in vitro* activity, most evidence of the therapeutic efficacy of aromatherapy has been published in books about aromatherapy and not in peer-reviewed journals. A few clinical trials have emerged, but their results are rarely confirmed completely to substantiate essential oil effectiveness. More rigorous clinical trials would establish confidence from the medical professionals [352].

Besides the antimicrobial activities, toxicity studies are also recommended using skin fibroblasts for sensitivity, as the use is topical. The toxicological effects of essential oils are important facets that need to be addressed. Discernment also needs to be applied as certain sensitivity studies may have been done on rabbit skin; however, human skin has been found to be more sensitive to irritants [115].

Further essential oil combinations need to be studied, along with the reason for the combination selection. Whether the interaction is synergistic, additive, indifferent, or antagonistic, each interaction is a valuable result. If antagonism is not reported, it will not be known to avoid those combinations, which in turn will result in their continuous use, which may eventually lead to resistance to the essential oils themselves. Including synergistic results will allow for these essential oil combinations to be used more frequently in practice. The inclusion of additive and indifferent interactions is also vital in order to report essential oil combinations already studied. This will prevent unnecessary duplication of combination research and confirm essential oil combinations that have useful antimicrobial activity. This research will provide an insight into the understanding of these combinations which could allow for newer directives for integrating essential oils into mainstream medicine. Although essential oil combinations with other essential oils and with antimicrobials have started gaining some attention, there is still a gap in the research with regard to carrier oils. Essential oils are seldom used directly on the skin because direct use onto the skin can cause irritation [26, 38]. Therefore, essential oils are blended with carrier oils before they are applied to the skin. This raises the question as to whether or not the carrier oils influence the overall antimicrobial activity of the essential oils. Gemeda et al. [373] tested the antimicrobial activity of essential oils mixed in different hydrophilic and lipophilic bases. They found better effects in hydrophilic bases than in lipophilic bases. This study confirmed that the base may have an influence on the antimicrobial activity; however, carrier oils in combination have to the best of our knowledge not been studied further.

Essential oils, such as* M. alternifolia,* are often used in subinhibitory concentrations in commercial products such as shampoos, shower gels, and creams to enhance commercial selling point of a greener product or improve fragrance or desire for the product [202]. This in itself can cause resistance. Therefore, although essential oils are showing promise, the use of essential oils in subinhibitory concentrations in cosmetics and other dermatological formulations may weaken the efficacies of the essential oils as antiseptics, as was shown by Nelson [136]. This highlights the need to insure that there is sufficient evidence supporting aromatherapeutic combinations not only for therapeutics, but also in commercial products.

Resistant strains such as* P. aeruginosa*, MRSA, and methicillin-resistant* S. epidermidis *(MRSE) have become extensively problematic microorganisms in the recent years due to their antimicrobial resistance [158], and, as such, including these organisms in screening studies is becoming more and more important.

For viral studies, one needs to consider that genuine antiviral potential is seen for those essential oils that display activity after absorption into the host cell's nucleus because this is where viral DNA replicates by using viral DNA polymerase [30].

Clinical trial and ex vivo studies should consider regular essential oil dosing, instead of once daily, or every several days, application. According to the aromatherapeutic literature, essential oils are generally applied two to three times a day. The reason may be due to the volatile nature resulting in essential oil evaporation. Thus, in order to give credit to essential oil use, application studies should consider timed dosages.

Finally,* M. alternifolia* is the most studied of all commercial essential oils. However, many other oils have shown better antimicrobial activity. It is time essential oil researchers give just as much attention to oils such as* C. zeylanicum*,* L. scoparium*,* O. vulgare*,* S. album*, and* S. aromaticum* in the hope of increasing the global knowledge of essential oils used on the skin.

## Figures and Tables

**Table 1:** Essential oils used in dermatology.

Scientific name	Common name	Dermatological use	Reference
*Abies balsamea*	Balsam (Peru, Canadian)	*Burns* ^*∗*^, cracks, *cuts*, eczema, *rashes*, *sores*, and *wounds*	[32]

*Abies balsamea*	Fir	Skin tonic	[36]

*Acacia dealbata*	Mimosa	*Antiseptic*, general care, oily conditions, and nourisher	[2, 32]

*Acacia farnesiana*	Cassie	Dry or sensitive conditions	[32]

*Achillea millefolium*	Yarrow	*Acne*, *burns*, chapped skin, *cuts*, dermatitis, eczema, healing agent, *infections*, inflammation, oily conditions, pruritus, *rashes*, scars, toner, sores, *ulcers*, and *wounds*	[32, 36, 40, 42]

*Allium sativum*	Garlic	*Acne*, *antiseptic*, *fungal infections (ringworm)*, lupus, *septic wounds*, and *ulcers*	[32, 36]

*Amyris balsamifera*	Amyris	Inflammation	[36]

*Anethum graveolens*	Dill	Wound healing encouragement	[36]

*Angelica archangelica*	Angelica	Congested and dull conditions, *fungal infections*, inflammation, psoriasis, and tonic	[32, 36]

*Aniba rosaeodora*	Rosewood	*Acne*, congested conditions, *cuts*, damaged skin, dermatitis, general care, greasy and oily conditions, inflammation, psoriasis, scars, regeneration, *sores*, *wounds*, and wrinkles	[2, 32, 36, 37, 39, 41, 42]

*Anthemis nobilis*	Roman chamomile	*Abscesses*, *acne*, allergies, *antiseptic*, *blisters*, *boils*, *burns*, cleanser, *cuts*, dermatitis, eczema, *foot blisters*, general care, herpes, inflammation, insect bites and stings, *nappy rash*, nourisher, problematic skin, pruritus, psoriasis, rashes, *rosacea*, *sores*, sunburn, *ulcers*, and *wounds*	[2, 26, 32, 36–43]

*Apium graveolens*	Celery	Reducing puffiness and redness	[36]

*Artemisia dracunculus*	Tarragon	*Infectious wounds*	[36]

*Betula alba*	Birch (white)	Congested conditions, dermatitis, eczema, psoriasis, and *ulcers*	[32, 36]

*Boswellia carteri*	Frankincense/olibanum	*Abscesses*, *acne*, aged or dry and damaged complexions, *antiseptic*, *bacterial infections*, blemishes, *carbuncles*, dermatitis, *disinfectant*, eczema, *fungal and nail infections*, general care, healing agent, inflammation, oily conditions, psoriasis, problematic conditions, regeneration or rejuvenation, scars, *sores*, toner, tonic, *ulcers*, *wounds*, and wrinkles	[1, 2, 32, 36–43]

*Bursera glabrifolia*	Linaloe (copal)	Acne, conditioning, cuts, dermatitis, sores, and wounds	[32, 40]

*Calendula officinalis*	Marigold	Athlete's foot, burns, cuts, diaper rash, eczema, fungal infections, inflammation, oily and greasy conditions, and wounds	[26, 32, 39]

*Cananga odorata*	Ylang-ylang	*Acne*, balancing sebum, dermatitis, eczema, general care, greasy and oily conditions, insect bites, and toner	[2, 32, 36–38, 40, 42, 43]

*Canarium luzonicum*	Elemi	Aged and dry complexions, *bacterial infections*, balancing sebum, *cuts*, *fungal infections*, inflammation, *sores*, *ulcers*, *wounds*, and wrinkles	[32, 36, 40]

*Carum carvi*	Caraway	*Acne*, *boils*, *infected wounds*, oily conditions, and pruritus	[36]

*Cedrus atlantica*	Cedar wood	*Acne*, *antiseptic*, ^*∗*^*bromodosis*, cellulite, cracked skin, dandruff, dermatitis, eczema, eruptions, *fungal infections*, general care, *genital infections*, greasy and oily conditions, inflammation, insect bites and stings, psoriasis, scabs, and *ulcers*	[1, 2, 32, 36–39, 41–43]

*Cinnamomum camphora*	Camphor (white)	*Acne*, burns, inflammation, oily conditions, spots, and *ulcers*	[32, 36, 42]

*Cinnamomum zeylanicum*	Cinnamon	*Antiseptic*, gum and tooth care, *warts*, and wasp stings	[32, 36, 37, 41, 42]

*Cistus ladanifer*	Rock rose/*Cistus*/labdanum	Aged complexion, *bacterial infections*, *bedsores*, blocked pores, eczema, oily conditions, *sores*, *ulcers*, *varicose ulcers*, *wounds*, and wrinkles	[2, 32, 40]

*Citrus aurantifolia*	Lime	*Acne*, *bacterial infections*, *boils*, cellulite, congested or greasy and oily conditions, *cuts*, insect bites, pruritus, tonic, *sores*, *ulcers*, *warts*, and *wounds*	[2, 32, 36, 40–43]

*Citrus aurantium *var.* amara*	Neroli	*Acne*, aged and dry complexions, *antiseptic*, broken capillaries, *cuts*, dermatitis, eczema, general care, healing agent, psoriasis, scars, stretch marks, toner, tonic, thread veins, *wounds*, and wrinkles	[2, 26, 32, 36–43]

*Citrus aurantium *var.* amara*	Petitgrain	*Acne*, *antiseptic*, *bacterial infections*, balancing sebum, blemishes, greasy and oily conditions, ^∗∗^ hyperhidrosis, *pimples, pressure sores*, sensitive complexions, toner, tonic, and *wounds*	[1, 2, 32, 36, 37, 39–42]

*Citrus bergamia*	Bergamot	*Abscesses*, *acne*, *antiseptic*, *athlete's foot*, *bacterial infections*, *blisters*, *boils*, *cold sores*, deodorant, dermatitis, eczema, *fungal infections*, greasy and oily conditions, healing agent, inflammation, insect bites, pruritus, psoriasis, shingles, ulcers, *viral infections (chicken pox*,* herpes, *and* shingles)*, and *wounds*	[2, 26, 32, 36, 37, 40–43]

*Citrus limon*	Lemon	*Abscesses*, *acne*, *antiseptic*, *athlete's foot*, *blisters*, *boils*, cellulite, corns, *cuts*, *grazes*, greasy and oily conditions, insect bites, mouth ulcers, *rosacea*, *sores*,*ulcers*, *viral infections (cold sores*,* herpes*,* verrucae*,and* warts)*, and *wounds*	[1, 2, 26, 32, 36, 37, 39, 41–43]

*Citrus paradisi*	Grapefruit	*Acne*, *antiseptic*, cellulite improvement, cleanser, combination and problematic skin, congested and oily conditions, stretch marks, and toner	[1, 2, 32, 36, 37, 39–43]

*Citrus reticulata*	Mandarin	*Acne*, cellulite, congested and oily conditions, general care, healing agent, scars, stretch marks, and toner	[1, 32, 36–38, 40, 43]

*Citrus sinensis*	Orange	*Acne*, blocked pores, congested and oily conditions, dermatitis, dry and dull complexions, problematic skin, *ulcers*, and wrinkles	[1, 32, 36–38, 40–43]

*Citrus tangerina*	Tangerine	*Acne*, chapped skin, inflammation, oily conditions, *rashes*, stretch marks, and toner	[36, 40, 42]

*Commiphora myrrha*	Myrrh	*Acne*, *antiseptic*, *athlete's foot*, *bacterial infections*, *bedsores*, *boils*, cracked skin, *cuts*, dermatitis, eczema, *fungal infections (athlete's foot*,* ringworm)*, healing agent, inflammation, scars, *sores*, *ulcers*, *weeping wounds*, and wrinkles	[1, 2, 26, 32, 36–43]

*Coriandrum sativum*	Coriander	Used to prevent the growth of *odour causing bacteria*	[37]

*Cupressus sempervirens*	Cypress	*Acne*, blocked pores, *bromodosis*, cellulite, *cellulitis*, deodorant, hyperhidrosis, oily conditions, *rashes*, *rosacea*, and *wounds*	[1, 2, 32, 36–38, 40–43]

*Curcuma longa*	Turmeric	*Cuts*, *sores*, and *wounds*	[40]

*Cymbopogon citratus*	Lemongrass	*Acne*, *athlete's foot*, *bacterial infections*, blocked or open pores, cellulite, *fungal infections*, hyperhidrosis, oily conditions, and toner	[2, 32, 36, 37, 41, 42]

*Cymbopogon martinii*	Palmarosa	*Acne*, *bacterial infections*, balancing sebum, damaged and dry complexions, dermatitis, eczema, *fungal infections*, oily conditions, *pressure sores*, psoriasis, scars, toner, tonic, *sores*, *wounds*, and wrinkles	[2, 32, 36–42]

*Cymbopogon nardus*	Citronella	*Bromodosis*, hyperhidrosis, oily conditions, and softener	[32, 36, 42]

*Daucus carota*	Carrot seed	Aged and dry complexions, *carbuncles*, dermatitis, eczema, inflammation, oily conditions, pruritus, psoriasis, *rashes*, scarring, toner, *ulcers*, vitiligo, *weeping sores*, *wounds*, and wrinkles	[2, 32, 36, 40, 42]

*Dryobalanops aromatica*	Borneol (Borneo Camphor)	*Cuts* and *sores*	[32]

*Eucalyptus globulus*	Eucalyptus	*Abscesses*,* antiseptic, athlete's foot, bacterial dermatitis*,* bacterial infections*, blisters, *boils*, *burns*, *chicken pox*, cleanser, congested conditions, *cuts*, *fungal infections*, *general infections*, *herpes (cold sores)*, inflammation, insect bites, *shingles*, *sores*, *ulcers*, and *wounds*	[1, 26, 32, 36–39, 41–43]

*Syzygium aromaticum*	Clove	*Acne*,* antiseptic*,* athlete's foot*,* burns*,* cuts*,* cold sores*,* fungal infections,* lupus, *sores*, *septic ulcers*, and *wounds*	[32, 36, 37, 41, 42]

*Ferula galbaniflua*	Galbanum	*Abscesses*,* acne*,* blisters*,* boils*,* cuts*, inflammation, scar tissue improvement, toner, and *wounds*	[32, 36]

*Foeniculum dulce*	Fennel	Aged and wrinkled complexions, *bromodosis*, cellulite, *cellulitis*, congested, greasy, and oily conditions, cleanser, and tonic	[1, 32, 36, 37, 40–43]

*Guaiacum officinale*	Guaiacwood	Firming or tightening the skin	[36]

*Helichrysum italicum*	Immortelle/everlasting/*Helichrysum*	*Abscesses*,* acne*,* athlete's foot*,* bacterial infections*,* boils*,* blisters*, cell regeneration, *cuts*, damaged skin conditions, dermatitis, eczema, *fungal infections (ringworm)*, inflammation, psoriasis, *rosacea*, scars, *sores*, *ulcers*, and *wounds*	[2, 32, 36, 40, 41]

*Humulus lupulus*	Hops	Dermatitis, *ulcers*, *rashes*, and nourisher	[32]

*Hyssopus officinalis*	Hyssop	*Cuts*, dermatitis, eczema, healing agent, inflammation, scars, *sores*, and *wounds*	[32, 36, 41]

*Jasminum officinale*	Jasmine	Aged and dry complexions, general care, inflammation, revitalization, oily conditions, and psoriasis	[2, 26, 32, 36, 37, 40]

*Juniperus virginiana*	Juniper	*Acne*, *antiseptic*, blocked pores, cellulite, congested and oily conditions, deodorant, eczema, dermatitis, general care, *general infections*, psoriasis, toner, *ulcers*, weeping eczema, and *wounds*	[1, 2, 32, 36, 37, 39, 41–43]

*Juniperus oxycedrus*	Cade	*Cuts*, dermatitis, eczema, *sores*, and spots	[32]

*Kunzea ericoides*	Kānuka	*Athlete's foot*	[40]

*Laurus nobilis*	Bay	*Acne*, *fungal infections*, inflammation, oily conditions, *pressure sores*, and *varicose ulcers*	[32, 36, 41]

*Lavandula angustifolia*	Lavender	*Abscesses*,* acne*,* antiseptic*,* bacterial infections*,* blisters*,* boils*,* burns*,* carbuncles*, cellulite, congested and oily conditions, *cuts*, deodorant, dermatitis, eczema, *foot blisters*, *fungal infections (athlete's foot*,* ringworm)*, general care, healing agent, inflammation, insect bites and stings, *pressure sores*, pruritus, psoriasis, *rosacea*, scalds, scarring, *sores*, sunburn, *ulcers*,*viral infections (chicken pox*,* cold sores*,* shingles*,and* warts)*, and *wounds*	[2, 26, 32, 36–43]

*Lavandula flagrans*	Lavandin	*Acne*, *abscesses*, *boils*, blisters, congested conditions, *cuts*, eczema, healing agent, inflammation, insect bites and stings, *pressure sores*, scalds, *sores*, and *wounds*	[32, 36, 41]

*Lavandula spica*	Lavender spike	*Abscesses*, *acne*, *bacterial infections*, *blisters*, *boils*, *burns*, congested and oily conditions, *cuts*, dermatitis, eczema, inflammation, *fungal infections (athlete's foot, ringworm)*, *pressure sores*, psoriasis, *sores*, *ulcers*, and *wounds*	[32, 36, 41]

*Leptospermum scoparium*	Manuka	*Acne, cuts, fungal infections (athlete's foot*,* ringworm)*,* ulcers*, and *wounds*	[2, 40]

*Verbena officinalis*	Verbena	Congested conditions and nourisher	[36]

*Liquidambar orientalis*	Sweetgum	*Cuts*, *ringworm*, *sores*, and *wounds*	[32]

*Litsea cubeba*	May Chang	*Acne*, dermatitis, greasy and oily conditions, and hyperhidrosis	[32, 36]

*Melaleuca alternifolia*	Tea tree	*Abrasions*,* abscesses*,* acne, antiseptic*,* bacterial infections*, blemishes, *blisters*,* boils*,* burns*,* carbuncles*,* cuts*, dandruff, *fungal infections (athlete's foot, nails*,* ringworm, *and* tinea)*, inflammation, insect bites, oily conditions, *rashes*, *sores*, spots, sunburn, *ulcers*, *viral infections (cold sores*,* chicken pox*,* herpes*,* shingles*,and* warts)*, and *wounds*	[1, 2, 26, 32, 36–43]

*Melaleuca cajuputi *	Cajuput	*Acne*, insect bites, oily conditions, psoriasis, and spots	[32, 36, 42]

*Melaleuca viridiflora*	Niaouli/Gomenol	*Abscesses*,* acne, antiseptic, bacterial infections*,* blisters, boils*,* burns*,* chicken pox*, congested and oily conditions, *cuts*, eruptions, healing agent, insect bites, psoriasis, *sores*, *ulcers*, and *wounds*	[2, 32, 36, 39–42]

*Melissa officinalis*	Melissa/lemon balm	Allergic reactions, *cold sores*, eczema, *fungal infections*, inflammation, insect stings, *ulcers*, and *wounds*	[1, 26, 32, 36, 41, 42]

*Mentha piperita*	Peppermint	*Acne*, *antiseptic*, *blackheads*, *chicken pox*, congested and greasy conditions, dermatitis, inflammation, pruritus, *ringworm*, scabies, softener, toner, and sunburn	[1, 2, 32, 36, 37, 41–43]

*Mentha spicata*	Spearmint	*Acne*, congested conditions, dermatitis, pruritus, scabs, and *sores*	[32, 36, 39, 42]

*Myristica fragrans*	Nutmeg	Hair conditioner	[36]

*Myrocarpus fastigiatus*	Cabreuva	*Cuts*, *scars*, and *wounds*	[32]

*Myrtus communis*	Myrtle	*Acne*, *antiseptic*, blemishes, blocked pores, bruises, congested and oily conditions, and psoriasis	[2, 32, 36, 40]

*Nardostachys jatamansi*	Spikenard	Eczema, inflammation, psoriasis, and *sores*	[32, 40]

*Ocimum basilicum*	Basil	*Acne*, *antiseptic*, congested conditions, insect bites, and wasp stings	[1, 36, 37, 39, 40, 42]

*Origanum majorana*	Marjoram	Bruises and *fungal infections*	[32, 36]

*Origanum vulgare*	Oregano	*Athlete's foot*,* bacterial infections*,* cuts*, eczema, *fungal infections*, psoriasis, *warts*, and *wounds*	[36, 41]

*Pelargonium odoratissimum*	Geranium	*Acne*, aged and dry complexions, *bacterial infections, *balancing sebum, *burns*, cellulite, *chicken pox*, congested and oily conditions, cracked skin, *cuts*, dermatitis, deodorant, eczema, *fungal infections (athlete's foot*,* ringworm)*, general care, healing agent, *herpes*, *impetigo*, inflammation, *measles*, psoriasis, *rosacea*, *shingles*, problematic skin, *sores*, *ulcers*, and *wounds*	[2, 26, 32, 36–43]

*Pelargonium roseum*	Rose geranium	Aging and dry or wrinkled skin	[40]

*Petroselinum sativum*	Parsley	Bruises, scalp conditioning, and *wounds*	[36]

*Pimpinella anisum*	Anise	*Infectious diseases*	[36]

*Pinus sylvestris*	Pine	*Antiseptic*, *bromodosis*, congested conditions, *cuts*, eczema, hyperhidrosis, pruritus, psoriasis, and *sores*	[32, 36, 37, 41–43]

*Piper nigrum*	Black pepper	Bruises and *fungal infections*	[36, 42]

*Pistacia lentiscus*	Mastic	*Abscesses*, *blisters*, *boils*, *cuts*, *ringworm*, and *wounds*	[32]

*Pistacia palaestina*	Terebinth	*Abscesses*,* blisters, boils*,* cuts*,* infectious wounds*,* ringworm*, and *sores*	[32, 36]

*Pogostemon patchouli*	Patchouli	*Abscesses*, *acne*, chapped or damaged and cracked skin, dermatitis, *cold sores*, eczema, *fungal infections (athlete's foot)*, general care, healing agent, *impetigo*, inflammation, oily conditions, pruritus, scalp disorders, scars, *sores*, tonic, stretch marks, and *wounds*	[1, 2, 32, 36–43]

*Rosa damascena*	Rose otto	Aging and dry conditions, *bacterial infections*, eczema, inflammation, toner, tonic, and *wounds*	[2, 38–41]

*Rosa gallica*	Rose	Broken capillaries, *cuts*, dry and aging conditions, *burns*, eczema, healing agent, inflammation, pruritus, psoriasis, scars, toner, tonic, stretch marks, sunburn, thread veins, and wrinkles	[26, 32, 36–38, 42, 43]

*Rosmarinus officinalis*	Rosemary	*Acne*, *bacterial infections*, balancing sebum, cellulite, congested and oily conditions, dandruff, dermatitis, dry scalp, eczema, general care, and *rosacea*	[1, 32, 36, 37, 39, 41, 42]

*Salvia lavandulifolia*	Spanish sage	*Acne*,* antiseptic*,* bacterial infections*, cellulite, *cold sores*, cuts, dermatitis, deodorant, hyperhidrosis, oily conditions, psoriasis, *sores*, and *ulcers*	[32, 36, 37, 41]

*Salvia sclarea*	Clary sage	*Abscesses*, *acne*, balancing sebum, *blisters*, *boils*, cell regeneration, dandruff, dermatitis, greasy and oily conditions, hyperhidrosis of the feet, inflammation, *ulcers*, and wrinkles	[1, 2, 32, 36, 40, 42]

*Santalum album*	Sandalwood	*Acne*, *antiseptic*, *bacterial infections*, *boils*, *burns*, chapped or damaged and dry conditions, eczema, *fungal infections*, general care, greasy and oily conditions, inflammation, pruritus, sunburn, and *wounds*	[1, 2, 26, 32, 36–39, 41–43]

*Santolina chamaecyparissus*	Santolina	Inflammation, pruritus, *ringworm*, scabs, *verrucae*, and *warts*	[36]

*Styrax benzoin *	Benzoin	Cracks, *cuts*, dermatitis, eczema, healing, inflammation, injured and irritated conditions, pruritus, *sores*, and *wounds*	[1, 2, 32, 36, 40, 42]

*Tagetes minuta*	Tagetes	*Bacterial infections*, *fungal infections*, inflammation, and *viral infections (verrucae and warts)*	[32, 36, 42]

*Thymus vulgaris*	Thyme	*Abscesses*,* acne*,* antiseptic*,* blisters*,* burns*,* carbuncles, cellulitis*,* cuts*, deodorant, dermatitis, eczema, *fungal infections*, oily conditions, *sores*, and *wounds*	[1, 32, 36, 37, 41, 42]

*Tilia europaea*	Linden Blossom	Blemishes, *burns*, freckles, softener, tonic, and wrinkles	[36]

*Vetiveria zizanioides *	Vetiver	*Acne*, *antiseptic*, balancing sebum, *cuts*, eczema, malnourished and aging skin, oily conditions, *weeping sores*, and *wounds*	[1, 2, 32, 36, 37, 41, 42]

*Viola odorata*	Violet	*Acne*, bruises, congested and oily conditions, eczema, inflammation, *infections*, *ulcers*, and *wounds*	[2, 32, 36, 40]

*Zingiber officinale*	Ginger	Bruises, *carbuncles*, and *sores*	[36]

^*∗*^Conditions involved in dermatological infections are shown in italics.

^*∗∗*^A medical condition that causes excessive sweating.

**Table 2:** Pathogens responsible for infectious skin diseases.

Skin disease	Anatomical structure affected by infection	Responsible pathogens	Reference
*Bacterial infections*			
Abscesses	Skin and subcutaneous tissue	*Staphylococcus aureus; *methicillin-resistant* S. aureus* (MRSA)	[101]
Acne	Sebum glands	*Propionibacterium acnes*;* S. epidermidis*	[8, 102]
Actinomycosis	Skin and subcutaneous tissue	*Actinomyces israelii*	[5]
Boils/carbuncles and furuncles	Hair follicles	*S. aureus*	[8]
Bromodosis (foot odour)	Epidermis/cutaneous	*Brevibacterium *spp.; *P. acnes*	[6, 103]
Cellulitis	Subcutaneous fat	*β*-Hemolyticstreptococci; *S. aureus; *MRSA	[7, 8, 101]
Ecthyma	Cutaneous	*S. aureus*;* Streptococcus pyogenes*	[7]
Erysipelas	Dermis, intradermal	*S. pyogenes*	[8]
Erythrasma	Epidermis	*Corynebacterium minutissimum*	[5]
Folliculitis	Hair follicles	*S. aureus*; MRSA	[8, 101]
Impetigo	Epidermis	*S. pyogenes*; *S. aureus*	[8, 104, 105]
Periorbital cellulitis	Subcutaneous fat	*Haemophilus influenzae*	[106]
Surgical wounds	Skin, fascia, and subcutaneous tissue	*Escherichia coli*; *Enterococcus* spp.; *Pseudomonas aeruginosa*;* S. aureus*	[8]

*Necrotizing infections*			
Necrotizing fasciitis	Skin, fascia, subcutaneous tissue, and muscle	*S. pyogenes*; anaerobic pathogens	[5, 8, 107]
Gas forming infections	Skin, subcutaneous tissue, and muscle	Gram-negative and various anaerobes	[5]
Gas gangrene	Skin, subcutaneous tissue, and muscle	*Clostridium* spp. (*C. perfringens, C. septicum, C. tertium, C. oedematiens*, and *C. histolyticum*)	[5, 8, 107]

*Fungal infections*			
Candidal infections (intertrigo, balanitis, nappy rash, angular cheilitis, and paronychia)	Superficial skin	*Candida albicans*	[7]
Eumycetoma	Subcutaneous infection	*Madurella mycetomatis*	[108]
Dermatophytosis (tinea pedis/athlete's foot, tinea cruris, tinea capitis, tinea corporis, tinea manuum, and tinea unguium/onychomycosis)	Keratin layer, epidermis	Dermatophytes (*Microsporum, Epidermophyton, *and* Trichophyton *spp.)	[8]
Seborrheic dermatitis	Subcutaneous infection	*Malasseziafurfur*	[109]
Tinea/pityriasis versicolor	Superficial skin	*M. furfur*	[7, 110]

*Viral infections*			
Herpes simplex	Mucocutaneous epidermidis	Herpes simplex virus (HSV) type 1, orofacial disease; HSV type 2, genital infection	[7]
Chicken pox	Mucocutaneous epidermidis	Varicella zoster	
Molluscum contagiosum	Prickle cells of epidermidis	Poxvirus	
Shingles	Mucocutaneous epidermidis	Herpes zoster	

Warts and verrucae	Epidermis	Human papillomavirus	[5, 7]

**Table 3:** Essential oil studies against *S. aureus*.

Essential oil^a^	Method^b^	Species strain^c^	Solvent^d^	Result^e^	Main components^f^	Reference
*Abies balsamea* (fir/balsam)	MIC	*S. aureus* (ATCC 6538)	Acetone	3.00 mg/mL	*β*-Pinene (31.00%), bornyl acetate (14.90%), *δ*-3-carene (14.20%)	[99]

*Abies holophylla* (Manchurian fir)	MIC	*S. aureus* (ATCC 25923)	5% DMSO	21.80 mg/mL	Bicyclo[2.2.1]heptan-2-ol (28.05%), *δ*-3-carene (13.85%), *α*-pinene (11.68%), camphene (10.41%)	[111]
		*S. aureus* (ATCC 6538)		>21.80 mg/mL		
*Abies koreana* (Korean fir)	MIC	*S. aureus* (ATCC 25923)	5% DMSO	21.80 mg/mL	Bornyl ester (41.79%), camphene (15.31%), *α*-pinene (11.19%)	
		*S. aureus* (ATCC 6538)		>21.80 mg/mL		

*Achillea millefolium* (yarrow)	MIC	*S. aureus* (ATCC 25923)	Tween 80	72.00 mg/mL	Eucalyptol (24.60%), camphor (16.70%), *α*-terpineol (10.20%)	[112]

*Achillea setacea* (bristly yarrow)	MIC	*S. aureus* (ATCC 25923)	Tween 80	4.50 mg/mL	Sabinene (10.80%), eucalyptol (18.50%)	[113]

*Angelica archangelica* (angelica), root	MIC	*S. aureus* (ATCC 6538)	Acetone	1.75 mg/mL	*α*-Phellandrene (18.50%), *α*-pinene (13.70%), *β*-phellandrene (12.60%), *δ*-3-carene (12.1%)	[99]
*Angelica archangelica* (angelica), seed				2.00 mg/mL	*β*-Phellandrene (59.20%)	

*Anthemis aciphylla *var.* discoidea* (chamomile), flowers	MIC	*S. aureus* (ATCC 6538)	DMSO	1.00 mg/mL	*α*-Pinene (39.00%), terpinen-4-ol (32.10%)	[114]
*Anthemis aciphylla *var.* discoidea* (chamomile), aerial parts				0.50 mg/mL	*α*-Pinene (49.40%), terpinen-4-ol (21.80%)	
*Anthemis aciphylla *var.* discoidea* (chamomile), leaves					Terpinen-4-ol (24.30%)	

*Anthemis nobilis* (chamomile)	MIC	*S. aureus* (ATCC 6538)	Acetone	16.00 mg/mL	2-Methylbutyl-2-methyl propanoic acid (31.50%), limonene (18.30%), 3-methylpentyl-2-butenoic acid (16.70%), isobutyl isobutyrate (10.00%)	[99]

*Artemisia dracunculus* (tarragon)	MIC	*S. aureus* (ATCC 6538)	Acetone	3.00 mg/mL	Estragole (82.60%)	[99]

*Backhousia citriodora* (lemon myrtle)	ADM	*S. aureus* (NCTC 4163)	Tween 20	0.05% v/v	Geranial (51.40%), neral (40.90%)	[115]
		MRSA (clinical isolate)		0.20% v/v		

*Boswellia carteri* (frankincense) (9 samples)	MIC	*S. aureus* (ATCC 12600)	Acetone	5.00–16.00 mg/mL	*α*-Pinene (4.80–40.40%), myrcene (1.60–52.40%), limonene (1.90–20.40%), *α*-thujene (0.30–52.40%), *p*-cymene (2.70–16.90%), *β*-pinene (0.30–13.10%)	[116]
*Boswellia frereana* (frankincense) (3 samples)				4.00–12.00 mg/mL	*α*-Pinene (2.00–64.70%), *α*-thujene (0.00–33.10%), *p*-cymene (5.40–16.90%)	
*Boswellia neglecta* (frankincense)				6.00 mg/mL	NCR	[117]
					*α*-Pinene (43.40%), *β*-pinene (13.10%)	[116]
*Boswellia papyrifera* (frankincense)				1.50 mg/mL	NCR	[117]
*Boswellia rivae* (frankincense)				2.50 mg/mL		
*Boswellia sacra* (frankincense) (2 samples)				4.00–8.00 mg/mL	*α*-Pinene (18.30–28.00%), *α*-thujene (3.90–11.20%), limonene (11.20–13.10%)	[116]
*Boswellia *spp. (frankincense) (4 samples)				6.00–9.30 mg/mL	*α*-Pinene (18.80–24.20%), limonene (11.70–19.00%)	
*Boswellia thurifera* (frankincense)				10.00 mg/mL	*α*-Pinene (28.0%), limonene (14.6%)	

*Cananga odorata* (ylang-ylang)	MIC	*S. aureus* (ATCC 6538)	Acetone	2.00 mg/mL	Bicyclosesquiphellandrene (19.50%), *β*-farnesene (13.90%)	[99]
*Cananga odorata* (ylang-ylang), heads				4.00 mg/mL	Benzyl acetate (31.90%), linalool (27.00%), methyl benzoate (10.40%)	

*Canarium luzonicum* (elemi)	MIC	*S. aureus* (ATCC 6538)	Acetone	3.00 mg/mL	Limonene (41.90%), elemol (21.60%), *α*-phellandrene (11.40%)	[99]

*Carum carvi* (caraway)	MIC	*S. aureus* (ATCC 6538)	Acetone	2.00 mg/mL	Limonene (27.60%), carvone (67.50%)	[99]
		*S. aureus*	DMSO	≤1.00 *μ*g/mL	DL-limonene (53.35%), *β*-selinene (11.08%), *β*-elemene (10.09%)	[118]

*Caryophyllus aromaticus* (clove)	ADM_90_	*S. aureus* (ATCC 25923, 16 MRSA and 15 MSSA clinical isolates)	Tween 80	2.70 mg/mL	Eugenol (75.85%), eugenol acetate (16.38%)	[119]

*Cinnamomum Cassia* (cinnamon)	MIC	*S. aureus*	DMSO	≤1.00 *μ*g/mL	*trans*-Caryophyllene (17.18%), eugenol (14.67%),	[118]
					Linalool L (14.52%), *trans*-cinnamyl acetate (13.85%), cymol (11.79%), cinnamaldehyde (11.25%)	

*Cinnamomum zeylanicum* (cinnamon)	MIC	*S. aureus* (ATCC 6538)	Acetone	2.00 mg/mL	Eugenol (80.00%)	[99]
	MIC	*S. aureus* (ATCC 25923)	n.m.	0.02 mg/mL	NCR	[85]
	ADM		10% DMSO	3.20 mg/mL		[80]
	ADM_90_	*S. aureus* (ATCC 25923, 16 MRSA and 15 MSSA clinical isolates)	Tween 80	0.25 mg/mL	Cinnamaldehyde (86.31%)	[119]

*Citrus aurantifolia* (lime)	ADM	*S. aureus* (ATCC 25923)	10% DMSO	12.80 mg/mL	Cinnamaldehyde (52.42%)	[80]

*Citrus aurantium* (bitter orange), flowers	MIC	*S. aureus* (ATCC 25923)	50% DMSO	0.31 mg/mL	Limonene (27.50%), *E*-nerolidol (17.50%), *α*-terpineol (14.00%)	[120]
	MIC	*S. aureus* (ATCC 6536)		0.63 mg/mL		

*Citrus aurantium* (petitgrain)	MIC	*S. aureus* (ATCC 6536)	Acetone	4.00 mg/mL	Linalyl acetate (54.90%), linalool (21.10%)	[99]

*Citrus bergamia* (bergamot)	MAC	*S. aureus* (ATCC 6538)	n.m.	1.25 *μ*L/mL	Bergamol (16.10%), linalool (14.02%), D-limonene (13.76%)	[62]

*Citrus grandis* (grapefruit)	MIC	*S. aureus* (ATCC 6538)	Acetone	3.00 mg/mL	Limonene (74.80%)	[99]

*Citrus medica limonum* (lemon)	ADM	*S. aureus *(ATCC 25923)	10% DMSO	>12.80 mg/mL	NCR	[80]
	MIC	*S. aureus* (ATCC 6538)	Acetone	3.00 mg/mL		[99]

*Citrus sinensis* (orange)	ADM	*S. aureus* (ATCC 25923)	10% DMSO	>12.80 mg/mL	NCR	[80]
	MAC	*S. aureus* (ATCC 9144)	0.1% ethanol	0.94 mg/L		[121]
	MIC	*S. aureus* (ATCC 6538)	Acetone	4.00 mg/mL	Limonene (93.20%)	[99]

*Commiphora guidotti* (myrrh)	MIC	*S. aureus* (ATCC 12600)	Acetone	1.50 mg/mL	*(E)*-*β*-Ocimene (52.60%), *α*-santalene (11.10%), *(E)*-bisabolene (16.00%)	[117]

*Commiphora myrrha* (myrrh)	MIC	*S. aureus* (ATCC 12600)	Acetone	1.30 mg/mL	Furanogermacrene (15.90%), furanoeudesma-1,3-diene (44.30%)	[117]
		*S. aureus* (ATCC 6538)		2.00 mg/mL	Furanoeudesma-1,3-diene (57.70%), lindestrene (16.30%)	[117]

*Coriandrum sativum* (coriander), seed	MIC	*S. aureus* (7 clinical isolates)	0.5% DMSO with Tween 80	0.16 mg/mL	NCR	[122]

*Cupressus arizonica* (smooth cypress), branches	MIC	*S. aureus* (ATCC 25923)	10% DMSO	1.50 *μ*g/mL	*α*-Pinene (58.60%), *δ*-3-carene (15.60%)	[123]
*Cupressus arizonica* (smooth cypress),female cones				2.95 *μ*g/mL	*α*-Pinene (60.50%), *δ*-3-carene (15.30%)	
*Cupressus arizonica* (smooth cypress), leaves				0.98 *μ*g/mL	*α*-Pinene (20.00%), umbellulone (18.40%)	

*Cupressus sempervirens* (cypress)	MIC	*S. aureus* (ATCC 6538)	Acetone	12.00 mg/mL	*α*-Pinene (41.20%), *δ*-3-carene (23.70%)	[99]

*Cymbopogon giganteus* (lemongrass)	MIC	*S. aureus* (ATCC 9144)	0.5% ethanol	2.10 mg/mL	Limonene (42.00%), *trans*-*p*-mentha-1(7),8-dien-2-ol (14.20%), *cis*-*p*-mentha-1(7),8-dien-2-ol (12.00%)	[124]

*Cymbopogon citratus* (lemongrass)	MIC	*S. aureus* (ATCC 9144)	0.5% ethanol	2.50 mg/mL	Geranial (48.10%), neral (34.60%), myrcene (11.00%)	[124]
		*S. aureus*	DMSO	≤1.00 *μ*g/mL	Geranial (47.34%), *β*-myrcene (16.53%), *Z*-citral (8.36%)	[118]
	MAC	*S. aureus* (MTCC 96)	Sodium taurocholate	0.80–0.27 *μ*L/mL	Citral (72.80%)	[125, 126]
	MIC	*S. aureus* (ATCC 6538)	Acetone	1.67 mg/mL	Geranial (44.80%)	[99]

*Cymbopogon martinii* (palmarosa)	MAC	*S. aureus* (MTCC 96)	Sodium taurocholate	0.80 *μ*L/mL	Geraniol (61.6%)	[125, 126]

*Cymbopogon nardus* (citronella)	MIC	*S. aureus* (ATCC 6538)	Acetone	4.00 mg/mL	Citronellal (38.30%), geraniol (20.70%), citronellol (18.80%)	[99]

*Daucus carota* (carrot seed)	MIC	*S. aureus* (ATCC 6538)	Acetone	2.00 mg/mL	Carotol (44.40%)	[99]

*Eucalyptus camaldulensis* (eucalyptus)	MAC	*S. aureus* (ATCC 25923)	Acetone	3.90 *μ*g/mL	1,8-Cineol (54.37%), *α*-pinene (13.24%)	[127]
		*S. aureus* (clinical isolate)				

*Eucalyptus globulus* (eucalyptus)	MIC	*S. aureus* (ATCC 25923)	Tween 80	10.00 mg/mL	1,8-Cineol (81.93%)	[128]
		MRSA (ATCC 10442)				
		MRSA (MRSA USA 300)				
	MIC	*S. aureus* (ATCC 43387)	DMSO	0.20% v/v	NCR	[129]
	MAC	*S. aureus* (MTCC 96)	Sodium taurocholate	0.41 *μ*L/mL	Cineole (23.20%)	[125, 126]
	ADM	MRSA (ATCC 33592)	Tween 20	85.60 *μ*g/mL	Eucalyptol (47.20%), (+)-spathulenol (18.10%)	[81]
		*S. aureus* (ATCC 25922)		51.36 *μ*g/mL		
		MRSA (14 clinical isolates)		8.56–85.60 *μ*g/mL		
	MIC	*S. aureus* (ATCC 6538)	Acetone	4.00 mg/mL	1,8-Cineole (58.00%), *α*-terpineol (13.20%)	[99]
	MIC	*S. aureus* (ATCC 25923)	Acetone	2.00 mg/mL	NCR	[130]
		MRSA (ATCC 33592)		0.75 mg/mL		

*Eucalyptus radiata* (eucalyptus)	MIC	*S. aureus* (ATCC 25923)	Acetone	2.00 mg/mL	1,8-Cineole (65.7% ± 9.5), *α*-terpineol (12.8% ± 4.4)	[130]
		MRSA (ATCC 33592)		1.00–2.00 mg/mL		

*Eucalyptus camaldulensis *(eucalyptus)	MIC	*S. aureus* (ATCC 25923)	Acetone	0.50 mg/mL	NCR	[130]
		MRSA (ATCC 33592)				

*Eucalyptus citriodora* (eucalyptus)	MIC	*S. aureus* (ATCC 25923)	Acetone	1.00 mg/mL	NCR	[130]
		MRSA (ATCC 33592)				

*Eucalyptus smithii* (eucalyptus)	MIC	*S. aureus* (ATCC 25923)	Acetone	2.00 mg/mL	NCR	[130]
		MRSA (ATCC 33592)				

*Eucalyptus dives* (eucalyptus)	MIC	*S. aureus* (ATCC 25923)	Acetone	2.00 mg/mL	NCR	[130]
		MRSA (ATCC 33592)		1.00 mg/mL		

*Eucalyptus intertexta* (eucalyptus)	MIC	*S. aureus* (ATCC 29737)	10% DSMO	7.80 *μ*g/mL	NCR	[131]

*Eucalyptus largiflorens* (eucalyptus)	MAC	*S. aureus* (ATCC 25923)	n.m.	7.80 *μ*g/mL	1,8-Cineol (70.32%), *α*-pinene (15.46%)	[127]
		*S. aureus* (clinical isolate)				
	MIC	*S. aureus* (ATCC 29737)	10% DSMO	250.00 *μ*g/mL	NCR	[131]

*Eucalyptus melliodora* (eucalyptus)	MAC	*S. aureus* (ATCC 25923)	n.m.	3.90 *μ*g/mL	1,8-Cineol (67.65%), *α*-pinene (18.58%)	[127]
		*S. aureus* (clinical isolate)				

*Eucalyptus polycarpa* (eucalyptus)	MAC	*S. aureus* (ATCC 25923)	n.m.	1.95 *μ*g/mL	1,8-Cineol (50.12%)	[127]
		*S. aureus* (clinical isolate)		3.90 *μ*g/mL		

*Foeniculum dulce* (fennel)	MIC	*S. aureus* (ATCC 6538)	Acetone	2.00 mg/mL	*E*-Anethole (79.10%)	[99]

*Foeniculum vulgare* (fennel)	MAC	*S. aureus* (ATCC 25923)	DMSO	>10.00 mg/mL	*trans*-Anethole (68.53%), estragole (10.42%)	[132]
*Foeniculum vulgare* (fennel) (6 samples)	MIC	*S. aureus*		≤1.00 *μ*g/mL	*trans*-Anethole (33.3%), DL-limonene (19.66%), carvone (12.03%)	[118]
		*S. aureus* (ATCC 28213)		125.00–500.00 *μ*g/mL	Fenchone (16.90–34.70%), estragole (2.50–66.00%), *trans*-anethole (7.90–77.70%)	[133]

*Foeniculum vulgare* Mill. ssp. v*ulgare* (fennel), Aurelio	MAC	*S. aureus* (ATCC 25923)	Tween 20	50.00–100.00 *μ*g/mL	Limonene (16.50–21.50%), *(E)*-anethole (59.80–66.00%)	[134]
*Foeniculum vulgare* Mill. ssp. *vulgare* (fennel), Spartaco					Limonene (0.20–17.70%), *(E)*-anethole (66.30–90.40%)	

*Geranium dissectum* (geranium)	MIC	*S. aureus*	DMSO	≤1.00 *μ*g/mL	*β*-Citronellol (25.45%), geraniol (13.83%)	[118]

*Hyssopus officinalis* (hyssop)	MIC	*S. aureus* (ATCC 6538)	Acetone	3.00 mg/mL	Isopinocamphone (48.70%), pinocamphone (15.50%)	[99]

*Juniperi aetheroleum* (juniper)	MAC_80_	*S. aureus* (ATCC 6538)	n.m.	40.00% v/v	*α*-Pinene (29.17%), *β*-pinene (17.84%), sabinene (13.55%)	[135]
	MAC_80_	*S. aureus* (MFBF)		15.00% v/v		

*Juniperus communis* (juniper), berry	MIC	*S. aureus* (ATCC 25923)	n.m.	10.00 mg/mL	NCR	[85]
	MIC	MRSA (15 clinical isolates)	Ethanol	>2.00% v/v		[136]

*Juniperus excelsa* (juniper), berries, Dojran	ADM	*S. aureus* (ATCC 29213)	50% DSMO	>50.00%	*α*-Pinene (70.81%)	[87]
*Juniperus excelsa* (juniper), leaves, Dojran				125.00%	*α*-Pinene (33.83%)	
*Juniperus excelsa* (juniper), leaves, Ohrid				125.00%	Sabinene (29.49%)	

*Juniperus officinalis* (juniper), berry	MIC	*S. aureus* (ATCC 29213)	Tween 80	10.00 mg/mL	*α*-Pinene (39.76%)	[128]
*Juniperus officinalis* (juniper), berry		MRSA (clinical isolates)		20.00 mg/mL		

*Juniperus virginiana* (juniper)	MIC	*S. aureus* (ATCC 6538)	Acetone	2.00 mg/mL	Thujopsene (29.80%), cedrol (14.90%), *α*-cedrene (12.40%)	[99]
*Juniperus virginiana* (juniper), berries				3.00 mg/mL	*α*-Pinene (20.50%), myrcene (13.70%), bicyclosesquiphellandrene (10.70%)	

*Kunzea ericoides* (Kānuka)	MAC	*S. aureus* (ATCC 6538)	Tween 80	0.25% v/v	*α*-Pinene (61.60%)	[137]
		MRSA (clinical isolate)		0.20% v/v		
	MIC	*S. aureus* (ATCC 12600)	Acetone	8.00 mg/mL	*α*-Pinene (26.2–46.7%), p-cymene (5.8–19.1%)	[138]

*Laurus nobilis* (bay)	MIC	*S. aureus* (ATCC 6538)	Acetone	0.83 mg/mL	Eugenol (57.20%), myrcene (14.30%), carvacrol (12.70%)	[99]

*Lavandula angustifolia* (lavender)	MIC	*S. aureus* (ATCC 6538)	Acetone	2.00 mg/mL	Linalyl acetate (36.70%), linalool (31.40%), terpinen-4-ol (14.90%)	[99]
		*S. aureus* (NCTC 6571)	10% DSMO	310.00 *μ*g/mL	Linalool (25.10%), linalyl acetate (22.50%)	[139]
		*S. aureus* (NCTC 1803)		320.40 *μ*g/mL		
		MRSA (15 clinical isolates)	Ethanol	0.50% v/v	NCR	[136]
		*S. aureus* (ATCC 12600)	Acetone	8.60 mg/mL	Linalool (30.80%), linalyl acetate (31.30%)	[140]
		*S. aureus* (clinical strain and ATCC 6538)	Acetone	2.00 mg/mL	Linalyl acetate (36.7%), linalool (31.4%), terpinen-4-ol (14.9%)	[99]
		MRSA (clinical strain and 43300)				
		Methicillin-gentamicin-resistant *S. aureus* (MGRSA) (ATCC 33592)				

*Lavandula dentata* (French lavender)	MIC	*S. aureus* (BNI 18)	5% DMSO	1.53 mg/mL	Camphor (12.40%)	[141]
*Lavandula officinalis* (lavender)	MIC	*S. aureus*	DMSO	≤1.00 *μ*g/mL	*δ*-3-Carene (17.14%), *α*-fenchene (16.79%), diethyl phthalate (13.84%)	[118]
*Lavandula stoechas* (French lavender)	MIC	*S. aureus* (STCC 976)	Tween 80	2.00 *μ*L/mL	10s,11s-Himachala-3(12),4-diene (23.62%), cubenol (16.19%)	[142]

*Lavandula stoechas* (French lavender), flower	MIC	MRSA (clinical isolate)	20% DMSO	31.25 *μ*g/mL	*α*-Fenchone (39.20%)	[47]
*Lavandula stoechas* (French lavender), leaf				125.00 *μ*g/mL	*α*-Fenchone (41.90%), 1,8-cineole (15.60%), camphor (12.10%)	

*Leptospermum scoparium* (manuka)	MAC	*S. aureus* (ATCC 6538)	Tween 80	0.10% v/v	(−)-(*E*)-Calamenene (14.50%), leptospermone (17.60%)	[137]
		MRSA (clinical isolate)		0.05% v/v		
	MIC	*S. aureus* (ATCC 12600)	Acetone	4.00 mg/mL	Eudesma-4(14),11-diene (6.2–14.5%), *α*-selinene (5.90–13.5%), (*E*)-methyl cinnamate (9.2–19.5%)	[138]

*Litsea cubeba* (May Chang)	MIC	*S. aureus* (ATCC 6538)	Acetone	1.50 mg/mL	Geranial (45.60%), nerol (31.20%)	[99]

*Matricaria chamomilla* (German chamomile)	MIC	*S. aureus* (ATCC 6538)	Acetone	1.50 mg/mL	Bisabolene oxide A (46.90%), *β*-farnesene (19.20%)	[99]

*Matricaria recutita* (German chamomile)	ADM_90_	*S. aureus* (ATCC 25923, 16 MRSA and 15 MSSA clinical isolates)	Tween 80	26.50 mg/mL	Chamazulene (31.48%), *α*-bisabolol (15.71%), bisabolol oxide (15.71%)	[119]

*Matricaria songarica* (chamomile)	MIC	*S. aureus* (CCTCC AB91093)	Tween 80	50.00 *μ*g/mL	*E*-*β*-Farnesene (10.58%), bisabolol oxide A (10.46%)	[143]

*Melaleuca alternifolia* (tea tree)	ADM	*S. aureus* (NCIM 2079)	Tween 80	1.00%	NCR	[79]
		*S. aureus* (clinical isolate)				
	MAC	*S. aureus* (ATCC 6538)	Tween 80	0.25% v/v	*α*-Terpinene (11.40%), *γ*-terpinene (22.50%), terpinen-4-ol (35.20%)	[137]
		MRSA (clinical isolate)		0.35% v/v		
	MIC	*S. aureus* (ATCC 29213)	None used	0.50% (v/v)	Terpinen-4-ol (40.00%), *δ*-terpinen (13.00%), *p*-cymene (13.00%)	[97]
		MRSA (98 clinical isolates)	n.m.	512.00–2048.00 mg/L	NCR	[144]
		*S. aureus* (NCIB 6571)		1.00% v/v		[145]
		Coagulase-negative staphylococci (9 clinical isolates)	Polyoxyl 35 castor oil	0.63–2.50% v/v	Terpinen-4-ol (>35.00%)	[146]
		MRSA (10 clinical isolates)		0.30–0.63% v/v		
				0.30% v/v		
		MRSA (15 clinical isolates)	Ethanol	0.25% v/v	NCR	[136]
		*S. aureus* (ATCC 12600)	Acetone	8.60 mg/mL	Terpinen-4-ol (38.60%), *γ*-terpinene (21.60%)	[140]
	MIC_90_	*S. aureus* (NCTC 6571)	Tween 80	0.25% v/v	Terpinen-4-ol (35.70%)	[147]
		*S. aureus* (105 clinical isolates)		0.12–0.50% v/v		
	MIC	MRSA (60 clinical isolates, 29 mupirocin-resistant)		0.25%		[148]
	MIC	*S. aureus* (NCTC 8325)	n.m.	0.50% (v/v)	Terpinen-4-ol (39.80%), *γ*-terpinene (17.80%)	[149]
				0.25% (v/v)		[150]
	MIC_90_	MRSA (100 clinical isolates)	Tween 80	0.16–0.32%	NCR	[151]
	MIC	*S. aureus* (69 clinical isolates)	Tween 80	0.12–0.50% v/v	Terpinen-4-ol (35.70%)	[152]
	ADM	*S. aureus* (NCTC 4163)	Tween 20	0.20% v/v	Terpinen-4-ol (42.80%), *γ*-terpinene (18.20%)	[115]
		MRSA (clinical isolate)		0.30% v/v		
	MIC	*S. aureus* (ATCC 6538)	Acetone	8.00 mg/mL	Terpinen-4-ol (49.30%), *γ*-terpinene (16.90%)	[99]
	MAC	*S. aureus* (2 clinical isolates)	n.m.	0.10–0.20%	Eucalyptol (70.08%)	[153]
		*S. aureus* (ATCC 25923)		0.20%		
		*S. aureus* (NCTC 9518)		0.63–1.25% v/v	*α*-Pinene (11.95%), *α*-terpinene (14.63%), terpinen-4-ol (29.50%), *p*-cymene (17.74%)	[154]
					*α*-Pinene (24.87%), *α*-terpinene (12.47%), terpinen-4-ol (28.59%)	

*Melaleuca cajuputi* (cajuput)	MIC	*S. aureus* (ATCC 25923)	Tween 80	2.50 mg/mL	1,8-Cineol (67.60%)	[128]
		MRSA (ATCC 10442)		5.00 mg/mL		
		MRSA (clinical isolate)		2.50 mg/mL		
	MAC	*S. aureus* (ATCC 6538)		0.20% v/v	1,8-Cineole (55.50%)	[137]
		MRSA (clinical isolate)		0.30% v/v		

*Melaleuca quinquenervia* (niaouli)	MAC	*S. aureus* (ATCC 6538)	Tween 80	0.20% v/v	1,8-Cineole (61.20%)	[137]
		MRSA (clinical isolate)		0.30% v/v		

*Melaleuca viridiflora* (niaouli)	MIC	*S. aureus* (ATCC 6538)	Acetone	2.00 mg/mL	1,8-Cineole (45.90%), *α*-terpinene (21.00%)	[99]

*Melissa officinalis* (lemon balm)	MIC	*S. aureus* (NCTC 6571)	10% DSMO	300.60 *μ*g/mL	1,8-Cineol (27.40%), *α*-thujone (16.30%), *β*-thujone (11.20%), borneol (10.40%)	[139]
	MIC	*S. aureus* (NCTC 1803)		330.30 *μ*g/mL		

*Mentha piperita* (peppermint)	MIC	*S. aureus* (ATCC 12600)	Acetone	11.90 mg/mL	Menthone (18.20%), menthol (42.90%)	[140]
		*S. aureus* (ATCC 25923)	Tween 80	0.60 mg/mL	1,8-Cineol (12.06%), menthone (22.24%), menthol (47.29%)	[128]
		MRSA (ATCC 10442)				
		MRSA (clinical isolate)				
		*S. aureus* (ATCC 6538)	DMSO	0.63–2.50 mg/mL	Menthol (27.50–42.30%), menthone (18.40–27.90%)	[155]
		*S. aureus*		≤1.00 *μ*g/mL	Menthone (40.82%), carvone (24.16%)	[118]
		MRSA (15 clinical isolates)	Ethanol	0.50% v/v	NCR	[136]
		*S. aureus* (ATCC 43387)	DMSO	0.20% v/v		[129]
	MAC	*S. aureus* (MTCC 96)	Sodium taurocholate	1.66 *μ*L/mL	Menthol (36.40%)	[125, 126]
	MIC	*S. aureus* (ATCC 9144)	0.5% ethanol	8.30 mg/mL	Menthol (39.30%), menthone (25.20%)	[156]
	MIC	*S. aureus* (ATCC 6538)	Acetone	4.00 mg/mL	Menthol (47.50%), menthone (18.60%)	[99]

*Myrtus communis* (myrtle)	MIC	*S. aureus* (ATCC 6538)	Acetone	2.00 mg/mL	Myrtenyl acetate (28.20%), 1,8-cineole (25.60%), *α*-pinene (12.50%)	[99]
	ADM	*S. aureus* (ATCC 6538)	Tween 20	2.80 mg/mL	NCR	[157]
		*S. aureus* (ATCC 29213)				

*Ocimum basilicum* (basil)	MIC	*S. aureus* (ATCC 9144)	0.5% ethanol	2.50 mg/mL	Linalool (57.00%), eugenol (19.20%)	[156]
	MAC	*S. aureus* (ATCC 6538)	n.m.	1.25 *μ*L/mL	Eugenol (62.60%), caryophyllene (21.51%)	[62]
			Tween 80	0.07 × 10^−2^% v/v	Linalool (54.95%), methyl chavicol (11.98%)	[158]
		*S. aureus* (3 clinical strains)	Tween 80	((0.15–0.30) × 10)^−2^ % v/v	Linalool (54.95%), methyl chavicol (11.98%)	[158]
	MIC	*S. aureus* (ATCC 6538)	Acetone	1.50 mg/mL	Linalool (54.10%)	[99]
	MIC_90_	*S. aureus* (ATCC 6538)	n.m.	45.00 *μ*g/mL	Methyl chavicol (46.90%), geranial (19.10%), neral (15.15%)	[159]
	MIC	*S. aureus* (ATCC 6538)	Tween 80	0.68–11.74 *μ*g/mL	Linalool (30.30–58.60%)	[160]

*Origanum acutidens* (Turkey oregano)	MIC	*S. aureus* (clinical isolate)	10% DMSO	125.00 *μ*g/mL	Carvacrol (72.00%)	[161]
		*S. aureus* (ATCC2913)				

*Origanum majorana* (marjoram)	MIC	*S. aureus* (ATCC 43387)	DMSO	0.05% v/v	NCR	[129]
		*S. aureus* (ATCC 6538)	Acetone	2.00 mg/mL	1,8-Cineole (46.00%), linalool (26.10%)	[99]

*Origanum microphyllum* (oregano)	MIC	*S. aureus* (ATCC 25923)	Tween 80	6.21 mg/mL	Terpin-4-ol (24.86%), *γ*-terpinene (13.83%), linalool (10.81%)	[162]

*Origanum scabrum* (oregano)	MIC	*S. aureus* (ATCC 25923)	Tween 80	0.35 mg/mL	carvacrol (74.86%)	[162]

*Origanum vulgare* (oregano)	ADM	*S. aureus* (ATCC 6538)	1% DMSO	0.13% v/v	*p*-Cymene (14.60%), *γ*-terpinene (11.70%), thymol (24.70%), carvacrol (14.00%)	[163]
		*S. aureus* (ATCC 25923)				
		*S. aureus* (ATCC 43300)				
		MRSA (22 isolates)		0.06–0.13% v/v		
	MIC	*S. aureus* (ATCC 6538)	n.m.	575.00 mg/L	NCR	[164]
	MAC			0.63.00 *μ*L/mL	Carvacrol (30.17%), *p*-cymene (15.20%), *γ*-terpinen (12.44%)	[62]
	MIC	*S. aureus* (ATCC 43387)	DMSO	0.10% v/v	NCR	[129]
	ADM	*S. aureus* (ATCC 6538)	Tween 20	0.70 mg/mL		[157]
		*S. aureus* (ATCC 29213)				

*Origanum vulgare* subsp. *hirtum* (Greek oregano)	MIC	*S. aureus* (ATCC 25923)	10% DMSO + Tween 80	170.70 *μ*g/mL	Linalool (96.31%)	[165]
*Origanum vulgare* subsp. *vulgare* (oregano)				106.70 *μ*g/mL	Thymol (58.31%), carvacrol (16.11%), *p*-cymene (13.45%)	

*Pelargonium graveolens* (geranium)	ADM	*S. aureus* (ATCC 25923)	10% DMSO	>12.80 mg/mL	NCR	[80]
		*S. aureus* (ATCC 6538)	Tween 20	0.72 mg/mL		[157]
		*S. aureus* (ATCC 29213)				
	MIC	*S. aureus* (strains isolated from skin lesions)	Ethanol	0.25–1.50 mL/mL	Citronellol (26.70%), geraniol (13.40%)	[166]
		*S. aureus* (strains isolated postoperatively)		0.50–2.25 mL/mL		
		MRSA and MSSA (clinical strains)		1.00 mL/mL		
		*S. aureus* (ATCC 6538)	Acetone	1.50 mg/mL	Citronellol (34.20%), geraniol (15.70%)	[99]

*Perovskia abrotanoides* (Russian sage)	MIC	*S. aureus* (ATCC 25923)	10% DMSO	8.00 *μ*L/mL	Camphor (23.00%), 1,8-cineole (22.00%), *α*-pinene (12.00%)	[167]

*Pimpinella anisum* (anise)	MIC	*S. aureus*	DMSO	125.00 *μ*g/mL	NCR	[168]
				≤1.00 *μ*g/mL	Anethole (64.82%)	[118]

*Pinus sylvestris* (pine)	MIC	*S. aureus* (ATCC 6538)	Acetone	4.00 mg/mL	Bornyl acetate (42.30%), camphene (11.80%), *α*-pinene (11.00%)	[99]

*Piper nigrum* (black pepper)	MIC	*S. aureus* (ATCC 6538)	Acetone	2.00 mg/mL	*β*-Caryophyllene (33.80%), limonene (16.40%)	[99]

*Pogostemon cablin* (patchouli)	MIC	*S. aureus* (NCTC 6571)	10% DSMO	395.20 *μ*g/mL	*α*-Guaiene (13.80%), *α*-bulnesene (17.10%), patchouli alcohol (22.70%)	[139]
		*S. aureus* (NCTC 1803)		520.00 *μ*g/mL		

*Pogostemon patchouli* (patchouli)	MIC	*S. aureus* (ATCC 6538)	Acetone	1.50 mg/mL	Patchouli alcohol (37.30%), *α*-bulnesene (14.60%), *α*-guaiene (12.50%)	[99]

*Rosmarinus officinalis* (rosemary)	MIC	*S. aureus* (ATCC 6538)	Tween 80	0.13% v/v	1,8-Cineole (27.23%), *α*-pinene (19.43%), camphor (14.26%), camphene (11.52%)	[169]
		*S. aureus* (NCTC 6571)	10% DSMO	305.30 *μ*g/mL	1,8-Cineol (29.2%), (+)-camphor (17.2%)	[139]
		*S. aureus* (NCTC 1803)		310.40 *μ*g/mL		
		MRSA (clinical isolate)	Tween 80	0.03% v/v	1,8-Cineole (26.54%), *α*-pinene (20.14%), camphene (11.38%), camphor (12.88%)	[170]
		*S. aureus* (MTCC 96)	n.m.	>11.00 mg/mL	NCR	[171]
		*S. aureus* (ATCC 6538)	Hexane	1.88–7.50 mg/mL	1,8-Cineole (10.56–11.91%), camphor (16.57–16.89%), verbenone (17.43–23.79%)	[172]
	ADM	*S. aureus* (ATCC 25923)	10% DMSO	>12.80 mg/mL	NCR	[80]
		*S. aureus* (ATCC 6538)	Tween 20	5.60 mg/mL		[157]
		*S. aureus* (ATCC 29213)				
	MIC	*S. aureus* (ATCC 12600)	Acetone	6.20 mg/mL	1,8-Cineole (41.40%), *α*-pinene (13.30%), camphor (12.40%)	[140]
		*S. aureus* (ATCC 43387)	DMSO	0.20% v/v	NCR	[129]
		*S. aureus* (ATCC 6538)	Acetone	4.00 mg/mL	1,8-Cineole (48.00%)	[99]
	ADM_90_	*S. aureus* (ATCC 25923, 16 MRSA and 15 MSSA clinical isolates)	Tween 80	8.60 mg/mL	Camphor (27.51%), limonene (21.01%), myrcene (11.19%), *α*-pinene (10.37%)	[119]

*Salvia bracteata* (sage)	MIC	*S. aureus* (ATCC 25923)		50.00 *μ*g/mL	Caryophyllene oxide (16.60%)	[173]
*Salvia eremophila* (sage)	MIC	*S. aureus* (ATCC 29737)	10% DMSO	8.00 *μ*g/mL	Borneol (21.83%), *α*-pinene (18.80%), bornyl acetate (18.68%)	[174]
*Salvia nilotica* (sage)	ADM	*S. aureus* (ATCC 25923)	n.m.	5.40 mg/mL	*trans*-Caryophyllene (10.90%)	[175]

*Salvia officinalis* (sage)	MIC	*S. aureus* (NCTC 6571)	10% DSMO	302.40 *μ*g/mL	1,8-Cineol (27.40%), *α*-thujone (16.30%), *β*-thujone (11.20%), borneol (10.40%)	[139]
		*S. aureus* (NCTC 1803)		324.30 *μ*g/mL		
		*S. aureus* (ATCC 43387)	DMSO	0.20% v/v	NCR	[129]
	ADM	*S. aureus* (ATCC 6538)	Tween 20	11.20 mg/mL	NCR	[157]
		*S. aureus* (ATCC 29213)		5.60 mg/mL		
		*S. aureus* (ATCC 25923)	n.m.	7.50 mg/mL		[176]

*Salvia ringens* (sage)	MIC	*S. aureus* (ATCC 25923)	n.m.	NI	*α*-Pinene (12.85%), 1,8-cineole (46.42%)	[177]
*Salvia rosifolia* (sage) (3 samples)		MRSA	20% DMSO	125.00–1000.00 *μ*g/mL	*α*-Pinene (15.70–34.80%), 1,8-cineole (16.60–25.10%), *β*-pinene (6.70–13.50%)	[178]
*Salvia rubifolia* (sage)		*S. aureus* (ATCC 25923)	Tween 20	50.00 *μ*g/mL	*γ*-Muurolene (11.80%)	[173]

*Salvia sclarea* (clary sage)	MIC	*S. aureus* (11 MRSA and 16 MSSA)	Ethanol	3.75–5.25	Linalyl acetate (57.90%), linalool (12.40%)	[179]
		*S. aureus* (ATCC 6538)	Acetone	2.00 mg/mL	Linalyl acetate (72.90%), linalool (11.90%)	[99]

*Santalum album* (sandalwood)	MIC	*S. aureus* (ATCC 6538)	Acetone	0.25 mg/mL	*α*-Santalol (32.10%)	[99]

*Styrax benzoin* (benzoin)	MIC	*S. aureus* (ATCC 6538)	Acetone	2.00 mg/mL	cinnamyl alcohol (44.80%), benzene propanol (21.70%)	[99]

*Syzygium aromaticum* (clove)	MIC	*S. aureus* (ATCC 6538)	Tween 80	0.13% v/v	Eugenol (68.52%), *β*-caryophyllene (19.00%), 2-methoxy-4-[2-propenyl]phenol acetate (10.15%)	[169]
		*S. aureus*	DMSO	≤1.00 *μ*g/mL	Eugenol (84.07%), isoeugenol (10.39%)	[118]
	ADM	*S. aureus* (ATCC 25923)	10% DMSO	>6.40 mg/mL	NCR	[80]
	MIC	*S. aureus* (ATCC 6538)	Acetone	1.50 mg/mL	Eugenol (82.20%), eugenol acetate (13.20%)	[99]

*Tagetes minuta* (Mexican marigold)	MIC_90_	*S. aureus* (ATCC 6538)	n.m.	67.00 *μ*g/mL	Dihydrotagetone (33.90%), *E*-ocimene (19.90%), tagetone (16.10%)	[159]
*Tagetes patula* (French marigold)	MIC	*S. aureus* (ATCC 6538)	Acetone	4.00 mg/mL	*(E)*-*β*-Ocimene (41.30%), *E*-tagetone (11.20%), verbenone (10.90%)	[99]

*Thymbra spicata* (thyme)	MIC	*S. aureus* (ATCC 29213)	Tween 80	2.25 mg/mL	Carvacrol (60.39%), *γ*-terpinene (12.95%)	[180]
*Thymus capitatus* (thyme)		*S. aureus* (ATCC 25923)		900.00 *μ*g/mL	*p*-Cymene (26.40%), thymol (29.30%), carvacrol (10.80%)	[181]
*Thymus capitatus* (thyme), commercial					*α*-Pinene (25.20%), linalool (10.30%), thymol (46.10%)	
*Thymus herba-barona* (thyme), Gennargentu				225.00 *μ*g/mL	Thymol (46.90%), carvacrol (20.60%)	
*Thymus herba-barona* (thyme), Limbara				900.00 *μ*g/mL	*p*-Cymene (27.60%), thymol (50.30%)	

*Thymus hyemalis* (thyme) (thymol, thymol/linalool, carvacrol chemotypes)	MAC	*S. aureus* (CECT 239)	95% ethanol	<0.20–0.50 *μ*L/mL	*p*-Cymene (16.00–19.80%), linalool (2.10–16.60%), thymol (2.90–43.00%), carvacrol (0.30–40.10%)	[61]

*Thymus numidicus*	ADM	*S. aureus* (ATCC 25923)	n.m.	0.23 mg/mL	NCR	[176]
*Thymus serpyllum* (thyme)		*S. aureus* (ATCC 6538)	Tween 20	0.28 mg/mL		[157]
		*S. aureus* (ATCC 29213)		0.70 mg/mL		

*Thymus vulgaris* (thyme)	MIC	*S. aureus*	DMSO	31.20 *μ*g/mL	NCR	[168]
		*S. aureus* (NCTC 6571)	10% DSMO	160.50 *μ*g/mL	*p*-Cymene (17.90%), thymol (52.40%)	[139]
		*S. aureus* (NCTC 1803)		210.00 *μ*g/mL		
		*S. aureus* (ATCC 25923)	n.m.	0.40 mg/mL	NCR	[85]
	ADM	*S. aureus* (ATCC 433000)	Ethanol	0.25 *μ*L/mL	Thymol (38.1%), *p*-cymene (29.10%)	[182]
		*S. aureus* (2 multidrug-resistant clinical strains from hands)		0.50 *μ*L/mL	Thymol (38.1%), *p*-cymene (29.10%)	[182]
		*S. aureus* (6 multidrug-resistant clinical strains from wounds)		0.50–1.00 *μ*L/mL		
		*S. aureus* (4 multidrug-resistant clinical strains from ulcers)		0.50–0.75 *μ*L/mL		
		*S. aureus* (multidrug-resistant clinical strain from abscesses)		0.25 *μ*L/mL		
	MIC	*S. aureus* (ATCC 12600)	Acetone	1.30 mg/mL	Thymol (47.20%), *p*-cymene (22.10%)	[140]
		MRSA (15 clinical isolates)	Ethanol	0.50% v/v	NCR	[136]
	ADM	MRSA (ATCC 33592)	Tween 20	18.50 *μ*g/mL	Thymol (48.1%), *p*-cymene (15.60%), *γ*-terpinene (15.40%)	[81]
		*S. aureus* (ATCC 25922)				
		MRSA (14 clinical isolates)		18.50–37.00 *μ*g/mL		
	MIC	*S. aureus* (ATCC 6538)	Acetone	3.33 mg/mL	*p*-Cymene (39.90%), thymol (20.70%)	[99]

*Thymus vulgaris* (thyme) (thymol chemotype)	MAC	*S.aureus* (CECT 239)	95% ethanol	<0.20 *μ*L/mL	*p*-Cymene (18.70%), thymol (57.70%)	[61]
*Thymus zygis* subsp. *gracilis* (thyme) (thymol and two linalool chemotypes)				<0.20–1.20 *μ*L/mL	*p*-Cymene (0.50–11.20%), *(E)*-sabinene hydrate (0.20–18.20%), linalool (2.00–82.30%)	

*Vetiveria zizanioides/Andropogon muricatus *(vetiver)	MIC	*S. aureus* (ATCC 6538)	Acetone	0.75 mg/mL	Zizanol (13.6%), *β*-vetirenene (7.2%)	[99]

^a^Scientific name (common name), part of plant (if applicable).

^b^MIC: microdilution method; MAC: macrodilution method; ADM: agar dilution method; CTA: contact time assay.

^c^American Type Culture Collection, Rockville, USA (ATCC); Colección Espanõla de Cultivos Tipo (CECT); collection of microorganisms of the Department of Microbiology (MFBF); culture collection of antibiotics resistant microbes (CCRM); Eskişehir Osmangazi University, Faculty of Medicine, clinical isolate (OGU); Laboratorio de Microbiología, Facultad de Ciencias Médicas, Universidad Nacional de Cuyo, Mendoza, Argentina (LM); Microbial Type Culture Collection (MTCC); Mycology Laboratory (LM); National Center of Industrial Microorganisms (NCIM); National Collection of Type Cultures, London, Great Britain (NCTC); Spanish Collection of Type Cultures (STCC).

^d^DMSO concentration was not included; n.m.: not mentioned.

^e^NI: no inhibition.

^f^NCR: no composition results reported.

**Table 4:** Antimicrobial efficacy of essential oils against pathogens associated with acne.

Essential oil^a^	Method^b^	Species strain^c^	Solvent^d^	Result^e^	Main components^f^	Reference
*Abies koreana* (Korean fir)	MIC	*S. epidermidis* (antibiotic-susceptible strain SK4)	n.m.	0.63 *μ*L/mL	Bornyl acetate (30.40%), limonene (19.00%)	[208]
		*S. epidermidis* (antibiotic-resistant strain SK9)		0.31 *μ*L/mL		
		*S. epidermidis* (antibiotic-resistant strain SK19)		5.00 *μ*L/mL		
		*P. acnes* (ATCC 3314)		0.31 *μ*L/mL		
		*P. acnes* (antibiotic-resistant strain SKA 4)				
		*P. acnes* (antibiotic-resistant strain SKA 7)		0.63 *μ*L/mL		

*Anthemis aciphylla *var.* discoidea* (chamomile), flowers	MIC	*S. epidermidis* (ATCC 12228)	DMSO	0.25 mg/mL	*α*-Pinene (39.00%), terpinen-4-ol (32.10%)	[114]
*Anthemis aciphylla *var.* discoidea* (chamomile), aerial parts				0.13 mg/mL	*α*-Pinene (49.40%), terpinen-4-ol (21.80%)	
*Anthemis aciphylla *var.* discoidea* (chamomile), leaves				0.06 mg/mL	Terpinen-4-ol (24.30%)	

*Anthemis nobilis* (chamomile)	MIC	*P. acnes* (CMCC 65002)	Tween 80	0.13% v/v	NCR	[209]

*Cananga odorata *var.* fruticosa* (dwarf ylang-ylang) *Cananga odorata* (ylang-ylang)	ADM	*P. acnes* (DMST 4916, 14917, 4918, 21823, 21823, 21824)	0.5% polysorbate 80	>4.00% v/v	NCR	[210]

*Cinnamomum burmannii* (cinnamon stick)	MIC	*S. epidermidis* (16 clinical isolates)	5% propylene glycol (PG)	0.50–2.00%	Cinnamaldehyde	[211]
		*S. epidermidis* strains RP62A (ATCC 35984)		1.00%		
		*S. epidermidis* (ATCC 12228)		0.50%		

*Cinnamomum zeylanicum* (cinnamon)	MIC	*P. acnes* (CMCC 65002)	Tween 80	0.012% v/v	NCR	[209]

*Citrus aurantium* (bitter orange), flowers	MIC	*S. epidermidis* (ATCC 12228)	50% DMSO	1.25 mg/mL	Limonene (27.50%), *E*-nerolidol (17.5%), *α*-terpineol (14.00%)	[120]

*Citrus medica limonum* (lemon)	MIC	*P. acnes* (CMCC 65002)	Tween 80	0.25% v/v	NCR	[209]

*Citrus natsudaidai* (Japanese summer orange)	MIC	*S. epidermidis* (KCTC 3958)	5% Tween 80	10.00 *μ*L/mL	Limonene (81.60%)	[102]
		*P. acnes* (ATCC 6919)		0.31 *μ*L/mL		

*Citrus paradisi* (grapefruit)	MIC	*P. acnes* (CMCC 65002)	Tween 80	0.25% v/v	NCR	[209]
	MAC	*S. epidermidis* (KCTC 3958)		>50.00 *μ*L/mL	Limonene (55.40–91.70%), myrcene (2.10–32.10%)	[212]

*Citrus *species (citrus) (14 spp.)	MAC	*S. epidermidis* (KCTC 3958)	Tween 80	>50.00 *μ*L/mL	Limonene (55.40–91.70%), myrcene (2.10–32.10%)	[212]
		*P. acnes* (ATCC 6919)		1.25–>50.00 *μ*L/mL		

*Coriandrum sativum* (coriander)	ADM	*P. acnes* (DMST 4916, 14917, 4918, 21823, 21823, 21824)	0.5% polysorbate 80	1.00% v/v	NCR	[210]
*Curcuma longa* (turmeric)				>4.00% v/v		
*Cymbopogon citratus* (lemongrass)				0.13% v/v		
*Cymbopogon nardus* (citronella)						

*Eucalyptus globulus* (eucalyptus)	MIC	*S. epidermidis* (ATCC 14990)	Tween 80	10.00 mg/mL	1,8-Cineol (81.93%)	[128]
		*S. epidermidis* (RP62A)	5% DSMO	4.00 mg/L	NCR	[213]
		*S. epidermidis* (clinical isolate TK1)		8.00 mg/L		
		*P. acnes* (DMST 14917)	n.m.	9.38 mg/mL	*p*-Cymene (28.75%), *γ*-terpinene (44.60%)	[214]
	ADM	*P. acnes* (DMST 4916, 14917, 4918, 21823, 21823, 21824)	0.5% polysorbate 80	4.00% v/v	NCR	[210]

*Eucalyptus intertexta* (eucalyptus)	MIC	*S. epidermidis* (ATCC 12228)	10% DSMO	7.80 *μ*g/mL	NCR	[131]
*Eucalyptus largiflorens* (eucalyptus)				125.00 *μ*g/mL		

*Foeniculum vulgare* (fennel), Aurelio	MAC	*S. epidermidis* (ATCC 12228)	Tween 20	50.00 *μ*g/mL	Limonene (16.50–21.50%), *(E)*-anethole (59.80–66.00%)	[134]
*Foeniculum vulgare* (fennel), Spartaco				25.00–50.00 *μ*g/mL	Limonene (0.20–17.70%), *(E)*-anethole (66.30–90.40%)	
*Foeniculum vulgare* (fennel) (6 samples)	MIC		DMSO	250.00–750.00 *μ*g/mL	Fenchone (16.90–34.70%), estragole (2.50–66.00%), *trans*-anethole (7.90–77.70%)	[133]

*Jasminum grandiflora* (jasmine)	MIC	*P. acnes* (CMCC 65002)	Tween 80	0.50% v/v	NCR	[209]
*Jasminum sambac* (jasmine)	ADM	*P. acnes* (DMST 4916, 14917, 4918, 21823, 21823, 21824)	0.5% polysorbate 80	2.00% v/v		[210]

*Juniperi aetheroleum* (juniper)	MAC_80_	*S. epidermidis* (MFBF)	n.m.	40.00% v/v	*α*-Pinene (29.17%), *β*-pinene (17.84%), sabinene (13.55%)	[135]
*Juniperus communis* (juniper)	CTA	*P. acne*s (ATCC 6919)	PEG 200	2.00 mg/mL	*α*-Pinene (22.75%), *β*-myrcene (11.88%)	[215]
*Juniperus officinalis* (juniper), berry	MIC	*S. epidermidis* (ATCC 14990)	Tween 80	20.00 mg/mL	*α*-Pinene (39.76%)	[128]

*Kunzea ericoides* (Kānuka)	MAC	*S. epidermidis* (clinical isolate)	Tween 80	0.25% v/v	*α*-Pinene (61.60%)	[137]
	MIC	*S. epidermidis* (ATCC 2223)	Acetone	8.00 mg/mL	*α*-Pinene (26.2–46.7%), p-cymene (5.8–19.1%)	[138]
	MIC	*P. acnes* (ATCC 11827)	Acetone	4.00 mg/mL		

*Lavandula angustifolia* (lavender)	MIC	*S. epidermidis* (ATCC 2223)	Acetone	6.20 mg/mL	Linalool (30.80%), linalyl acetate (31.30%)	[140]
		*S. epidermidis* (antibiotic-susceptible strain SK4)	n.m.	1.00 *μ*L/mL	NCR	[208]
		*S. epidermidis* (antibiotic-resistant strain SK9)	n.m.	0.13 *μ*L/mL		
		*S. epidermidis* (antibiotic-resistant strain SK19)	n.m.	1.00 *μ*L/mL		
		*P. acnes* (ATCC 3314)	n.m.	0.25 *μ*L/mL		
		*P. acnes* (antibiotic-resistant strain SKA 4)	n.m.	1.25 *μ*L/mL		
		*P. acnes* (antibiotic-resistant strain SKA 7)	n.m.	0.25 *μ*L/mL		
	ADM	*P. acnes* (DMST 4916, 14917, 4918, 21823, 21823, 21824)	0.5% polysorbate 80	2.00% v/v	NCR	[210]

*Lavandula stoechas* (French lavender), flower	MIC	*S. epidermidis* (ATCC 12228)	20% DMSO	250.00 *μ*g/mL	*α*-Fenchone (39.20%)	[47]
*Lavandula stoechas* (French lavender), leaf	MIC				*α*-Fenchone (41.90%), 1,8-cineole (15.60%), camphor (12.10%)	
*Lavandula stoechas* (lavender)	MIC	*P. acnes* (CMCC 65002)	Tween 80	0.13% v/v	NCR	[209]

*Leptospermum scoparium* (manuka)	MAC	*S. epidermidis* (clinical isolate)	Tween 80	0.05% v/v	(−)-*(E)*-Calamenene (14.50%), leptospermone (17.60%)	[137]
	MIC	*S. epidermidis* (ATCC 2223)	Acetone	4.00 mg/mL	Eudesma-4(14),11-diene (6.20–14.50%), *α*-selinene (5.90–13.50%), *(E)*-methyl cinnamate (9.20–19.50%)	[138]
		*P. acnes* (ATCC 11827)		1.00 mg/mL		

*Melaleuca alternifolia* (tea tree)	ADM	*S. epidermidis* (NCIM number 2493)	Tween 80	1.00%	NCR	[79]
		*S. epidermidis* (clinical isolate)				
	MAC	*S. epidermidis* (clinical isolate)		0.45% v/v	*α*-Terpinene (11.40%), *γ*-terpinene (22.50%), terpinen-4-ol (35.20%)	[137]
	MIC	*S. epidermidis* (15 clinical isolates)		0.12–1.00% v/v	Terpinen-4-ol (35.70%)	[152]
	MIC	*S. epidermidis* (RP62A)	5% DSMO	2.00 mg/L	NCR	[213]
		*S. epidermidis* (clinical isolate TK1)		16.00 mg/L		
	MIC	*S. epidermidis* (ATCC 2223)	Acetone	6.20 mg/mL	Terpinen-4-ol (38.60%), *γ*-terpinene (21.60%)	[140]
	MIC	*S. epidermidis* (antibiotic-susceptible strain SK4)	n.m.	0.13 *μ*L/mL	NCR	[208]
		*S. epidermidis* (antibiotic-resistant strain SK9)		1.00 *μ*L/mL		
		*S. epidermidis* (antibiotic-resistant strain SK19)				
	MAC	*P. acnes* (MTCC 1951)	Tween 80	0.50% v/v	NCR	[79]
		*P. acnes* (32 clinical strains)		0.25–0.50%	Terpinen-4-ol (35.70%)	[92]
	ADM	*P. acnes* (DMST 4916, 14917, 4918, 21823, 21823, 21824)	0.5% polysorbate 80	1.00% v/v	NCR	[210]
	MIC	*P. acnes* (ATCC 3314)	n.m.	0.25 *μ*L/mL	NCR	[208]
		*P. acnes* (antibiotic-resistant strain SKA 4)		2.50 *μ*L/mL		
		*P. acnes* (antibiotic-resistant strain SKA 7)		0.25 *μ*L/mL		
	MAC	*S. epidermidis* (NCTC 11047)	n.m.	0.63–1.25% v/v	*α*-Pinene (11.95%), *α*-terpinene (14.63%), terpinen-4-ol (29.5%), *p*-cymene (17.74%)	[154]
		*P. acnes* (NCTC 737)		0.31–0.63% v/v		
		*S. epidermidis* (NCTC 11047)		0.63–1.25% v/v	*α*-Pinene (24.87%), *α*-terpinene (12.47%), terpinen-4-ol (28.59%)	
		*P. acnes* (NCTC 737)		0.31–0.63% v/v		

*Melaleuca cajuputi* (cajuput)	MAC	*S. epidermidis* (clinical isolate)	Tween 80	0.40% v/v	1,8-Cineole (55.50%)	[137]
	MIC	*S. epidermidis* (ATCC 14990)	Tween 80	10.00 mg/mL	1,8-Cineol (67.60%)	[128]

*Melaleuca quinquenervia* (niaouli)	MAC	*S. epidermidis* (clinical isolate)	tween 80	0.40% v/v	1,8-Cineole (61.20%)	[137]

*Mentha piperita* (peppermint)	MIC	*S. epidermidis* (ATCC 14990)	Tween 80	1.25 mg/mL	1,8-Cineol (12.06%), menthone (22.24%), menthol (47.29%)	[128]
		*S. epidermidis* (NCTC 12228)	DMSO	0.63–2.50 mg/mL	Menthol (27.50–42.30%), menthone (18.40–27.90%)	[155]
		*S. epidermidis* (ATCC 2223)	Acetone	6.20 mg/mL	Menthone (18.20%), menthol (42.90%)	[140]
		*S. epidermidis* (antibiotic-susceptible strain SK4)	n.m.	0.13 *μ*L/mL	NCR	[208]
		*S. epidermidis* (antibiotic-resistant strain SK9)		0.50 *μ*L/mL		
		*S. epidermidis* (antibiotic-resistant strain SK19)				
		*P. acnes* (ATCC 3314)		0.25 *μ*L/mL		
		*P. acnes* (antibiotic-resistant strain SKA 4)		0.63 *μ*L/mL		
		*P. acnes* (antibiotic-resistant strain SKA 7)		0.06 *μ*L/mL		

*Mentha spicata* (spearmint)	MIC	*P. acnes* (CMCC 65002)	Tween 80	0.25% v/v	NCR	[209]

*Ocimum americanum* (hoary basil)	ADM	*P. acnes* (DMST 14916)	Polysorbate 80	>5.00% v/v	Neral (27.20%), geraniol (32.00%)	[44]

*Ocimum basilicum* (basil)	MAC	*S. epidermidis* (2 clinical strains)	Tween 80	(0.15–0.30) × 10^−2^% v/v	Linalool (54.95%), methyl chavicol (11.98%)	[158]
	ADM	*P. acnes* (DMST 14916)	Polysorbate 80	2.00% v/v	Methyl chavicol (93.00%)	[44]
		*P. acnes* (DMST 4916, 14917, 4918, 21823, 21823, 21824)	0.5% polysorbate 80	>4.00% v/v	NCR	[210]

*Ocimum sanctum* (holy basil)	ADM	*P. acnes* (DMST 14916)	Polysorbate 80	3.0% v/v	Eugenol (41.50%), methyl eugenol (11.80%), *γ*-caryophyllene (23.70%)	[44]
*Ocimum tenuiflorum* (holy basil)		*P. acnes* (DMST 4916, 14917, 4918, 21823, 21823, 21824)	0.5% polysorbate 80	2.00% v/v	NCR	[210]

*Origanum acutidens* (Turkey oregano)	MIC	*S. epidermis* (A233)	10% DMSO	125.00 *μ*g/mL	Carvacrol (72.0%)	[161]
*Origanum microphyllum* (oregano)		*S. epidermidis* (ATCC 12228)	Tween 80	5.32 mg/mL	Terpin-4-ol (24.86%), *γ*-terpinene (13.83%), linalool (10.81%)	[162]
*Origanum scabrum* (oregano)				0.38 mg/mL	Carvacrol (74.86%)	

*Origanum vulgare* (oregano)	ADM	*S. epidermidis* (ATCC 12228)	1% DMSO	0.13% v/v	*p*-Cymene (14.60%), *γ*-terpinene (11.70%), thymol (24.70%), carvacrol (14.00%)	[163]
	ADM	*S. epidermidis* (21 clinical isolates)		0.06–0.13% v/v		

*Piper nigrum* (black pepper)	ADM	*P. acnes* (DMST 4916, 14917, 4918, 21823, 21823, 21824)	0.5% polysorbate 80	>4.00% v/v	NCR	[210]

*Rosa centifolia* (rose)	MIC	*P. acnes* (CMCC 65002)	Tween 80	0.03% v/v	NCR	[209]

*Rosmarinus officinalis* (rosemary)	MIC	*S. epidermidis* (ATCC 12228)	Tween 80	0.25% v/v	1,8-Cineole (27.23%), *α*-pinene (19.43%), camphor (14.26%), camphene (11.52%)	[169]
		*S. epidermidis*		0.10% (v/v)	1,8-Cineole (26.54%), *α*-pinene (20.14%), camphene (11.38%), camphor (12.88%)	[170]
		*S. epidermidis* (MTCC 435)	n.m.	>11.00 mg/mL	NCR	[171]
		*S. epidermidis* (ATCC 2223)	Acetone	10.10 mg/mL	1,8-Cineole (41.40%), *α*-pinene (13.30%), camphor (12.40%)	[140]

*Salvia bracteata* (sage)	MAC	*S. epidermidis* (ATCC 12228)	Tween 20	50.00 *μ*g/mL	Caryophyllene oxide (16.6%)	[173]

*Salvia eremophila* (sage)	MIC	*S. epidermidis* (ATCC 12228)	10% DMSO	32.00 *μ*g/mL	Borneol (21.83%), *α*-pinene (18.80%), bornyl acetate (18.68%)	[174]

*Salvia nilotica* (sage)	ADM	*S. epidermidis* (ATCC 12228)	n.m.	5.50 mg/mL	*trans*-Caryophyllene (10.90%)	[175]

*Salvia ringens* (sage)	MIC	*S. epidermidis* (ATCC 12228)	n.m.	NI	*α*-Pinene (12.85%), 1,8-cineole (46.42%)	[177]

*Salvia rosifolia* (sage) (3 samples)	MIC	*S. epidermidis* (ATCC 12228)	20% DMSO	125.00–1000.00 *μ*g/mL	*α*-Pinene (15.70–34.80%), 1,8-cineole (16.60–25.10%), *β*-pinene (6.70–13.50%)	[178]

*Salvia rubifolia* (sage)	MAC	*S. epidermidis* (ATCC 12228)	Tween 20	50.00 *μ*g/mL	*γ*-Muurolene (11.80%)	[173]

*Salvia sclarea* (clary sage)	MIC	*S. epidermidis* (19 clinical isolates)	Ethanol	4.50–6.25	Linalyl acetate (57.90%), linalool (12.40%)	[179]

*Syzygium aromaticum* (clove)	MIC	*S. epidermidis* (ATCC 12228)	Tween 80	0.25% v/v	Eugenol (68.52%), *β*-caryophyllene (19.00%), 2-methoxy-4-[2-propenyl]phenol acetate (10.15%)	[169]
	ADM	*P. acnes* (DMST 4916, 14917, 4918, 21823, 21823, 21824)	0.5% polysorbate 80		NCR	[210]
	MIC	*P. acnes*	n.m.	0.31 mg/mL	DL-Limonene (61.60%)	[216]

*Thymus capitatus* (thyme)	MIC	*S. epidermidis* (ATCC 12228)	Tween 80	900.00 *μ*g/mL	*p*-Cymene (26.40%), thymol (29.30%), carvacrol (10.80%)	[181]
*Thymus capitatus* (thyme), commercial					*α*-Pinene (25.20%), linalool (10.30%), thymol (46.10%)	
*Thymus herba-barona* (thyme), Gennargentu				450.00 *μ*g/mL	Thymol (46.90%), carvacrol (20.6 0%)	
*Thymus herba-barona* (thyme), Limbara				900.00 *μ*g/mL	*p*-Cymene (27.60%), thymol (50.30%)	

*Thymus quinquecostatus* (thyme), Jeju	MAC	*P. acnes* (ATCC 6919)	n.m.	0.50 mg/mL	*p*-Cymen-3-ol (50.41%), *p*-cymen-2-ol (24.06%), cymene (19.04%)	[207]
		*P. granulosum* (ATCC 25564)				

*Thymus vulgaris* (thyme)	MIC	*S. epidermidis* (ATCC 2223)	Acetone	4.70 mg/mL	Thymol (47.20%), *p-*cymene (22.10%)	[140]
		*P. acnes* (CMCC 65002)	Tween 80	0.02% v/v	NCR	[209]

*Zingiber officinale* (ginger)	MIC	*P. acnes* (CMCC 65002)	Tween 80	0.25% v/v	NCR	[209]

*Zingiber officinale *Roscoe (ginger)	ADM	*P. acnes* (DMST 4916, 14917, 4918, 21823, 21823, 21824)	0.5% polysorbate 80	>4.00% v/v	NCR	[210]

^a^Scientific name (common name), part of plant (if applicable).

^b^MIC: microdilution method; MAC: macrodilution method; ADM: agar dilution method; CTA: contact time assay.

^c^American Type Culture Collection, Rockville, USA (ATCC); Colección Espanõla de Cultivos Tipo (CECT); collection of microorganisms of the Department of Microbiology (MFBF); culture collection of antibiotics-resistant microbes (CCRM); Eskişehir Osmangazi University, Faculty of Medicine, clinical isolate (OGU); Laboratorio de Microbiología, Facultad de Ciencias Médicas, Universidad Nacional de Cuyo, Mendoza, Argentina (LM); Microbial Type Culture Collection (MTCC); Mycology Laboratory (LM); National Center of Industrial Microorganisms (NCIM); National Collection of Type Cultures, London, Great Britain (NCTC); Spanish Collection of Type Cultures (STCC).

^d^DMSO concentration was not included; n.m.: not mentioned.

^e^NI: no inhibition.

^f^NCR: no composition results reported.

**Table 5:** Essential oil studies showing efficacy against Gram-negative pathogens associated with skin infections.

Essential oil^a^	Method^b^	Species strain^c^	Solvent^d^	Result^e^	Main components^f^	Reference
*Abies balsamea* (fir)	MIC	*P. aeruginosa* (ATCC 27858)	Acetone	2.00 mg/mL	*β*-Pinene (31.00%), bornyl acetate (14.90%), *δ*-3-carene (14.20%)	[99]

*Abies holophylla* (Manchurian fir)	MIC	*E. coli* (ATCC 10536)	DMSO	21.8 mg/mL	Bicyclo[2.2.1]heptan-2-ol (28.05%), *δ*-3-carene (13.85%), *α*-pinene (11.68%), camphene (10.41%)	[111]
		*E. coli* (ATCC 25922)				
		*E. coli* (ATCC 33312)				
		*P. aeruginosa* (NCTC 10490)		>21.80 mg/mL		

*Abies koreana* (Korean fir)	MIC	*E. coli* (ATCC 10536)	DMSO	21.8 mg/mL	Bornyl ester (41.79%), camphene (15.31%), *α*-pinene (11.19%)	[111]
		*E. coli* (ATCC 25922)				
		*E. coli* (ATCC 33312)		10.9 mg/mL		
		*P. aeruginosa* (NCTC 10490)		>21.80 mg/mL		

*Achillea millefolium* (yarrow)	MIC	*P. aeruginosa* (ATCC 27853)	Tween 80	NI	Eucalyptol (24.60%), camphor (16.70%), *α*-terpineol (10.20%)	[112]
		*E. coli* (ATCC 25922)				
*Achillea setacea* (bristly yarrow)		*E. coli* (ATCC 25922)	0.5% Tween 80	72.00 mg/mL	Sabinene (10.80%), eucalyptol (18.50%)	[113]
		*P. aeruginosa* (ATCC 27853)		>72.00 mg/mL		
*Achillea teretifolia* (bristly yarrow)		*E. coli* (ATCC 25922)		36.00 mg/mL	Eucalyptol (19.90%), camphor (11.10%), borneol (11.90%)	
		*P. aeruginosa* (ATCC 27853)		>72.00 mg/mL		

*Angelica archangelica* (angelica) root	MIC	*P. aeruginosa* (ATCC 27858)	Acetone	2.00 mg/mL	*α*-Phellandrene (18.50%), *α*-pinene (13.70%), *β*-phellandrene (12.60%), *δ*-3-carene (12.10%)	[99]
*Angelica archangelica* (angelica) seed					*β*-Phellandrene (59.20%)	

*Aniba rosaeodora* (rosewood)	MIC	*E. coli* (ATCC 25922)	n.m.	0.40 mg/mL	NCR	[85]

*Anthemis aciphylla *var.* discoidea* (chamomile), flowers	MIC	*E. coli* (ATCC 25922)	DMSO	1.00 mg/mL	*α*-Pinene (39.00%), terpinen-4-ol (32.10%)	[114]
		*P. aeruginosa* (ATCC 27853)		0.25 mg/mL		

*Anthemis aciphylla *var.* discoidea* (chamomile), aerial parts	MIC	*E. coli* (ATCC 25922)	DMSO	1.00 mg/mL	*α*-Pinene (49.40%), terpinen-4-ol (21.80%)	[114]
		*P. aeruginosa* (ATCC 27853)				

*Anthemis aciphylla *var.* discoidea* (chamomile), leaves	MIC	*E. coli* (ATCC 25922)	DMSO	0.50 mg/mL	Terpinen-4-ol (24.30%)	[114]
		*P. aeruginosa* (ATCC 27853)				

*Anthemis nobilis* (chamomile)	MIC	*P. aeruginosa* (ATCC 27858)	Acetone	2.00 mg/mL	2-Methylbutyl-2-methyl propanoic acid (31.50%), limonene (18.30%), 3-methylpentyl-2-butenoic acid (16.70%), isobutyl isobutyrate (10.00%)	[99]

*Artemisia dracunculus* (tarragon)	MIC	*P. aeruginosa* (ATCC 27858)	Acetone	2.00 mg/mL	Estragole (82.60%)	[99]

*Backhousia citriodora* (lemon myrtle)	ADM	*E. coli* (NCTC 8196)	Tween 80	0.03% v/v	Geranial (51.40%), neral (40.90%)	[115]
		*P. aeruginosa* (NCTC 6750)		2.00% v/v		

*Boswellia carteri* (frankincense) (9 samples)	MIC	*E. coli* (ATCC 25922)	Acetone	4.00–12.00 mg/mL	*α*-Pinene (4.80–40.40%), myrcene (1.60–52.40%), limonene (1.90–20.40%), *α*-thujene (0.30–52.40%), *p*-cymene (2.70–16.90%), *β*-pinene (0.30–13.10%)	[116]
*Boswellia frereana* (frankincense) (3 samples)				4.00–6.00 mg/mL	*α*-Pinene (2.00–64.70%), *α*-thujene (0.00–33.10%), *p*-cymene (5.40–16.90%)	
*Boswellia neglecta* (frankincense)		*E. coli* (ATCC 8739)	Acetone	3.00 mg/mL	NCR	[117]
		*E. coli* (ATCC 25922)		6.00 mg/mL	*α*-Pinene (43.40%), *β*-pinene (13.10%)	[116]
		*P. aeruginosa* (ATCC 27858)		1.30 mg/mL	NCR	[117]
*Boswellia papyrifera* (frankincense)		*E. coli* (ATCC 8739)		3.30 mg/mL		
		*P. aeruginosa* (ATCC 27858)		1.50 mg/mL		
*Boswellia rivae* (frankincense)		*E. coli* (ATCC 8739)		3.00 mg/mL		
		*P. aeruginosa* (ATCC 27858)		1.00 mg/mL		
*Boswellia sacra* (frankincense) (2 samples)		*E. coli* (ATCC 25922)		4.00–6.00 mg/mL	*α*-Pinene (18.30–28.00%), *α*-thujene (3.90–11.20%), limonene (11.20–13.10%)	[116]
*Boswellia *spp. (frankincense) (4 samples)				6.00 mg/mL	*α*-Pinene (18.80–24.20%), limonene (11.70–19.00%)	
*Boswellia thurifera* (frankincense)					*α*-Pinene (28.00%), limonene (14.60%)	

*Cananga odorata* (ylang-ylang)	MIC	*P. aeruginosa* (ATCC 27858)	Acetone	3.00 mg/mL	Bicyclosesquiphellandrene (19.50%), *β*-farnesene (13.90%)	[99]
*Cananga odorata* (ylang-ylang), heads				1.50 mg/mL	Benzyl acetate (31.90%), linalool (27.00%), methyl benzoate (10.40%)	

*Canarium luzonicum* (elemi)	MIC	*P. aeruginosa* (ATCC 27858)	Acetone	2.00 mg/mL	Limonene (41.90%), elemol (21.60%), *α*-phellandrene (11.40%)	[99]

*Carum carvi* (caraway)	MIC	*P. aeruginosa* (ATCC 27858)	Acetone	2.00 mg/mL	Limonene (27.60%), carvone (67.50%)	[99]
		*P. aeruginosa*	DMSO	>16.00 *μ*g/mL	DL-Limonene (53.35%), *β*-selinene (11.08%), *β*-elemene (10.09%)	[118]

*Caryophyllus aromaticus* (clove)	ADM_90_	*P. aeruginosa* (ATCC 27853 and 15 clinical isolates)	Tween 80	3.00 mg/mL	Eugenol (75.85%), eugenyl acetate (16.38%)	[119]
		*E. coli* (ATCC 25922 and 15 clinical isolates)				

*Cinnamomum cassia* (cinnamon)	MIC	*P. aeruginosa*	DMSO	≤1.00 *μ*g/mL	*trans*-Caryophyllene (17.18%), eugenol (14.67%), linalool L (14.52%), *trans*-cinnamyl acetate, (13.85%), cymol (11.79%), cinnamaldehyde (11.25%)	[118]

*Cinnamomum zeylanicum* (cinnamon)	MAC	*E. coli* (ATCC 25922)	75% ethanol	200.00 mg/L	NCR	[231]
		*E. coli* (10 clinical strains)		400.00 mg/L		
	ADM	*E. coli*	DMSO	1.25 *μ*L/mL	NCR	[232]
		*E. coli* (ATCC 25922)	10% DMSO	>1.6 mg/mL	Cinnamaldehyde (52.42%)	[80]
	MIC	*P. aeruginosa* (ATCC 27853)	75% ethanol	400.00 mg/L	NCR	[231]
		*P. aeruginosa* (clinical strain)				
	ADM	*P. aeruginosa* (ATCC 27853)	10% DMSO	>0.80 mg/mL	Cinnamaldehyde (52.42%)	[80]
	MIC		Acetone	1.50 mg/mL	Eugenol (80.00%)	[99]
	ADM_90_	*P. aeruginosa* (ATCC 27853 and 15 clinical isolates)	Tween 80	0.80 mg/mL	Cinnamaldehyde (86.31%)	[119]
		*E. coli* (ATCC 25922 and 15 clinical isolates)		0.25 mg/mL		

*Citrus aurantifolia* (lime)	ADM	*E. coli* (ATCC 25922)	10% DMSO	6.40 mg/mL	NCR	[80]
		*P. aeruginosa* (ATCC 27853)				

*Citrus aurantium* (bitter orange), flowers	MIC	*E. coli* (ATCC 25922)	10% DMSO	1.25 mg/mL	Limonene (27.50%), *α*-terpineol (14.00%), *E*-nerolidol (17.50%), *α*-terpinyl acetate (11.7%)	[120]
		*E. coli* (ATCC 8739)				
		*P. aeruginosa* (ATCC 9027)		2.50 mg/mL		

*Citrus aurantium* (petitgrain)	MIC	*P. aeruginosa* (ATCC 27858)	Acetone	2.00 mg/mL	Linalyl acetate (54.90%), linalool (21.10%)	[99]

*Citrus bergamia* (bergamot)	MAC	*E. coli* (ATCC 8739)	n.m.	5.0 *μ*L/mL	Bergamol (16.10%), linalool (14.02%), D-limonene (13.76%)	[62]

*Citrus grandis* (grapefruit)	MIC	*P. aeruginosa* (ATCC 27858)	Acetone	1.50 mg/mL	Limonene (74.80%)	[99]
	ADM	*E. coli*	DMSO	2.5 *μ*L/mL	NCR	[232]

*Citrus medica limonum* (lemon)	ADM	*E. coli* (ATCC 25922)	10% DMSO	>6.4 mg/mL	NCR	[80]
		*P. aeruginosa* (ATCC 27853)		12.80 mg/mL		
	MIC	*P. aeruginosa* (ATCC 27858)	Acetone	2.00 mg/mL		[99]

*Citrus sinensis* (orange)	ADM	*E. coli* (ATCC 25922)	10% DMSO	>12.8 mg/mL	NCR	[80]
	MAC	*E. coli* (ATCC 10536)	0.1% ethanol	1.875 mg/L		[121]
	ADM	*P. aeruginosa* (ATCC 27853)	10% DMSO	>12.80 mg/mL		[80]
	MAC	*P. aeruginosa* (ATCC 15442)	0.1% ethanol	1.88 mg/mL		[121]
	MIC	*P. aeruginosa* (ATCC 27858)	Acetone	2.00 mg/mL	Limonene (93.20%)	[99]

*Commiphora guidotti* (myrrh)	MIC	*E. coli* (ATCC 8739)	Acetone	4.00 mg/mL	(*E*)-*β*-Ocimene (52.60%), *α*-santalene (11.10%), (*E*)-*α*-bisabolene (16.00%)	[117]
		*P. aeruginosa* (ATCC 27858)		1.40 mg/mL		
*Commiphora myrrha* (myrrh)		*E. coli* (ATCC 8739)		1.00 mg/mL	Furanogermacrene (15.9%), furanoeudesma-1,3-diene (44.3%)	
		*P. aeruginosa* (ATCC 27858)		0.50 mg/mL		
		*P. aeruginosa* (ATCC 27858)		4.00 mg/mL	Furanoeudesma-1,3-diene (57.70%), lindestrene (16.30%)	[99]

*Coriandrum sativum* (coriander), seed	MIC	*E. coli* (7 clinical isolates)	0.5% DMSO with Tween 80	0.14 mg/mL	NCR	[122]
*Cumin cymene* (cumin) (6 samples)				0.30 mg/mL		

*Cupressus arizonica* (smooth cypress)	MIC	*P. aeruginosa* (ATCC 27853)	10% DMSO	6.02–23.60 *μ*g/mL	*α*-Pinene (20.00–60.50%), *δ*-3-carene (1.00–15.60%), umbellulone (0.80–18.40%)	[123]
*Cupressus arizonica* (smooth cypress), branches		*E. coli* (ATCC 25922)		0.37 *μ*g/mL	*α*-Pinene (58.60%), *δ*-3-carene (15.60%)	
		*P. aeruginosa* (ATCC 27853)		11.80 *μ*g/mL		
*Cupressus arizonica* (smooth cypress), female cones		*E. coli* (ATCC 25922)		2.95 *μ*g/mL	*α*-Pinene (60.50%), *δ*-3-carene (15.30%)	
		*P. aeruginosa* (ATCC 27853)		6.02 *μ*g/mL		
*Cupressus arizonica* (smooth cypress), leaves		*E. coli* (ATCC 25922)		0.38 *μ*g/mL	*α*-Pinene (20.00%), umbellulone (18.40%)	
		*P. aeruginosa* (ATCC 27853)		23.60 *μ*g/mL		
*Cupressus sempervirens* (cypress)		*P. aeruginosa* (ATCC 27853)	Acetone	2.00 mg/mL	*α*-Pinene (41.20%), *δ*-3-carene (23.70%)	[99]

*Cymbopogon giganteus* (lemongrass)	MIC	*E. coli* (CIP 105182)	0.5% ethanol	6.3 mg/mL	Limonene (42.00%), *trans-p*-mentha-1(7),8-dien-2-ol (14.20%), *cis*-*p*-mentha-1(7),8-dien-2-ol (12.00%)	[124]
		*P. aeruginosa* (CRBIP 19.249)		70.00 mg/mL		

*Cymbopogon citratus* (lemongrass)	MIC	*E. coli* (CIP 105182)	0.5% ethanol	10 mg/mL	Geranial (48.1%), neral (34.6%), myrcene (11.0%)	[124]
	MAC	*E. coli* (clinical isolate VR 12 and MTCC 424)	Sodium taurocholate	1.66–3.33 *μ*L/mL	Citral (72.80%)	[125, 126]
		*P. aeruginosa* (MTCC 424 and clinical isolate VR 6)		11.60–>20.00 *μ*L/mL		
	MIC	*P. aeruginosa* (CRBIP 19.249)	0.5% ethanol	>80.00 mg/mL	Geranial (48.10%), neral (34.60%), myrcene (11.00%)	[124]
		*P. aeruginosa* (ATCC 27858)	Acetone	1.50 mg/mL	Geranial (44.80%)	[99]
		*P. aeruginosa*	DMSO	>16.00 *μ*g/mL	Geranial (47.34%), *β*-myrcene (16.53%), Z-citral (8.36%)	[118]

*Cymbopogon martinii* (palmarosa)	MAC	*E. coli* (clinical isolate VR 12 and MTCC 424)	sodium taurocholate	1.66–4.16 *μ*L/mL	Geraniol (61.60%)	[125, 126]
		*P. aeruginosa* (MTCC 424 and clinical isolate VR 6)		8.33–>20.00 *μ*L/mL		

*Cymbopogon nardus* (citronella)	MIC	*P. aeruginosa* (ATCC 27858)	acetone	1.50 mg/mL	Citronellal (38.30%), geraniol (20.70%), citronellol (18.80%)	[99]
*Daucus carota* (carrot seed)				3.00 mg/mL	Carotol (44.40%)	

*Eucalyptus camaldulensis* (eucalyptus)	ADM	*E. coli*	DMSO	5.00 *μ*L/mL	NCR	[232]
	MIC	*E. coli* (ATCC 25922)	Acetone	2.00 mg/mL		[130]
		*P. aeruginosa* (ATCC 27853)		1.00 mg/mL		

*Eucalyptus globulus* (eucalyptus)	ADM	*E. coli* (ATCC3428)	Tween 20	51.36 *μ*g/mL	Eucalyptol (47.20%), (+)-spathulenol (18.10%)	[81]
	MIC	*E. coli* (ATCC 25922)	Tween 80	10.00 mg/mL	1,8-Cineol (81.93%)	[128]
		*P. aeruginosa* (ATCC 27853)		10.00 mg/mL		[128]
	MAC	*E. coli* (clinical isolate and MTCC 424)	Sodium taurocholate	1.66–3.33 *μ*L/mL	Cineole (23.20%)	[125, 126]
		*P. aeruginosa* (MTCC 424 and clinical isolate VR 6)		8.33–>20.00 *μ*L/mL		
	MIC	*P. aeruginosa* (ATCC 9027)	DMSO	0.10% v/v	NCR	[129]
		*P. aeruginosa* (ATCC 27858)	Acetone	3.00 mg/mL	1,8-Cineole (58.00%), *α*-terpineol (13.20%)	[99]
		*E. coli* (ATCC 25922)		2.00 mg/mL	NCR	[130]
		*P. aeruginosa* (ATCC 27853)		1.00 mg/mL		
*Eucalyptus radiata* (eucalyptus)		*E. coli* (ATCC 25922)	Acetone	2.00 mg/mL	1,8-Cineole (65.7% ± 9.5), *α*-terpineol (12.8% ± 4.4)	[130]
		*P. aeruginosa* (ATCC 27853)		1.00 mg/mL		[130]
*Eucalyptus citriodora* (eucalyptus)		*E. coli* (ATCC 25922)		2.00 mg/mL	NCR	[130]
		*P. aeruginosa* (ATCC 27853)		1.00 mg/mL		[130]
*Eucalyptus smithii* (eucalyptus)		*E. coli* (ATCC 25922)		2.00 mg/mL	NCR	[130]
		*P. aeruginosa* (ATCC 27853)		2.00 mg/mL		[130]
*Eucalyptus dives* (eucalyptus)		*E. coli* (ATCC 25922)		2.00 mg/mL	NCR	[130]
		*P. aeruginosa* (ATCC 27853)		1.00 mg/mL		[130]
*Eucalyptus intertexta* (eucalyptus)		*E.coli* (ATCC 10536)	10% DSMO	15.6 *μ*g/mL	NCR	[131]
		*P. aeruginosa* (ATCC 27853)		NI		
*Eucalyptus largiflorens* (eucalyptus)		*E.coli* (ATCC 10536)				
		*P. aeruginosa* (ATCC 27853)				

*Foeniculum dulce* (fennel)	MIC	*P. aeruginosa* (ATCC 27858)	Acetone	3.00 mg/mL	*E*-Anethole (79.10%)	[99]

*Foeniculum vulgare* (fennel)	MAC	*E. coli* (ATCC 25922)	DMSO	0.25 mg/mL	*trans*-Anethole (68.53%), estragole (10.42%)	[132]
		*P. aeruginosa* (ATCC 9027)		>10.00 mg/mL		
	MIC	*P. aeruginosa*		>16.00 *μ*g/mL	*trans*-Anethole (33.3%), DL-limonene (19.66%), carvone (12.03%)	[118]
*Foeniculum vulgare* (fennel) (6 samples)	MIC	*P. aeruginosa* (ATCC 27853)		1000.00–>2000.00 *μ*g/mL	Fenchone (16.90–34.70%), estragole (2.50–66.00%), *trans*-anethole (7.90–77.70%)	[133]
		*E. coli* (ATCC 25922)		62.50–500 *μ*g/mL		

*Foeniculum vulgare* Mill. ssp. *vulgare* (fennel), Aurelio	MAC	*P. aeruginosa* (ATCC 27853)	Tween 20	>100.00 *μ*g/mL	Limonene (16.50–21.50%), (*E*)-anethole (59.80–66.00%)	[134]
		*E. coli* (ATCC 25922)		50.00 *μ*g/mL		
*Foeniculum vulgare* Mill. ssp. *vulgare* (fennel), Spartaco		*E. coli* (ATCC 25922)		50.00–100.00 *μ*g/mL	Limonene (0.20–17.70%), (*E*)-anethole (66.30–90.40%)	
		*P. aeruginosa* (ATCC 27853)		≥100.00 *μ*g/mL		

*Geranium dissectum* (geranium)	MAC	*P. aeruginosa*	DMSO	>16.00 *μ*g/mL	*β*-Citronellol (25.45%), geraniol (13.83%)	[118]

*Hyssopus officinalis* (hyssop)	MAC	*P. aeruginosa* (ATCC 27858)	Acetone	2.00 mg/mL	Isopinocamphone (48.70%), pinocamphone (15.50%)	[99]

*Jasminum sambac* (jasmine)	MAC	*E. coli* (MTCC 443)	Sodium taurocholate	31.25 *μ*L/mL	Linalool (59.00%), benzyl acetate (22.50%)	[233]

*Juniperus excelsa* (juniper), berries, Ohrid	ADM	*E. coli* (25927)	50% DSMO	>50.00%	Sabinene (58.85%)	[87]
*Juniperus officinalis* (juniper), berry	MIC	*E. coli* (ATCC 25922)	Tween 80	20.00 mg/mL	*α*-Pinene (39.760%)	[128]
		*P. aeruginosa* (ATCC 27853)				
*Juniperus virginiana* (juniper)		*P. aeruginosa* (ATCC 27858)	Acetone	2.00 mg/mL	Thujopsene (29.80%), cedrol (14.90%), *α*-cedrene (12.40%)	[99]
*Juniperus virginiana* (juniper), berries					*α*-Pinene (20.50%), myrcene (13.70%), bicyclosesquiphellandrene (10.70%)	

*Kunzea ericoides* (Kānuka)	MAC	*E. coli* (ATCC 11229)	Tween 80	>2.00% v/v	*α*-pinene (61.60%)	[137]
		*P. aeruginosa* (ATCC 15442)		>2.00% v/v		
	MIC	*P. aeruginosa* (ATCC 9027)	Acetone	4.00 mg/mL	*α*-Pinene (26.20–46.70%), *p*-cymene (5.80–19.10%)	[138]

*Laurus nobilis* (bay)	MIC	*P. aeruginosa* (ATCC 27858)	Acetone	2.67 mg/mL	Eugenol (57.20%), myrcene (14.30%), chavicol (12.70%)	[99]

*Lavandula angustifolia* (lavender)	MIC	*E. coli* (ATCC 8739)	10% DSMO	730.1 *μ*g/mL	Linalool (25.10%), linalyl acetate (22.50%)	[139]
		*E. coli* (ampicillin-resistant NCTC 10418)		722.2 *μ*g/mL		
		*P. aeruginosa* (NCTC 1662)		1040.00 *μ*g/mL		
		*E. coli* (ATCC 11775)	Acetone	6.20 mg/mL	Linalool (30.80%), linalyl acetate (31.30%)	[140]
		*P. aeruginosa* (ATCC 9027)		8.60 mg/mL		
		*P. aeruginosa* (ATCC 27858)		2.00 mg/mL	Linalyl acetate (36.70%), linalool (31.40%), terpinen-4-ol (14.90%)	[99]
*Lavandula dentata* (French lavender)		*E. coli* (BNI 2)	5% DMSO	2.20 mg/mL	Camphor (12.40%)	[141]
*Lavandula officinalis* (lavender)		*P. aeruginosa*	DMSO	>16.00 *μ*g/mL	*δ*-3-Carene (17.14%), *α*-fenchene (16.79%), diethyl phthalate (13.84%)	[118]
*Lavandula stoechas* (French lavender)		*E. coli* (STCC 471)	Tween 80	8.00 *μ*L/mL	10s,11s-Himachala-3(12),4-diene (23.62%), cubenol (16.19%)	[142]
*Lavandula stoechas* (French lavender), flower		*E. coli* (NRRL B-3008)	20% DMSO	250.00 *μ*g/mL	*α*-Fenchone (39.20%)	[47]
		*P. aeruginosa* (NRRL B-23)				
*Lavandula stoechas* (French lavender), leaf		*E. coli* (NRRL B-3008)		250.00 *μ*g/mL	*α*-Fenchone (41.90%), 1,8-cineole (15.60%), camphor (12.10%)	
		*P. aeruginosa* (NRRL B-23)		500.00 *μ*g/mL		

*Leptospermum scoparium* (manuka)	MAC	*E. coli* (ATCC 11229)	Tween 80	>2.00% v/v	(−)-*(E)*-Calamenene (14.50%), leptospermone (17.60%)	[137]
		*P. aeruginosa* (ATCC 15442)		0.85% v/v		
	MIC	*P. aeruginosa* (ATCC 9027)	Acetone	4.00 mg/mL	Eudesma-4(14),11-diene (6.20–14.50%), *α*-selinene (5.90–13.50%), (*E*)-methyl cinnamate (9.20–19.50%)	[138]

*Litsea cubeba* (May Chang)	MIC	*P. aeruginosa* (ATCC 27858)	Acetone	1.50 mg/mL	Geranial (45.60%), nerol (31.20%)	[99]
*Matricaria chamomilla* (German chamomile)				4.00 mg/mL	Bisabolene oxide A (46.90%), *β*-farnesene (19.20%)	[99]

*Matricaria recutita* (German chamomile)	ADM_90_	*P. aeruginosa* (ATCC 27853 and 15 clinical isolates)	Tween 80	54.40 mg/mL	Chamazulene (31.48%), *α*-bisabolol (15.71%), bisabolol oxide (15.71%)	[119]
		*E. coli* (ATCC 25922 and 15 clinical isolates)				

*Matricaria songarica* (chamomile)	MIC	*E. coli* (CCTCC AB91112)	Tween 80	100 *μ*g/mL	*E*-*β*-Farnesene (10.58%), bisabolol oxide A (10.46%)	[143]
		*P. aeruginosa* (CCTCC AB93066)		200.00 *μ*g/mL		

*Melaleuca alternifolia* (tea tree)	MIC	*E. coli* (ATCC 10536)	Tween 80	0.12% v/v	NCR	[147]
		*E. coli* (110 clinical isolates)		0.12–0.25% v/v		
		*E. coli* (AG100)	n.m.	0.25% (v/v)	Terpinen-4-ol (39.80%), *γ*-terpinene (17.80%)	[149, 150]
		*E. coli* (ATCC 25922)	None used	0.25% (v/v)	Terpinen-4-ol (40.00%), *γ*-terpinen (13.00%), *p*-cymene (13.00%)	[97]
		*E. coli* (ATCC 11775)	Acetone	3.70 mg/mL	Terpinen-4-ol (38.60%), *γ*-terpinene (21.60%)	[140]
	MAC	*E. coli* (ATCC 11229)	Tween 80	0.25% v/v	*α*-Terpinene (11.40%), *γ*-terpinene (22.50%), terpinen-4-ol (35.20%)	[137]
		*P. aeruginosa* (ATCC 15442)		1.00% v/v		
	MIC	*P. aeruginosa* (NCTC 6749)	n.m.	8.00% (v/v)	Terpinen-4-ol (39.80%), *γ*-terpinene (17.80%)	[150]
		*P. aeruginosa* (10 clinical isolates)	Tween 80	2.00–5.00% v/v	terpinen-4-ol (35.70%)	[152]
	ADM	*P. aeruginosa* (NCTC 6750)	0.5% Tween 20	>2.00% v/v	Terpinen-4-ol (42.80%), *γ*-terpinene (18.20%)	[115]
		*E. coli* (NCTC 8196)		0.20% v/v		[115]
	MIC	*P. aeruginosa* (NCIB 8295)	n.m.	12.50% v/v	NCR	[145]
	MIC_90_	*P. aeruginosa* (30 clinical isolates)	Tween 80	4.00%	Terpinen-4-ol (40.30%), *γ*-terpinene (19.70%)	[234]
	MIC	*P. aeruginosa* (ATCC 9027)	Acetone	8.60 mg/mL	Terpinen-4-ol (38.60%), *γ*-terpinene (21.60%)	[140]
	MIC	*P. aeruginosa* (ATCC 27858)	Acetone	2.00 mg/mL	Terpinen-4-ol (49.30%), *γ*-terpinene (16.90%)	[99]

*Melaleuca cajuputi* (cajuput)	MIC	*E. coli* (ATCC 25922)	Tween 80	5.00 mg/mL	1,8-Cineol (67.60%)	[128]
		*P. aeruginosa* (ATCC 27853)				
		*P. aeruginosa* (ATCC 15442)		1.90% v/v	1,8-Cineole (55.50%)	[137]
		*E. coli* (ATCC 11229)		0.40% v/v		

*Melaleuca quinquenervia* (niaouli)	MIC	*E. coli* (ATCC 11229)	Tween 80	0.40% v/v	1,8-Cineole (61.20%)	[137]
		*P. aeruginosa* (ATCC 15442)		1.90%v/v		
*Melaleuca viridiflora* (niaouli)		*P. aeruginosa* (ATCC 27858)	Acetone	2.00 mg/mL	1,8-Cineole (45.90%), *α*-terpinene (21.00%)	[99]

*Melissa officinalis* (lemon balm)	MIC	*P. aeruginosa* (NCTC 1662)	10% DSMO	1000.30 *μ*g/mL	Citronellal (20.50%), *β*-citronellol (11.50%), geraniol (17.00%)	[139]
		*E. coli* (ATCC 8739)		442.30 *μ*g/mL		
		*E. coli* (ampicillin-resistant NCTC 10418)		567.40 *μ*g/mL		
		*E. coli* (ATCC 25922)	n.m.	10.00 mg/mL	NCR	[85]

*Mentha piperita* (peppermint)	MIC	*E. coli* (ATCC 25922)	Tween 80	0.6 mg/mL	1,8-Cineol (12.06%), menthone (22.24%), menthol (47.29%)	[128]
	MAC	*E. coli* (clinical isolate and MTCC 424)	Sodium taurocholate	1.66–2.50 *μ*L/mL	Menthol (36.40%)	[125, 126]
	MIC	*E. coli* (ATCC 11775)	Acetone	5.70 mg/mL	Menthone (18.20%), menthol (42.90%)	[140]
		*E. coli* (ATCC 25922)	DMSO	1.25–2.50 mg/mL	Menthol (27.50–42.30%), menthone (18.40–27.90%)	[155]
			n.m.	3.20 mg/mL	NCR	[85]
		*P. aeruginosa* (ATCC 27853)	Tween 80	20.00 mg/mL	1,8-Cineol (12.06%), menthone (22.24%), menthol (47.29%)	[128]
		*P. aeruginosa*	DMSO	≤1.00 *μ*g/mL	Menthone (40.82%), carvone (24.16%)	[118]
		*P. aeruginosa* (ATCC 9027)		0.10% v/v	NCR	[129]
	MAC	*P. aeruginosa* (MTCC 424 and clinical isolate VR 6)	Sodium taurocholate	10.00–>20.00 *μ*L/mL	Menthol (36.40%)	[125, 126]
	MIC	*P. aeruginosa* (ATCC 9027)	Acetone	8.60 mg/mL	Menthone (18.20%), menthol (42.90%)	[140]
		*P. aeruginosa* (ATCC 27853)	DMSO	2.50–5.00 mg/mL	Menthol (27.50–42.30%), menthone (18.40–27.90%)	[155]
		*P. aeruginosa* (ATCC 27858)	Acetone	2.00 mg/mL	Menthol (47.50%), menthone (18.60%)	[99]
		*E. coli* (CIP 105182)	Ethanol	40.0 mg/mL	Menthol (39.30%), menthone (25.20%)	[156]
		*P. aeruginosa* (CRBIP 19.249)	0.5% ethanol	>80.00 mg/mL		

*Myrtus communis* (myrtle)	MIC	*P. aeruginosa* (ATCC 27858)	Acetone	2.00 mg/mL	Myrtenyl acetate (28.20%), 1,8-cineole (25.60%), *α*-pinene (12.50%)	[99]
	ADM	*E. coli* (ATCC 35218)	Tween 20	11.20 mg/mL	NCR	[157]

*Ocimum basilicum* (basil)	MIC	*E. coli* (CIP 105182)	Ethanol	8.30 mg/mL	Linalool (57.00%), eugenol (19.20%)	[156]
	MAC	*E. coli *(ATCC 8739)	n.m.	1.25 *μ*L/mL	Eugenol (62.60%), caryophyllene (21.51%)	[62]
	ADM	*E. coli* (ATCC 25922)	96% ethanol	8.00 *μ*L/mL	Estragole (86.4%)	[223]
		*E. coli* (ESBL+) (4 clinical strains from wounds)		8.50–9.25 *μ*L/mL		
		*E. coli* (ESBL−) (4 clinical strains from wounds)		10.00–11.50 *μ*L/mL		
	MIC	*P. aeruginosa* (CRBIP 19.249)	0.5% ethanol	>80.00 mg/mL	Linalool (57.00%), eugenol (19.20%)	[156]
	MAC	*P. aeruginosa* (ATCC 9027)	Tween 80	0.0030% v/v	Linalool (54.95%), methyl chavicol (11.98%)	[158]
		*P. aeruginosa* (clinical isolate)				
	MIC	*P. aeruginosa* (ATCC 27858)	Acetone	2.67 mg/mL	Linalool (54.10%)	[99]
	MIC_90_	*E. coli* (ATCC 8739)	n.m.	160.00 *μ*g/mL	Methyl chavicol (46.90%), geranial (19.10%), neral (15.15%)	[159]
	MIC	*E. coli* (ATCC 35210)	Tween 80	0.18–5.40 *μ*g/mL	Linalool (30.30–58.60%)	[160]
		*P. aeruginosa* (ATCC 27853)		0.11–11.74 *μ*g/mL		

*Origanum acutidens* (Turkey oregano)	MIC	*E. coli* (A1)	10% DMSO	62.50 *μ*g/mL	Carvacrol (72.00%)	[161]
		*P. aeruginosa* (ATCC9027)		125.00 *μ*g/mL		
		*P. aeruginosa* (ATCC27859)		125.00 *μ*g/mL		
*Origanum majorana* (marjoram)		*P. aeruginosa* (ATCC 9027)	DMSO	0.05% v/v	NCR	[129]
		*P. aeruginosa* (ATCC 27858)	Acetone	2.00 mg/mL	1,8-Cineole (46.00%), linalool (26.10%)	[99]
*Origanum microphyllum* (oregano)		*P. aeruginosa* (ATCC 227853)	Tween 80	NI	Terpin-4-ol (24.86%), *γ*-terpinene (13.83%), linalool (10.81%)	[162]
		*E. coli* (ATCC 25922)		3.35 mg/mL		
*Origanum scabrum* (oregano)		*E. coli* (ATCC 25922)		0.28 mg/mL	Carvacrol (74.86%)	
		*P. aeruginosa* (ATCC 227853)		1.27 mg/mL		

*Origanum vulgare* (oregano)	MAC	*E. coli* (ATCC 25922)	75% ethanol	200.00 mg/L	NCR	[231]
		*E. coli* (10 clinical isolates)		200.00–400.00 mg/L		
		*E. coli* (ATCC 8739)	n.m.	0.63 *μ*L/mL	Carvacrol (30.17%), *p*-cymene (15.20%), *γ*-terpinen (12.44%)	[62]
	ADM	*E. coli* (ATCC 35218)	Tween 20	0.70 mg/mL	NCR	[157]
	MIC	*P. aeruginosa* (ATCC 27853)	75% ethanol	800.00 mg/L		[231]
		*P. aeruginosa* (clinical isolate)		400.00 mg/L		
		*P. aeruginosa* (ATCC 2730)	n.m.	1648.00 mg/L		[164]
		*P. aeruginosa* (ATCC 9027)	DMSO	0.20% v/v		[129]
*Origanum vulgare* subsp. *hirtum* (Greek oregano)	MIC	*E. coli* (ATCC 25922)	10% DMSO and Tween 80	>512.00 *μ*g/mL	Linalool (96.31%)	[165]
		*P. aeruginosa* (ATCC 27853)				
*Origanum vulgare* subsp. *vulgare* (oregano)		*E. coli* (ATCC 25922)		213.30 *μ*g/mL	Thymol (58.31%), carvacrol (16.11%), *p*-cymene (13.45%)	
		*P. aeruginosa* (ATCC 27853)		256.00 *μ*g/mL		

*Pelargonium graveolens* (geranium)	ADM	*E. coli* (ATCC 25922)	10% DMSO	>6.40 mg/mL	NCR	[80]
		*E. coli* (ATCC 35218)	Tween 20	5.60 mg/mL		[157]
	MIC	*E. coli* (clinical strains isolated from wounds)	Ethanol	3.00–3.75 mL/mL	Citronellol (26.70%), geraniol (13.40%)	[166]
	ADM	*P. aeruginosa* (ATCC 27853)	10% DMSO	>12.80 mg/mL	NCR	[80]
	MIC	*P. aeruginosa*	Ethanol	9.25–10.50 mL/mL.	Citronellol (26.70%), geraniol (13.40%)	[166]

*Pelargonium odoratissimum* (geranium)	MIC	*P. aeruginosa* (ATCC 27858)	Acetone	2.00 mg/mL	Citronellol (34.20%), geraniol (15.70%)	[99]

*Perovskia abrotanoides* (Russian sage)	MIC	*E. coli* (ATCC 8739)	10% DMSO	>8.00 *μ*L/mL	Camphor (23.00%), 1,8-cineole (22.00%), *α*-pinene (12.00%)	[167]
		*P. aeruginosa* (ATCC 9027)				

*Pimpinella anisum* (anise)	MIC	*E. coli*	DMSO	>500.00 *μ*g/mL	NCR	[168]
		*P. aeruginosa*				
		*P. aeruginosa*		>16.00 *μ*g/mL	Anethole (64.82%)	[118]

*Pinus sylvestris* (pine)	MIC	*P. aeruginosa* (ATCC 27858)	Acetone	2.00 mg/mL	Bornyl acetate (42.30%), camphene (11.80%), *α*-pinene (11.00%)	[99]
*Piper nigrum* (black pepper)		*P. aeruginosa* (ATCC 27858)	Acetone	2.00 mg/mL	*β*-Caryophyllene (33.80%), limonene (16.40%)	[99]

*Pogostemon cablin* (patchouli)	MIC	*E. coli* (ampicillin-resistant NCTC 10418)	10% DSMO	530.2 *μ*g/mL	*α*-Guaiene (13.80%), *α*-bulnesene (17.10%), patchouli alcohol (22.70%)	[139]
		*E. coli* (ATCC 8739)		410.7 *μ*g/mL		
		*P. aeruginosa* (NCTC 1662)		1200.00 *μ*g/mL		
*Pogostemon patchouli* (patchouli)		*P. aeruginosa* (ATCC 27858)	Acetone	2.00 mg/mL	Patchouli alcohol (37.30%), *α*-bulnesene (14.60%), *α*-guaiene (12.50%)	[99]

*Rosmarinus officinalis* (rosemary)	MIC	*E. coli* (ATCC 8739)	Tween 80	0.25% v/v	1,8-Cineole (27.23%), *α*-pinene (19.43%), camphor (14.26%), camphene (11.52%)	[169]
		*E. coli* (ATCC 8739)	10% DSMO	733.70 *μ*g/mL	1,8-Cineol (29.20%), (+)-camphor (17.20%)	[139]
		*E. coli* (ampicillin-resistant NCTC 10418)		810.70 *μ*g/mL		
		*E. coli* (ATCC 8739)	Tween 80	0.03% (v/v)	1,8-Cineole (26.54%), *α*-pinene (20.14%), camphene (11.38%), camphor (12.88%)	[170]
		*E. coli* (MTCC 723)	n.m.	>11.00 mg/mL	NCR	[171]
		*E. coli* (ATCC 8739)	Hexane	0.47–3.75 mg/mL	*α*-Pinene (8.14–11.47)%, 1,8-cineole (10.56–11.91%), camphor (16.57–16.89%), verbenone (17.43–23.79%), bornyl acetate (9.19–11.62%)	[171]
	ADM	*E. coli* (ATCC 25922)	10% DMSO	>6.40 mg/mL	NCR	[80]
		*E. coli* (ATCC 35218)	Tween 20	11.20 mg/mL		[157]
	MIC	*E. coli* (ATCC 11775)	Acetone	4.50 mg/mL	1,8-Cineole (41.40%), *α*-pinene (13.30%), camphor (12.40%)	[140]
	ADM	*E. coli* (ATCC 25922)	96% ethanol	18.50 *μ*L/mL	1,8-Cineole (46.40%), camphor (11.40%), *α*-pinene (11.00%)	[223]
		*E. coli* (ESBL+) (4 clinical strains from wounds)		18.50–19.25 *μ*L/mL		
		*E. coli* (ESBL−) (4 clinical strains from wounds)		18.25 *μ*L/mL–20.0 *μ*L/mL		
	MIC	*P. aeruginosa* (ATCC 27853)	Tween 80	1.00%v/v	1,8-Cineole (27.23%), *α*-pinene (19.43%), camphor (14.26%), camphene (11.52%)	[169]
		*P. aeruginosa* (NCTC 1662)	10% DSMO	1113.30 *μ*g/mL	1,8-Cineol (29.20%), (+)-camphor (17.20%)	[139]
		*P. aeruginosa* (ATCC 27853)	Tween 80	0.10% v/v	1,8-Cineole (26.54%), *α*-pinene (20.14%), camphene (11.38%), camphor (12.88%)	[170]
		*P. aeruginosa* (MTCC 741)	n.m.	>11.00 mg/mL	NCR	[171]
		*P. aeruginosa* (ATCC 9027)	DMSO	0.20% v/v		[129]
	ADM	*P. aeruginosa* (ATCC 27853)	10% DMSO	>6.40 mg/mL	NCR	[80]
	MIC	*P. aeruginosa* (ATCC 9027)	Acetone	6.20 mg/mL	1,8-Cineole (41.40%), *α*-pinene (13.30%), camphor (12.40%)	[140]
		*P. aeruginosa* (ATCC 27858)		2.00 mg/mL	1,8-Cineole (48.00%)	[99]
	ADM_90_	*P. aeruginosa* (ATCC 27853 and 15 clinical isolates)	Tween 80	79.91 mg/mL	Camphor (27.51%), limonene (21.01%), myrcene (11.19%), *α*-pinene (10.37%)	[119]
		*E. coli* (ATCC 25922 and 15 clinical isolates)		79.35 mg/mL		

*Salvia bracteata* (sage)	MIC	*E. coli* (ATCC 25922)	Tween 20	>100.00 *μ*g/mL	Caryophyllene oxide (16.60%)	[173]
	MAC	*P. aeruginosa* (ATCC 27853)				

*Salvia eremophila* (sage)	MIC	*E. coli* (ATCC 10536)	10% DMSO	500.00 *μ*g/mL	Borneol (21.83%), *α*-pinene (18.80%), bornyl acetate (18.68%)	[174]
		*P. aeruginosa* (ATCC 27853)		NI		

*Salvia nilotica* (sage)	ADM	*E. coli* (ATCC 25922)	n.m.	NI	*trans*-Caryophyllene (10.90%)	[175]
		*P. aeruginosa* (ATCC 227853)		7.80 mg/mL		

*Salvia officinalis* (sage)	ADM	*E. coli* (ATCC 35218)	Tween 20	11.2 mg/mL	NCR	[157]
	MIC	*E. coli* (ATCC 8739)	10% DSMO	475.0 *μ*g/mL	1,8-Cineol (27.40%), *α*-thujone (16.30%), *β*-thujone (11.20%), borneol (10.40%)	[139]
		*E. coli* (ampicillin-resistant NCTC 10418)		548.0 *μ*g/mL		
		*P. aeruginosa* (NCTC 1662)		1250.30 *μ*g/mL		
		*P. aeruginosa* (ATCC 9027)	DMSO	0.20% v/v	NCR	[129]
	ADM	*P. aeruginosa* (clinical strain)	n.m.	7.50 mg/mL	NCR	[176]
		*E. coli* (ATCC 25922)		3.75 mg/mL		
		*E. coli* (clinical strain)		7.50 mg/mL		

*Salvia ringens* (sage)	MIC	*E. coli* (ATCC 25922)	n.m.	3.25 mg/mL	*α*-Pinene (12.85%), 1,8-cineole (46.42%)	[177]
		*P. aeruginosa* (ATCC 227853)		3.75 mg/mL		
*Salvia rosifolia* (sage) (3 samples)		*E. coli* (NRRL B 3008)	20% DMSO	250–1000 *μ*g/mL	*α*-Pinene (15.70–34.80%), 1,8-cineole (16.60–25.10%), *β*-pinene (6.70–13.50%)	[178]
		*P. aeruginosa* (NRRL B 23)		250.00–500.00 *μ*g/mL		
*Salvia rubifolia* (sage)		*E. coli* (ATCC 25922)	Tween 20	>100 *μ*g/mL	*γ*-Muurolene (11.80%).	[173]

*Salvia sclarea* (clary sage)	MIC	*P. aeruginosa* (ATCC 27858)	Acetone	3.50 mg/mL	Linalyl acetate (72.90%), linalool (11.90%)	[99]
*Santalum album* (sandalwood)				0.50 mg/mL	*α*-Santalol (32.10%)	
*Styrax benzoin* (benzoin)				3.00 mg/mL	Cinnamyl alcohol (44.80%), benzene propanol (21.70%)	

*Syzygium aromaticum* (clove)	MIC	*E. coli* (ATCC 8739)	Tween 80	0.50% v/v	Eugenol (68.52%), *β*-caryophyllene (19.00%), 2-methoxy-4-[2-propenyl]phenol acetate (10.15%)	[169]
	ADM	*E. coli* (ATCC 25922)	10% DMSO	>1.6 mg/mL	NCR	[80]
	MIC	*P. aeruginosa* (ATCC 27853)	Tween 80	0.13% v/v	Eugenol (68.52%), *β*-caryophyllene (19.00%), 2-methoxy-4-[2-propenyl]phenol acetate (10.15%)	[169]
	ADM	*P. aeruginosa* (ATCC 27853)	10% DMSO	>1.60 mg/mL	NCR	[80]
	MIC	*P. aeruginosa*	DMSO	>16.00 *μ*g/mL	Eugenol (84.07%), isoeugenol (10.39%)	[118]
		*P. aeruginosa* (ATCC 27858)	Acetone	1.50 mg/mL	Eugenol (82.20%), eugenol acetate (13.20%)	[99]

*Tagetes minuta* (Mexican marigold)	MIC_90_	*E. coli* (ATCC 8739)	n.m.	165.00 *μ*g/mL	Dihydrotagetone (33.90%), *E*-ocimene (19.90%), tagetone (16.10%)	[159]

*Tagetes patula* (French marigold)	MIC	*P. aeruginosa* (ATCC 27858)	Acetone	1.50 mg/mL	(*E*)-*β*-Ocimene (41.30%), *E*-tagetone (11.20%), verbenone (10.90%)	[99]

*Thymus capitatus* (thyme)	MIC	*E. coli* (ATCC 25922)	Tween 80	900.00 *μ*g/mL	*p*-Cymene (26.40%), thymol (29.30%), carvacrol (10.80%)	[181]
		*P. aeruginosa* (ATCC 27853)		>900.00 *μ*g/mL		
*Thymus capitatus* (thyme), commercial		*E. coli* (ATCC 25922)		900.00 *μ*g/mL	*α*-Pinene (25.20%), linalool (10.30%), thymol (46.10%)	
		*P. aeruginosa* (ATCC 27853)		>900.00 *μ*g/mL		
*Thymus herba-barona* (thyme), Gennargentu		*E. coli* (ATCC 25922)		450 *μ*g/mL	Thymol (46.90%), carvacrol (20.60%)	
		*P. aeruginosa* (ATCC 27853)		>900.00 *μ*g/mL		
*Thymus herba-barona* (thyme), Limbara		*E. coli* (ATCC 25922)		450 *μ*g/mL	*p*-Cymene (27.60%), thymol (50.30%)	
		*P. aeruginosa* (ATCC 27853)		>900.00 *μ*g/mL		

*Thymus hyemalis* (thymol, thymol/linalool, carvacrol chemotypes) (thyme)	MAC	*E. coli* (CECT 516)	95% ethanol	<0.2–2.0 *μ*L/mL	*p*-Cymene (16.00–19.80%), linalool (2.10–16.60%), thymol (2.90–43.00%), carvacrol (0.30–40.10%)	[61]

*Thymus numidicus*	ADM	*P. aeruginosa* (clinical strain)	n.m.	0.47 mg/mL	NCR	[176]
		*E. coli* (ATCC 25922)		0.12 mg/mL		
		*E. coli* (clinical strain)		0.23 mg/mL		

*Thymus schimperi* (thyme)	ADM	*E. coli*	DMSO	0.63 *μ*L/mL	NCR	[232]
*Thymus serpyllum* (thyme)		*E. coli* (ATCC 35218)	Tween 20	0.70 mg/mL		[157]

*Thymus vulgaris* (thyme)	MIC	*E. coli*	DMSO	62.50 *μ*g/mL	NCR	[168]
		*E. coli* (ATCC 8739)	10% DSMO	430.40 *μ*g/mL	*p*-Cymene (17.90%), thymol (52.40%)	[139]
		*E. coli* (ampicillin-resistant NCTC 10418)		360.60 *μ*g/mL		
		*E. coli* (ATCC 25922)	n.m.	0.30 mg/mL	NCR	[85]
	ADM	*E. coli* (ATCC 25922)	Ethanol	0.25 *μ*L/mL	Thymol (38.10%), *p*-cymene (29.10%)	[182]
		*E. coli* (2 multidrug-resistant clinical strains from groin)		0.25–0.50 *μ*L/mL		
		*E. coli* (7 multidrug-resistant clinical strains from wounds)				
		*E. coli* (multidrug-resistant clinical strain from abdominal cavity)		0.50 *μ*L/mL		
		*E. coli* (2 multidrug-resistant clinical strains from ulcers)		0.25–0.50 *μ*L/mL		
		*E. coli* (5 multidrug-resistant clinical strains from bedsores)				
	MIC	*E. coli* (ATCC 11775)	Acetone	0.50 mg/mL	Thymol (47.20%), *p-*cymene (22.10%)	[140]
	ADM	*E. coli* (ATCC3428)	Tween 20	9.25 *μ*g/mL	Thymol (48.10%), *p*-cymene (15.60%), *γ*-terpinene (15.40%)	[81]
	MIC	*P. aeruginosa*	DMSO	>500.00 *μ*g/mL	NCR	[168]
		*P. aeruginosa* (NCTC 1662)	10% DSMO	1250.30 *μ*g/mL	*p*-Cymene (17.90%), thymol (52.40%)	[139]
	ADM	*P. aeruginosa* (ATCC 27853)	Ethanol	0.50 *μ*L/mL	Thymol (38.10%), *p*-cymene (29.10%)	[182]
		*P. aeruginosa* (multidrug-resistant clinical strain from toes)		1.50 *μ*L/mL		
		*P. aeruginosa* (6 multidrug-resistant clinical strains from wounds)		1.50–2.00 *μ*L/mL		
		*P. aeruginosa* (6 multidrug-resistant clinical strains from ulcers)		2.00–2.50 *μ*L/mL		
		*P. aeruginosa* (6 multidrug-resistant clinical strains from bedsores)		1.50–2.00 *μ*L/mL		
	MIC	*P. aeruginosa* (ATCC 9027)	Acetone	8.6. mg/mL	Thymol (47.20%), *p-*cymene (22.10%)	[140]
		*P. aeruginosa* (ATCC 27858)		2.00 mg/mL	*p*-Cymene (39.90%), thymol (20.70%)	[99]

*Thymus vulgaris* (thyme) (thymol chemotype)	MAC	*E. coli* (CECT 516)	95% ethanol	0.5 *μ*L/mL	*p*-Cymene (18.70%), thymol (57.70%)	[61]
*Thymus zygis* subsp. *gracilis* (thyme) (thymol and two linalool chemotypes)				<0.2 *μ*L/mL	*p*-Cymene (0.50–11.20%), (*E*)-sabinene hydrate (0.20–18.20%), linalool (2.00–82.30%)	

*Vetiveria zizanioides/Andropogon muricatus* (vetiver)	MIC	*P. aeruginosa* (ATCC 27858)	Acetone	1.50 mg/mL	Zizanol (13.60%), *β*-vetirenene (7.20%)	[99]

^a^Scientific name (common name), part of plant (if applicable).

^b^MIC: microdilution method; MAC: macrodilution method; ADM: agar dilution method; CTA: contact time assay.

^c^American Type Culture Collection, Rockville, USA (ATCC); Colección Espanõla de Cultivos Tipo (CECT); collection of microorganisms of the Department of Microbiology (MFBF); culture collection of antibiotics-resistant microbes (CCRM); Eskişehir Osmangazi University, Faculty of Medicine, clinical isolate (OGU); Laboratorio de Microbiología, Facultad de Ciencias Médicas, Universidad Nacional de Cuyo, Mendoza, Argentina (LM); Microbial Type Culture Collection (MTCC); Mycology Laboratory (LM); National Center of Industrial Microorganisms (NCIM); National Collection of Type Cultures, London, Great Britain (NCTC); Spanish Collection of Type Cultures (STCC).

^d^DMSO concentration was not included; n.m.: not mentioned.

^e^NI: no inhibition.

^f^NCR: no composition results reported.

**Table 6:** Essential oil studies showing efficacy against other bacterial skin pathogens.

Essential oil^a^	Method^b^	Species strain^c^	Solvent^d^	Result^e^	Main components^f^	Reference
*Achillea millefolium* (yarrow)	MIC	*C. perfringens* KUKENS-Turkey	Tween 80	4.50 mg/mL	Eucalyptol (24.60%), camphor (16.70%), *α*-terpineol (10.20%)	[112]
*Achillea setacea* (bristly yarrow)				0.56 mg/mL	Sabinene (10.80%), eucalyptol (18.50%)	[113]
*Achillea teretifolia* (yarrow)				0.28 mg/mL	Eucalyptol (19.90%), camphor (11.10%), borneol (11.90%)	

*Eucalyptus globulus* (eucalyptus)	MIC	*S. pyogenes* (ATCC 12344)	Tween 80	10.00 mg/mL	1,8-Cineol (81.93%)	[128]
		*S. pyogenes* (NHLS 8668)		0.50 mg/mL		

*Eucalyptus radiata* (eucalyptus)	MIC	*S. pyogenes* (NHLS 8668)	Acetone	0.50–1.00 mg/mL	1,8-Cineole (65.7% ± 9.5), *α*-terpineol (12.8% ± 4.4)	[130]
*Eucalyptus camaldulensis* (eucalyptus)			Acetone	0.50 mg/mL		
*Eucalyptus citriodora* (eucalyptus)			Acetone	1.00 mg/mL		
*Eucalyptus smithii* (eucalyptus)			Acetone	0.50 mg/mL		
*Eucalyptus dives* (eucalyptus)			Acetone			

*Juniperus excelsa* (juniper), berries, Dojran	ADM	*S. pyogenes* (clinical isolate)	50% DSMO	>50.00%	*α*-Pinene (70.81%)	[87]
		*H. influenzae* (clinical isolate)		31.00 *μ*L/mL		
*Juniperus excelsa* (juniper), berries, Ohrid		*S. pyogenes* (clinical isolate)		>50.00%	Sabinene (58.85%)	
		*H. influenzae* (clinical isolate)				
*Juniperus excelsa* (juniper), leaves, Dojran		*H. influenzae* (clinical isolate)		>50.00%	*α*-Pinene (33.83%), cedrol (24.44%)	
*Juniperus excelsa* (juniper), leaves, Ohrid		*S. pyogenes* (clinical isolate)		125.00 *μ*L/mL	Sabinene (29.49%), *cis*-thujone (26.20%), menth-2-en-1-ol (12.86%)	
		*H. influenzae* (clinical isolate)				

*Juniperus officinalis* (juniper berry)	MIC	*S. pyogenes* (ATCC 12344)	Tween 80	20.00 mg/mL	*α*-Pinene (39.76%)	[128]

*Kunzea ericoides* (Kānuka)	MAC	*C. diphtheriae* (clinical isolate)	Tween 80	0.25% v/v	*α*-Pinene (61.60%)	[137]
		*C. minutissimus* (clinical isolate)		0.30% v/v		
	MIC	*S. pyogenes* (ATCC 8668)	Acetone	2.00 mg/mL	*α*-Pinene (26.2–46.7%), *p*-cymene (5.8–19.1%)	[138]
		*B. brevis* (ATCC 8246)		1.00 mg/mL		
		*B. agri* (ATCC 51663)				
		*B. laterosporus* (ATCC 64)				

*Leptospermum scoparium* (manuka)	MAC	*C. diphtheriae* (clinical isolate)	Tween 80	0.05% v/v	(−)-(*E*)-Calamenene (14.50%), leptospermone (17.60%)	[137]
		*C. minutissimus* (clinical isolate)				
	MIC	*S. pyogenes* (ATCC 8668)	Acetone	1.00 mg/mL	Eudesma-4(14),11-diene (6.2–14.5%), *α*-selinene (5.90–13.5%), (*E*)-methyl cinnamate (9.2–19.5%)	[138]
		*B. brevis* (ATCC 8246)				
		*B. agri* (ATCC 51663)		0.06 mg/mL		
		*B. laterosporus* (ATCC 64)		0.25 mg/mL		

*Melaleuca alternifolia* (tea tree)	MIC	*Corynebacterium* spp. (10 clinical isolates)	Tween 80	0.06–2% v/v	Terpinen-4-ol (35.70%)	[152]
	MIC_90_	*S. pyogenes* (15 clinical isolates)		0.12%		[235]
	MIC	*C. diphtheriae* (clinical isolate)	Tween 80	0.20% v/v	*α*-Terpinene (11.40%), *γ*-terpinene (22.50%), terpinen-4-ol (35.20%)	[137]
		*C. minutissimus* (clinical isolate)				

*Melaleuca cajuputi* (cajuput)	MIC	*S. pyogenes* (ATCC 12344)	Tween 80	5.00 mg/mL	1,8-Cineol (67.60%)	[128]
	MAC	*C. diphtheriae* (clinical isolate)		0.30% v/v	1,8-Cineole (55.50%)	[137]
		*C. minutissimus* (clinical isolate)				

*Melaleuca quinquenervia* (niaouli)	MAC	*C. diphtheriae* (clinical isolate)	Tween 80	0.25% v/v	1,8-Cineole (61.20%)	[137]
		*C. minutissimus* (clinical isolate)				

*Mentha piperita* (peppermint)	MIC	*S. pyogenes* (ATCC 12344)	Tween 80	5.00 mg/mL	1,8-Cineol (12.06%), menthone (22.24%), menthol (47.29%)	[128]

^a^Scientific name (common name), part of plant (if applicable).

^b^MIC: microdilution method; MAC: macrodilution method; ADM: agar dilution method; CTA: contact time assay.

^c^American Type Culture Collection, Rockville, USA (ATCC).

^d^DMSO concentration was not included; n.m.: not mentioned.

^e^NI: no inhibition.

^f^NCR: no composition results reported.

**Table 7:** Essential oils demonstrating noteworthy antimicrobial efficacy against *C. albicans*.

Essential oil^a^	Method^b^	Species strain^c^	Solvent^d^	Result^e^	Main components^f^	Reference
*Abies balsamea *(fir)	MIC	*C. albicans *(ATCC 10231)	Acetone	2.00 mg/mL	*β*-Pinene (31.00%), bornyl acetate (14.90%), *δ*-3-carene (14.20%)	[99]
*Abies holophylla* (Manchurian fir)		*C. albicans* (B02630)	DMSO	>2.20 mg/mL	Bicyclo[2.2.1]heptan-2-ol (28.05%), *δ*-3-carene (13.85%), *α*-pinene (11.68%), camphene (10.41%)	[111]
*Abies koreana* (Korean fir)					Bornyl ester (41.79%), camphene (15.31%), *α*-pinene (11.19%)	

*Achillea millefolium* subsp. *millefolium* (yarrow)	MIC	*C. albicans* (ATCC 10239)	Tween 80	4.50 mg/mL	Eucalyptol (24.60%), camphor (16.70%), *α*-terpineol (10.20%)	[112]
*Achillea setacea* (bristly yarrow)				1.12 mg/mL	Sabinene (10.80%), eucalyptol (18.50%)	[113]

*Angelica archangelica* (angelica) root	MIC	*C. albicans* (ATCC 10231)	Acetone	2.00 mg/mL	*α*-Phellandrene (18.50%), *α*-pinene (13.70%), *β*-phellandrene (12.60%), *δ*-3-carene (12.10%)	[99]
*Angelica archangelica* (angelica) seed					*β*-Phellandrene (59.20%)	

*Anthemis nobilis* (chamomile)	MIC	*C. albicans* (ATCC 10231)	Acetone	3.00 mg/mL	2-Methylbutyl-2-methyl propanoic acid (31.50%), limonene (18.30%), 3-methylpentyl-2-butenoic acid (16.70%), isobutyl isobutyrate (10.00%)	[99]

*Apium graveolens* (celery)	ADM_90_	*C. albicans* (ATCC 10231)	Tween 80	1.00% v/v	NCR	[236]
*Artemisia dracunculus* (tarragon)	MIC		Acetone	2.00 mg/mL	Estragole (82.60%)	[99]

*Boswellia carteri* (frankincense)	ADM_90_	*C. albicans* (ATCC 10231)	Tween 80	1.00% v/v	NCR	[236]
*Boswellia carteri* (frankincense) (9 samples)	MIC		Acetone	5.30–12.00 mg/mL	*α*-Pinene (4.80–40.40%), myrcene (1.60–52.40%), limonene (1.90–20.40%), *α*-thujene (0.30–52.40%), *p*-cymene (2.70–16.90%), *β*-pinene (0.30–13.10%)	[116]
*Boswellia frereana* (frankincense) (3 samples)				6.00–12.00 mg/mL	*α*-Pinene (2.00–64.70%), *α*-thujene (0.00–33.10%), *p*-cymene (5.40–16.90%)	
*Boswellia neglecta* (frankincense)				1.80 mg/mL	NCR	[117]
				6.60 mg/mL	*α*-Pinene (43.40%), *β*-pinene (13.10%)	[116]
*Boswellia papyrifera* (frankincense)				1.40 mg/mL	NCR	[117]
*Boswellia rivae* (frankincense)				4.00 mg/mL		
*Boswellia sacra* (frankincense) (2 samples)				8.00 mg/mL	*α*-Pinene (18.30–28.00%), *α*-thujene (3.90–11.20%), limonene (11.20–13.10%)	[116]
*Boswellia *spp. (frankincense) (4 samples)				6.00–8.00 mg/mL	*α*-Pinene (18.80–24.20%), limonene (11.70–19.00%)	
*Boswellia thurifera* (frankincense)				6.00 mg/mL	*α*-Pinene (28.00%), limonene (14.60%)	

*Cananga odorata* (ylang-ylang)	ADM_90_	*C. albicans* (ATCC 10231)	Tween 80	1.00% v/v	NCR	[236]
	MIC		Acetone	2.00 mg/mL	Bicyclosesquiphellandrene (19.50%), *β*-farnesene (13.90%)	[99]
	ADM	*C. albicans* (ATCC 10231 and 3 clinical isolates)	Tween 20	0.25–0.50%	NCR	[237]
*Cananga odorata* (ylang-ylang) heads	MIC	*C. albicans* (ATCC 10231)	Acetone	2.00 mg/mL	Benzyl acetate (31.90%), linalool (27.00%), methyl benzoate (10.40%)	[99]

*Canarium luzonicum* (elemi)	MIC	*C. albicans* (ATCC 10231)	Acetone	3.00 mg/mL	Limonene (41.90%), elemol (21.60%), *α*-phellandrene (11.40%)	[99]

*Carum carvi* (caraway)	MIC	*C. albicans* (ATCC 10231)	Acetone	2.00 mg/mL	Limonene (27.60%), carvone (67.50%)	[99]

*Carum carvi* (caraway)	MIC	*C. albicans*	DMSO	≤1.00 *μ*g/mL	DL-Limonene (53.35%), *β*-selinene (11.08%), *β*-elemene (10.09%)	[118]

*Cedrus atlantica* (cedar wood)	ADM_90_	*C. albicans*	Tween 80	>2.00% v/v	NCR	[236]

*Cinnamomum camphora* (camphor)	ADM	*C. albicans* (ATCC 10231 and 3 clinical isolates)	Tween 20	0.50%	NCR	[237]

*Cinnamomum cassia* (cinnamon)	MAC_80_	*C. albicans* (ATCC90029)	n.m.	0.17 *μ*L/mL	*trans*-Cinnamaldehyde (92.20%)	[238]

*Cinnamomum cassia* (cinnamon)	MIC	*C. albicans*	DMSO	≤1.00 *μ*g/mL	*trans*-Caryophyllene (17.18%), eugenol (14.67%), linalool L (14.52%), *trans*-cinnamyl acetate (13.85%), cymol (11.79%), cinnamaldehyde (11.25%)	[118]

*Cinnamomum zeylanicum* (cinnamon)	ADM	*C. albicans*	DMSO	0.08 *μ*L/mL	NCR	[232]
	MIC	*C. albicans* (ATCC 10231)	Acetone	2.00 mg/mL	Eugenol (80.00%)	[99]
	ADM	*C. albicans* (ATCC 10231 and 3 clinical isolates)	Tween 20	0.01%	NCR	[237]

*Citrus aurantium* (petitgrain)	ADM_90_	*C. albicans* (ATCC 10231)	Tween 80	0.25% v/v	NCR	[236]
	MIC		Acetone	2.00 mg/mL	Linalyl acetate (54.90%), linalool (21.10%)	[99]

*Citrus bergamia* (bergamot)	ADM_90_	*C. albicans* (ATCC 10231)	Tween 80	1.00% v/v	NCR	[236]
	ADM	*C. albicans* (ATCC 10231 and 3 clinical isolates)	Tween 20	1.00–2.00%		[237]

*Citrus grandis* (grapefruit)	MIC	*C. albicans* (ATCC 10231)	Acetone	2.00 mg/mL	Limonene (74.80%)	[99]

*Citrus medica limonum* (lemon)	ADM_90_	*C. albicans* (ATCC 10231)	Tween 80	2.00% v/v	NCR	[236]
	ADM	*C. albicans*	DMSO	2.50 *μ*L/mL		[232]
	MIC	*C. albicans* (ATCC 10231)	Acetone	2.00 mg/mL		[99]
	ADM	*C. albicans* (ATCC 10231 and 3 clinical isolates)	Tween 20	0.50–1.00%		[237]

*Citrus limon* (lemon), aromatic art	MAC	*C. albicans* (clinical strain C31)	Tween 80	0.03%	Limonene (22.42%), isopropyl myristate (42.78%)	[239]
*Citrus limon* (lemon), Avicenna				0.60%	Limonene (42.03%), *β*-pinene (15.15%)	
*Citrus limon* (lemon) -Vera Nord					Limonene (23.39%), *trans*-citral (15.52%), *cis*-citral (19.41%)	

*Citrus sinensis* (orange)	MAC	*C. albicans* (ATCC 10231)	0.1% ethanol	3.75 mg/L	NCR	[121]
	MIC		Acetone	2.00 mg/mL	Limonene (93.20%)	[99]
	ADM	*C. albicans* (ATCC 10231 and 3 clinical isolates)	Tween 20	1.00%	NCR	[237]

*Commiphora guidotti* (myrrh)	MIC	*C. albicans* (ATCC 10231)	Acetone	2.00 mg/mL	(*E*)-*β*-Ocimene (52.60%), *α*-santalene (11.10%), (*E*)-bisabolene (16.00%)	[117]
*Commiphora myrrha* (myrrh)				1.50 mg/mL	Furanogermacrene (15.90%), furanoeudesma-1,3-diene (44.30%)	
				4.00 mg/mL	Furanoeudesma-1,3-diene (57.70%), lindestrene (16.30%)	[99]

*Coriandrum sativum* (coriander)	ADM_90_	*C. albicans* (ATCC 10231)	Tween 80	0.25% v/v	NCR	[236]
	MIC	*C. albicans* (CBS 562 and 4 clinical isolates)	n.m.	0.02–0.06 mg/mL	Decanal (10.97%), 1-decanol (15.30%), 2-dodecenol (11.26%)	[240]

*Cupressus sempervirens* (cypress)	MIC	*C. albicans* (ATCC 10231)	Acetone	4.00 mg/mL	*α*-Pinene (41.20%), *δ*-3-carene (23.70%)	[99]

*Cymbopogon citratus* (lemongrass)	ADM_90_	*C. albicans* (ATCC 10231)	Tween 80	0.12% v/v	NCR	[236]
	ADM	*C. albicans* (SP-14)	Sodium taurocholate	5.00 *μ*L/mL	Citral (72.80%)	[125, 126]
	MIC	*C. albicans* (ATCC 10231)	Acetone	2.00 mg/mL	Geranial (44.80%)	[99]
	MIC	*C. albicans*	DMSO	≤1.00 *μ*g/mL	Geranial (47.34%), *β*-myrcene (16.53%), Z-citral (8.36%)	[118]
	ADM	*C. albicans* (ATCC 10231 and 3 clinical isolates)	Tween 20	0.06%	NCR	[237]

*Cymbopogon martinii* (palmarosa)	MIC	*C. albicans* (CBS 562 and 4 clinical isolates)	n.m.	0.06–0.25 mg/mL	NCR	[240]
	ADM	*C. albicans* (SP-14)	Sodium taurocholate	2.00 *μ*L/mL	Geraniol (61.60%)	[125, 126]
		*C. albicans* (ATCC 10231 and 3 clinical isolates)	Tween 20	0.12–0.15%	NCR	[237]
		*C. albicans* (clinical samples)		0.08%		[75]

*Cymbopogon nardus* (citronella)	ADM_90_	*C. albicans* (ATCC10231)	Tween 80	0.25% v/v	NCR	[236]
	MIC		Acetone	0.75 mg/mL	Citronellal (38.30%), geraniol (20.70%), citronellol (18.80%)	[99]
	ADM	*C. albicans* (ATCC 10231 and 3 clinical isolates)	Tween 20	0.50–1.00%	NCR	[237]

*Cymbopogon winterianus* (citronella grass)	MIC	*C. albicans* (CBS 562 and 4 clinical isolates)	n.m.	0.50–1.00 mg/mL	NCR	[240]

*Daucus carota* (carrot seed)	MIC	*C. albicans* (ATCC10231)	Acetone	3.00 mg/mL	Carotol (44.40%)	[99]
	MAC		2% DSMO	1.25–2.50 *μ*L/mL	Sabinene (28.30–33.80%), limonene (6.50–11.80%), elemicin (6.20–26.00%)	[241]
				≥20.00 *μ*L/mL (v/v)	*β*-Bisabolene (17.6%), carotol (25.1), 11*α*H-himachal-4-en-1*β*-ol (21.6%)	[242]
				5.00–10.00 *μ*L/mL (v/v)	*β*-Bisabolene (51.00%), (*E*)-methyl isoeugenol (10.00%)	
					*α*-Pinene (37.9%), geranyl acetate (15%)	
				>20.00 *μ*L/mL (v/v)	Geranyl acetate (65.00%)	

*Eucalyptus camaldulensis* (eucalyptus)	ADM	*C. albicans*	DMSO	5.00 *μ*L/mL	NCR	[232]

*Eucalyptus fruticetorum *(eucalyptus)	ADM_90_	*C. albicans* (ATCC10231)	Tween 80	1.00% v/v	NCR	[236]

*Eucalyptus globulus* (eucalyptus)	ADM	*C. albicans* (clinical samples)	Tween 20	0.05%	NCR	[75]
	MIC_90_	*C. albicans* (ATCC 90028)	Tween 80	10.00 mg/mL	1,8-Cineol (81.93%)	[128]
	ADM	*C. albicans* (SP-14)	Sodium taurocholate	5.00 *μ*L/mL	Cineole (23.20%)	[125, 126]
	MIC	*C. albicans* (ATCC 10231)	Acetone	1.50 mg/mL	1,8-Cineole (58.00%), *α*-terpineol (13.20%)	[99]
	ADM	*C. albicans* (ATCC 10231 and 3 clinical isolates)	Tween 20	1.50%	NCR	[237]
	MIC	*C. albicans* (ATCC 10231)	Acetone	1.00 mg/mL	NCR	[130]

*Eucalyptus radiata* (eucalyptus)	MIC	*C. albicans* (ATCC 10231)	Acetone	1.00 mg/mL	1,8-Cineole (65.7% ± 9.5), *α*-terpineol (12.8% ± 4.4)	[130]
*Eucalyptus camaldulensis* (eucalyptus)				0.50 mg/mL	NCR	
*Eucalyptus citriodora* (eucalyptus)				1.00 mg/mL		
*Eucalyptus smithii* (eucalyptus)						
*Eucalyptus dives* (eucalyptus)						

*Eucalyptus intertexta* (eucalyptus)	MAC	*C. albicans* (ATCC 10231)	10% DSMO	7.80 *μ*g/mL	NCR	[131]
*Eucalyptus largiflorens* (eucalyptus)				125.00 *μ*g/mL	NCR	

*Eugenia caryophyllus* (clove)	ADM	*C. albicans* (clinical samples)	Tween 20	0.33%	NCR	[75]
*Foeniculum vulgare* (fennel)	MIC	*C. albicans*	DMSO	≤1.00 *μ*g/mL	*trans*-Anethole (33.3%), DL-limonene (19.66%), carvone (12.03%)	[118]
*Foeniculum dulce* (fennel)	MIC	*C. albicans* (ATCC 10231)	Acetone	2.00 mg/mL	*E*-Anethole (79.10%)	[99]

*Hyssopus officinalis* (hyssop)	MIC	*C. albicans* (ATCC 10231)	Acetone	1.00 mg/mL	Isopinocamphone (48.70%), pinocamphone (15.50%)	[99]

*Geranium dissectum* (geranium)	MIC	*C. albicans*	DMSO	≤1.00 *μ*g/mL	*β*-Citronellol (25.45%), geraniol (13.83%)	[118]

*Jasminum nudiflorum* (jasmine)	ADM	*C. albicans* (clinical samples)	Tween 20	>3.00%	NCR	[75]
*Juniperi aetheroleum* (juniper)	MAC_80_	*C. albicans* (MFBF)	n.m.	1.00% v/v	*α*-Pinene (29.17%), *β*-pinene (17.84%), sabinene (13.55%)	[135]
*Juniperus chinensis* (Chinese juniper)	ADM	*C. albicans* (clinical samples)	Tween 20	2.00%	NCR	[75]
*Juniperus communis* (juniper berry)	MIC_90_	*C. albicans* (ATCC 90028)	Tween 80	20.00 mg/mL	*α*-Pinene (39.76%)	[128]
*Juniperus communis* (juniper)	ADM_90_	*C. albicans* (ATCC10231)		2.00% v/v	NCR	[236]
*Juniperus communis* ssp. *alpina* (juniper), berries	MAC	*C. albicans* (clinical strain D5)	2% DMSO	1.25–2.50 *μ*L/mL	*α*-Pinene (77.40%)	[243]
*Juniperus communis* subsp. *alpina* (juniper)		*C. albicans* (ATCC 10231)		5.00–10.00 *μ*L/mL		
				2.50 *μ*L/mL	Sabinene (26.20%), *α*-pinene (12.90%), limonene (10.40%)	[244]
*Juniperus virginiana* (juniper)	MIC	*C. albicans* (ATCC 10231)	Acetone	1.50 mg/mL	Thujopsene (29.80%), cedrol (14.90%), *α*-cedrene (12.40%)	[99]
*Juniperus virginiana* (juniper), berries				2.00 mg/mL	*α*-Pinene (20.50%), myrcene (13.70%), bicyclosesquiphellandrene (10.70%)	
*Juniperus virginiana* (juniper)	ADM	*C. albicans* (ATCC 10231 and 3 clinical isolates)	Tween 20	3.00%	NCR	[237]
*Juniperus turbinata* (juniper), berries	MAC	*C. albicans* (ATCC 10231)	2% DMSO	10.00–20.00 *μ*L/mL	*α*-Pinene (66.70%)	[243]
		*C. albicans* (clinical strain D5)		5.00 *μ*L/mL		
*Juniperus turbinata* (juniper), leaf		*C. albicans* (ATCC 10231)	2% DMSO	5.00 *μ*L/mL	*α*-Pinene (48.20%), *β*-phellandrene (23.10%)	
		*C. albicans* (clinical strain D5)		1.25 *μ*L/mL		
*Juniperus oxycedrus* (cade juniper)	ADM	*C. albicans* (clinical samples)	Tween 20	NI	NCR	[75]

*Juniperus oxycedrus* ssp. *oxycedrus* (cade), leaf	MAC	*C. albicans* (ATCC 10231)	2% DMSO	1.25–25.00 *μ*L/mL	*α*-Pinene (65.50%)	[243]
		*C. albicans* (clinical strain D5)		0.32–0.64 *μ*L/mL		
*Juniperus oxycedrus* ssp. *oxycedrus* (cade), berries		*C. albicans* (ATCC 10231)	2% DMSO	10.00–20.00 *μ*L/mL	*α*-Pinene (54.70)%, myrcene (17.80%), germacrene D (10.40%)	
		*C. albicans* (clinical strain D5)		5.00 *μ*L/mL		

*Kunzea ericoides* (Kānuka)	MAC	*C. albicans* (ATCC 10231)	Tween 80	>2.00% v/v	*α*-Pinene (61.60%)	[137]
	MIC	*C. albicans* (ATCC 10231)	Acetone	4.00 mg/mL	*α*-Pinene (26.20–46.70%), *p*-cymene (5.80–19.10%)	[138]
*Laurus nobilis* (bay)				0.75 mg/mL	Eugenol (57.20%), myrcene (14.30%), chavicol (12.70%)	[99]

*Lavandula angustifolia* (lavender)	ADM	*C. albicans* (clinical samples)	Tween 20	>3.00%	NCR	[75]
	MIC	*C. albicans* (ATCC 10231)	Acetone	5.70 mg/mL	Linalool (30.80%), linalyl acetate (31.30%)	[140]
				3.00 mg/mL	Linalyl acetate (36.70%), linalool (31.40%), terpinen-4-ol (14.90%)	[99]
	ADM_90_	*C. albicans* (ATCC 10231)	Tween 80	0.50% v/v	NCR	[236]
				0.25% v/v		

*Lavandula officinalis* (lavender)	MIC	*C. albicans* (ATCC 10231)	DMSO	≤1.00 *μ*g/mL	*δ*-3-Carene (17.14%), *α*-fenchene (16.79%), diethyl phthalate (13.84%)	[118]

*Lavandula pedunculata* (French lavender)	MAC	*C. albicans* (ATCC 10231)	2% DMSO	2.50 *μ*L/mL	1,8-Cineole (2.40–55.50%), fenchone (1.30–59.70%), camphor (3.60–48.00%)	[245]
*Lavandula stoechas* (French lavender)					Fenchone (37.00%) and camphor (27.30%)	[246]
*Lavandula viridis* (yellow lavender)					1,8-Cineole (34.50%–42.2%), camphor (13.40%)	[247]

*Leptospermum scoparium* (manuka)	MAC	*C. albicans* (ATCC 10231)	Tween 80	>2.00% v/v	(−)-(*E*)-Calamenene (14.50%), leptospermone (17.60%)	[137]
	MIC		Acetone	8.00 mg/mL	Eudesma-4(14),11-diene (6.2–14.5%), *α*-selinene (5.90–13.5%), (*E*)-methyl cinnamate (9.2–19.5%)	[138]

*Litsea cubeba* (May Chang)	MIC	*C. albicans* (ATCC 10231)	Acetone	6.00 mg/mL	Geranial (45.60%), nerol (31.20%)	[99]

*Matricaria chamomilla* (German chamomile)	MIC	*C. albicans* (ATCC 10231)	Acetone	0.50 mg/mL	Bisabolene oxide A (46.90%), *β*-farnesene (19.20%)	[99]
	ADM	*C. albicans* (clinical samples)	Tween 20	NI	NCR	[75]

*Melaleuca alternifolia* (tea tree)	ADM	*C. albicans* (clinical samples)	Tween 20	0.73%	NCR	[75]
	MIC	*C. albican*s (KEM H5)	n.m.	0.13% (v/v)	Terpinen-4-ol (39.80%), *γ*-terpinene (17.80%)	[149, 150]
	ADM_90_	*C. albicans* (ATCC10231)	Tween 80	0.25% v/v	NCR	[236]
	ADM			0.20% v/v	Terpinen-4-ol (42.80%), *γ*-terpinene (18.20%)	[115]
	MIC	*C. albicans* (NCYC 854)	n.m.	0.25% v/v	NCR	[145]
		*C. albicans* (fluconazole- and itraconazole-susceptible isolates)	Tween 80	0.06–0.50% v/v	Terpinen-4-ol (42.35%), *γ*-terpinene (20.65%)	[248]
		*C. albicans* (fluconazole- and/or itraconazole-resistant isolates; six isolates were cross-resistant)		0.25–0.50% v/v		
	ADM	*C. albicans* (NRRL y-12983)	n.m.	3.50 mg/mL	NCR	[249]
		*C. albicans* (ATCC 14053)				
		*C. albicans* (NRRL y-869)				
		*C. albicans* (NRRL y-22077)		1.75 mg/mL		
		*C. albicans* (ATCC 10231)		3.50 mg/mL		
	MIC	*C. albicans* (NRRL 12983)		4.73 mg/mL	*γ*-Terpinene (16.30%), terpinen-4-ol (30.30%)	[61]
		*C. albicans* (ATCC 14053)				
		*C. albicans* (ATCC 90028)				
		*C. albicans* (NRRL 22077)		2.30 mg/mL		
		*C. albicans* (ATCC 10231)		4.73 mg/mL		
		*C. albicans* (ATCC 10231)	Acetone	3.70 mg/mL	Terpinen-4-ol (38.60%), *γ*-terpinene (21.60%)	[140]
	MAC	*C. albicans* (ATCC 10231)	Tween 80	0.30% v/v	*α*-Terpinene (11.40%), *γ*-terpinene (22.50%), terpinen-4-ol (35.20%)	[137]
	MIC	*C. albicans* (ATCC 10231)	Acetone	1.50 mg/mL	Terpinen-4-ol (49.30%), *γ*-terpinene (16.90%)	[99]
	ADM	*C. albicans* (ATCC 10231 and 3 clinical isolates)	Tween 20	0.12–0.25%	NCR	[237]

*Melaleuca cajuputi* (cajuput)	MIC_90_	*C. albicans* (ATCC 90028)	Tween 80	2.50 mg/mL	1,8-Cineol (67.60%)	[128]
	MAC	*C. albicans* (ATCC10231)		0.40% v/v	1,8-Cineole (55.50%)	[137]

*Melaleuca quinquenervia* (niaouli)	MAC	*C. albicans* (ATCC10231)	Tween 80	0.40% v/v	1,8-Cineole (61.20%)	[137]

*Melaleuca viridiflora* (niaouli)	MIC	*C. albicans* (ATCC10231)	Acetone	1.75 mg/mL	1,8-Cineole (45.90%), *α*-terpinene (21.00%)	[99]

*Mentha piperita* (peppermint)	ADM_90_	*C. albicans* (ATCC10231)	Tween 80	0.50% v/v	NCR	[236]
	MIC_90_	*C. albicans* (ATCC 90028)		0.30 mg/mL	1,8-Cineol (12.06%), menthone (22.24%), menthol (47.29%)	[128]
	ADM	*C. albicans* (SP-14)	Sodium taurocholate	5.00 *μ*L/mL	Menthol (36.40%)	[125, 126]
	MIC	*C. albicans* (ATCC 10231)	Acetone	2.40 mg/mL	Menthone (18.20%), menthol (42.90%)	[140]
	ADM	*C. albicans* (clinical isolate)	Tween 20	0.08%	NCR	[75]
	MIC	*C. albicans* (clinical isolate)	DMSO	0.31–0.63 mg/mL	Menthol (27.50–42.30%), menthone (18.40–27.90%)	[155]
		*C. albicans*		≤1.00 *μ*g/mL	Menthone (40.82%), carvone (24.16%)	[118]
		*C. albicans* (ATCC 10231)	Acetone	2.00 mg/mL	Menthol (47.50%), menthone (18.60%)	[99]
	ADM	*C. albicans* (ATCC 10231 and 3 clinical isolates)	Tween 20	0.25–0.30%	NCR	[237]
*Mentha pulegium* (peppermint)	MIC	*C. albicans* (ATCC 10231)	10% DMSO	1.00 *μ*L/mL	Piperitone (38.00%), piperitenone (33.00%)	[250]
*Mentha rotundifolia* (peppermint), Beja		*C. albicans*	Tween 80	0.80% v/v	*β*-Caryophyllene (26.67%), germacrene D (12.31%)	[50]
*Mentha rotundifolia* (peppermint), Bizerte					Pulegone (32.09%), piperitenone oxide (17.28%), 5-acetyl thiazole (11.26%)	

*Mentha spicata* (spearmint)	ADM_90_	*C. albicans* (ATCC 10231)	Tween 80	0.12% v/v	NCR	[236]

*Myrtus communis* (myrtle)	MIC	*C. albicans* (ATCC 10231)	Acetone	1.50 mg/mL	Myrtenyl acetate (28.20%), 1,8-cineole (25.60%), *α*-pinene (12.50%)	[99]
*Myrtus nivellei* (Sahara myrtle)	MAC	*C. albicans* (ATCC 10231)	2% DMSO	1.25–2.50 *μ*g/mL	1,8-Cineole (37.50%), limonene (25.00%)	[251]

*Ocimum basilicum *var.* minimum* (basil)	MIC	*C. albicans* (ATCC 11006)	DMSO	NI	Linalool (440%), 1,8-cineole (15.50%)	[252]
*Ocimum americanum* (basil)				5000.00 *μ*g/mL	1,8-Cineole (25.90%), (Z)-methyl cinnamate (29.40%)	
*Ocimum basilicum *var.* purpurascens* (basil)					Linalool (41.50%), *α*-muurolol (11.80%)	
*Ocimum micranthum* (basil)				625.00 *μ*g/mL	Eugenol (64.11%), *β*-caryophyllene (14.30%)	
*Ocimum selloi*				1250.00 *μ*g/mL	Linalool (16.8%), anethole (52.2%)	

*Ocimum basilicum* (basil)	MIC	*C. albicans*	n.m.	30.00 *μ*g/*μ*L	Estragole (45.80%), linalool (24.20%)	[253]
	ADM_90_	*C. albicans* (ATCC10231)	Tween 80	0.50% v/v	NCR	[236]
	ADM	*C. albicans* (clinical samples)	Tween 20	1.50%		[75]
	MIC	*C. albicans* (ATCC 10231)	Acetone	1.00 mg/mL	Linalool (54.10%)	[99]
	MIC_90_		n.m.	95.00 *μ*g/mL	Methyl chavicol (46.90%), geranial (19.10%), neral (15.15%)	[159]
	ADM	*C. albicans* (ATCC 10231 and 3 clinical isolates)	Tween 20	1.00%	NCR	[237]

*Ocimum gratissimum* (African basil)	MIC	*C. albicans* (clinical isolate)	n.m.	750.00 *μ*g/mL	Eugenol (67.00%)	[254]

*Ocimum sanctum* (holy basil)	MIC_90_	*C. albicans* (37 clinical isolates (5 resistant to fluconazole))	10% DMSO	0.10–0.24 *μ*L/mL	Methyl chavicol (44.63%), linalool (21.84%)	[255]
		*C. albicans* (ATCC 90028)		0.25 *μ*L/mL		
		*C. albicans* (ATCC 10261)		0.20 *μ*L/mL		
		*C. albicans* (ATCC 44829)				
	ADM	*C. albicans* (clinical samples)	Tween 20	0.48%	NCR	[75]

*Origanum majorana* (marjoram)	MIC	*C. albicans* (ATCC 10231)	Acetone	2.00 mg/mL	1,8-Cineole (46.00%), linalool (26.10%)	[99]

*Origanum acutidens* (Turkey oregano)	MIC	*C. albicans* (A117)	10% DMSO	125.00 *μ*g/mL	Carvacrol (72.00%)	[161]

*Origanum microphyllum* (oregano)	MIC	*C. albicans*	Tween 80	3.23 mg/mL	Terpinen-4-ol (24.86%), *γ*-terpinene (13.83%), linalool (10.81%)	[162]

*Origanum vulgare* (oregano)	MIC	*C. albicans*	n.m.	2.00 *μ*g/*μ*L	Carvacrol (61.30%), thymol (13.90%)	[253]
	ADM_90_	*C. albicans* (ATCC10231)	Tween 80	0.12% v/v	NCR	[236]
	ADM	*C. albicans* (NRRL y-12983)	n.m.	0.70 mg/mL	NCR	[249]
		*C. albicans* (ATCC 14053)				
		*C. albicans* (NRRL y-869)				
		*C. albicans* (NRRL y-22077)				
		*C. albicans* (ATCC 10231)				
	MIC	*C. albicans* (NRRL 12983)			Cymene (25.00%), cymenol (58.6.00%)	[61]
		*C. albicans* (ATCC 14053)				
		*C. albicans* (ATCC 90028)				
		*C. albicans* (NRRL 22077)				
		*C. albicans* (ATCC 10231)				

*Origanum vulgare* subsp. *hirtum* (Greek oregano)	MAC	*C. albicans* (ATCC 10239)	10% DMSO + Tween 80	85.30 *μ*g/mL	Linalool (96.31%)	[165]
*Origanum vulgare* subsp. *vulgare* (oregano)				128.00 *μ*g/mL	Thymol (58.31%), carvacrol (16.11%), *p*-cymene (13.45%)	

*Pelargonium graveolens* (geranium)	ADM	*C. albicans* (NRRL y-12983)	n.m.	0.70 mg/mL	NCR	[249]
		*C. albicans* (ATCC 14053)				
		*C. albicans* (NRRL y-869)				
		*C. albicans* (NRRL y-22077)				
		*C. albicans* (ATCC 10231)				
	MIC	*C. albicans* (NRRL 12983)	n.m.	0.12 mg/mL	Citronellol (47.30%)	[61]
		*C. albicans* (ATCC 14053)				
		*C. albicans* (ATCC 90028)				
		*C. albicans* (NRRL 22077)				
		*C. albicans* (ATCC 10231)	n.m.	0.06 mg/mL		
		*C. albicans* (ATCC 10231)	Acetone	0.75 mg/mL	Citronellol (34.20%), geraniol (15.70%)	[99]
	ADM	*C. albicans* (ATCC 10231 and 3 clinical isolates)	Tween 20	0.12%	NCR	[237]

*Perovskia abrotanoides* (Russian sage)	MIC	*C. albicans* (ATCC 10231)	10% DMSO	8.00 *μ*L/mL	Camphor (23.00%), 1,8-cineole (22.00%), *α*-pinene 12.00%	[167]

*Pimenta racemosa* (West Indian bay)	ADM_90_	*C. albicans* (ATCC 10231)	Tween 80	0.12% v/v	NCR	[236]

*Pimpinella anisum* (anise)	MAC_83_	*C. albicans*	n.m.	1.00% v/v	Anethole, anisaldehyde, linalool	[256]
	MIC		DMSO	≤1.00 *μ*g/mL	Anethole (64.82%)	[118]

*Pinus sylvestris* (pine)	MIC	*C. albicans* (ATCC 10231)	Acetone	1.50 mg/mL	Bornyl acetate (42.30%), camphene (11.80%), *α*-pinene (11.00%)	[99]

*Piper nigrum* (black pepper)	MIC	*C. albicans* (ATCC 10231)	Acetone	2.00 mg/mL	*β*-Caryophyllene (33.80%), limonene (16.40%)	[99]

*Pogostemon patchouli* (patchouli)	MIC	*C. albicans* (ATCC 10231)	Acetone	1.50 mg/mL	Patchouli alcohol (37.30%), *α*-bulnesene (14.60%), *α*-guaiene (12.50%)	[99]

*Ricinus officinalis* (rose)	ADM	*C. albicans* (clinical samples)	Tween 20	>3.00%	NCR	[75]

*Rosa gallica* (rose)	ADM	*C. albicans* (ATCC 10231 and 3 clinical isolates)	Tween 20	1.00–2.00%	NCR	[237]

*Rosmarinus officinalis* (rosemary)	MIC	*C. albicans* (ATCC 10231)	Tween 80	0.25% v/v	1,8-Cineole (27.23%), *α*-pinene (19.43%), camphor (14.26%), camphene (11.52%)	[169]
				0.10% v/v	1,8-Cineole (26.54%), *α*-pinene (20.14%), camphene (11.38%), camphor (12.88%)	[170]
		*C. albicans* (MTCC 1637)	n.m.	5.50 mg/mL	NCR	[171]
		*C. albicans* (10 antifungal-resistant isolates)		2.75–5.50 mg/mL		
		*C. albicans* (ATCC 10231)	Acetone	5.70 mg/mL	1,8-Cineole (41.40%), *α*-pinene (13.30%), camphor (12.40%)	[140]
				2.00 mg/mL	1,8-Cineole (48.00%)	[99]
	ADM	*C. albicans* (ATCC 10231 and 3 clinical isolates)	Tween 20	1.00%	NCR	[237]

*Salvia eremophila* (sage)	MIC	*C. albicans* (ATCC 10231)	10% DMSO	13.00 *μ*g/mL	Borneol (21.83%), *α*-pinene (18.80%), bornyl acetate (18.68%)	[174]
*Salvia officinalis* (sage)	ADM_90_		Tween 80	0.50% v/v	NCR	[236]

*Salvia ringens* (sage)	MIC	*C. albicans*	n.m.	0.75 mg/mL	*α*-Pinene (12.85%), 1,8-cineole (46.42%)	[177]

*Salvia rosifolia* (sage) (3 samples)	MIC	*C. albicans*	20% DMSO	500.00 *μ*g/mL	*α*-Pinene (15.70–34.80%), 1,8-cineole (16.60–25.10%), *β*-pinene (6.70–13.50%)	[178]

*Salvia sclarea* (clary sage)	MIC	*C. albicans* (13 clinical isolates and 1 reference strain ATCC 10231)	1% DMSO	128.00–256.00 *μ*g/mL	Linalyl acetate (56.88%), linalool (20.75%)	[257]
		*C. albicans* (ATCC 10231)	Acetone	0.88 mg/mL	Linalyl acetate (72.90%), linalool (11.90%)	[99]
	ADM	*C. albicans* (ATCC 10231 and 3 clinical isolates)	Tween 20	2.00%	NCR	[237]

*Santalum album* (sandalwood)	ADM_90_	*C. albicans* (ATCC 10231)	Tween 80	0.06% v/v	NCR	[236]
	MIC		Acetone	2.00 mg/mL	*α*-Santalol (32.10%)	[99]

*Santolina chamaecyparissus* (santolina)	MIC	*C. albicans* (CBS 562 and 4 clinical isolates)	n.m.	0.25–>1.00 mg/mL	NCR	[240]

*Styrax benzoin* (benzoin)	MIC	*C. albicans* (ATCC 10231)	Acetone	2.00 mg/mL	Cinnamyl alcohol (44.80%), benzene propanol (21.70%)	[99]

*Syzygium aromaticum* (clove)	ADM_90_	*C. albicans* (ATCC 10231)	Tween 80	0.12% v/v	NCR	[236]
	MIC	*C. albicans* (ATCC 10231)	Tween 80	0.13% v/v	Eugenol (68.52%), *β*-caryophyllene (19.00%), 2-methoxy-4-[2-propenyl]phenol acetate (10.15%)	[169]
			DMSO	≤1.00 *μ*g/mL	Eugenol (84.07%), isoeugenol (10.39%)	[118]
	MAC	*C. albicans* (ATCC 10231)	DMSO	0.64 *μ*g/mL	Eugenol (85.30%)	[258]
		*C. albicans* (clinical isolate D5)				
		*C. albicans* (clinical isolate D1)				
	MIC	*C. albicans* (ATCC 10231)	Acetone	0.50 mg/mL	Eugenol (82.20%), eugenol acetate (13.20%)	[99]
	ADM	*C. albicans* (ATCC 10231 and 3 clinical isolates)	Tween 20	0.12%	NCR	[237]

*Tagetes minuta* (Mexican marigold)	MIC_90_	*C. albicans* (ATCC 10231)	n.m.	115.00 *μ*g/mL	Dihydrotagetone (33.90%), *E*-ocimene (19.90%), tagetone (16.10%)	[159]
*Tagetes patula* (French marigold)	MIC		Acetone	2.00 mg/mL	(*E*)-*β*-Ocimene (41.30%), *E*-tagetone (11.20%), verbenone (10.90%)	[99]

*Thymus broussonetii* (thyme)	MAC	*C. albicans* (CCMM L4)	n.m.	0.25 mg/mL	Thymol (39.64%), carvacrol (21.31%), borneol (20.13%)	[259]

*Thymus capitatus* (thyme)	MIC	*C. albicans* (ATCC 10231)	Tween 80	450.00 *μ*g/mL	*p*-Cymene (26.40%), thymol (29.30%), carvacrol (10.80%)	[181]
*Thymus capitatus* (thyme), commercial					*α*-Pinene (25.20%), linalool (10.30%), thymol (46.10%)	

*Thymus herba-barona* (thyme)	MAC	*C. albicans* (ATCC 10231)	2% DMSO	0.32 *μ*L/mL	Carvacrol (54.00%), thymol (30.20%)	[246]

*Thymus herba-barona* (thyme), Limbara	MIC	*C. albicans* (ATCC 10231)	Tween 80	450.00 *μ*g/mL	*p*-Cymene (27.60%), thymol (50.30%)	[181]
*Thymus herba-barona* (thyme), Gennargentu				225.00 *μ*g/mL	Thymol (46.90%), carvacrol (20.60%)	
*Thymus magnus* (thyme)		*C. albicans* (KCCM 11282)	Ethanol and Tween 80	0.39 mg/mL	Thymol (39.80%)	[260]

*Thymus maroccanus* (thyme)	MAC	*C. albicans* (CCMM L4)	n.m.	0.25 mg/mL	Carvacrol (89.15%)	[259]
*Thymus mastichina* subsp. *mastichina* (thyme)		*C. albicans* (ATCC 10231, H37, M1)	n.m.	1.25–2.50 *μ*L/mL	1,8-Cineole (67.40%)	[261]

*Thymus quinquecostatus* (thyme)	MIC	*C. albicans* (KCCM 11282)	Ethanol and Tween 80	0.39 mg/mL	Thymol (41.70%), *γ*-terpinene (16.00%)	[260]

*Thymus schimperi* (thyme)	ADM	*C. albicans*	DMSO	0.16 *μ*L/mL	NCR	[232]

*Thymus vulgaris* (thyme)	MIC	*C. albicans*	n.m.	4.00 *μ*g/*μ*L	Thymol (47.90%)	[253]
	MAC	*C. albicans* (ATCC 10231, H37, M1)		0.16–0.32 *μ*L/mL	Carvacrol (70.30%), *p*-cymene (11.70%)	[261]
	MIC	*C. albicans* (ATCC 10231)	Acetone	2.40 mg/mL	Thymol (47.20%), *p-*cymene (22.10%)	[140]
				1.00 mg/mL	*p*-Cymene (39.90%), thymol (20.70%)	[99]

*Thymus* x *viciosoi* (thyme)	MAC	*C. albicans* (clinical isolates M1, D5), *C. albicans* (ATCC 10231)	1% DMSO	0.04–0.64 *μ*L/* *mL	Carvacrol (30.0* *0%), thymol (18.00* *%), *p*-cymene (19.0* *0%)	[262]
*Thymus zygis* subsp. *sylvestris* (thyme)		*C. albicans* (ATCC 10231)	2% DMSO	0.32–1.25 *μ*L/mL	*p-*Cymene (11.00–17.00%), *γ*-terpinene (3.80–11.50%), linalool (3.50–30.00%), geraniol (0.10–19.80%), thymol (5.20–23.80%), carvacrol (1.30–25.00%), geranyl acetate (0.50–20.80%)	[263]
*Thymus zygis* subsp. *zygis* (thyme)		*C. albicans* (ATCC 10231, H37, M1)	n.m.	NI	Thymol (39.60%), *p*-cymene (21.20%)	[261]

*Vetiveria zizanioides/Andropogon muricatus* (vetiver)	ADM	*C. albicans* (clinical samples)	Tween 20	NI	NCR	[75]
	MIC	*C. albicans* (ATCC 10231)	Acetone	1.75 mg/mL	Zizanol (13.60%), *β*-vetirenene (7.20%)	[99]

*Zingiber officinalis* (ginger)	ADM	*C. albicans* (clinical samples)	Tween 20	3.00%	NCR	[75]
		*C. albicans* (ATCC 10231 and 3 clinical isolates)				[237]

^a^Scientific name (common name), part of plant (if applicable).

^b^MIC: microdilution method; MAC: macrodilution method; ADM: agar dilution method, CTA: contact time assay.

^c^American Type Culture Collection, Rockville, USA (ATCC); Colección Espanõla de Cultivos Tipo (CECT); collection of microorganisms of the Department of Microbiology (MFBF); culture collection of antibiotics-resistant microbes (CCRM); Eskişehir Osmangazi University, Faculty of Medicine, clinical isolate (OGU); Laboratorio de Microbiología, Facultad de Ciencias Médicas, Universidad Nacional de Cuyo, Mendoza, Argentina (LM); Microbial Type Culture Collection (MTCC); Mycology Laboratory (LM); National Center of Industrial Microorganisms (NCIM); National Collection of Type Cultures, London, Great Britain (NCTC); Spanish Collection of Type Cultures (STCC).

^d^DMSO concentration was not included; n.m.: not mentioned.

^e^NI: no inhibition.

^f^NCR: no composition results reported.

**Table 8:** Essential oils against dermatophytes and other yeasts.

Essential oil^a^	Method^b^	Species strain^c^	Solvent^d^	Result^e^	Main components^f^	Reference
*Apium nodiflorum* (celery), aerial parts, Italy	MAC	*E. floccosum* (clinical strain FF9)	2% DMSO	0.16 *μ*L/mL	Dillapiole (70.80%), limonene (14.40%)	[230]
		*T. mentagrophytes* (clinical strain FF7)				
		*M. canis* (clinical strain FF1)	2% DMSO	0.04 *μ*L/mL		
		*T. rubrum* (CECT 2794)		0.16 *μ*L/mL		
		*M. gypseum* (CECT 2908)		0.08 *μ*L/mL		
		*T. mentagrophytes *var.* interdigitale* (CECT 2958)		0.16 *μ*L/mL		
		*T. verrucosum* (CECT 2992)		0.32 *μ*L/mL		

*Apium nodiflorum* (celery), aerial parts, Portugal	MAC	*E. floccosum* (clinical strain FF9)	2% DMSO	0.08 *μ*L/mL	Myristicin (29.10%), dillapiole (22.50%), limonene (16.70%)	[230]
		*T. mentagrophytes* (clinical strain FF7)		0.16 *μ*L/mL		
		*M. canis* (clinical strain FF1)		0.04 *μ*L/mL		
		*T. rubrum* (CECT 2794)	2% DMSO	0.08 *μ*L/mL		
		*M. gypseum* (CECT 2908)				
		*T. mentagrophytes *var.* interdigitale* (CECT 2958)	2% DMSO	0.16 *μ*L/mL		
		*T. verrucosum* (CECT 2992)		0.32 *μ*L/mL		

*Cedrus atlantica* (cedar wood)	MIC	*T. erinacei* (KCCM 60411)	Tween 80	2.0 mg/mL	NCR	[264]
		*T. mentagrophytes* (KCCM 11950)		1.00 mg/mL		
		*T. rubrum* (ATCC 6345)				
		*T. schoenleinii* (KCCM 60477)				
		*T. soudanense* (KCCM 60448)		0.25 mg/mL		
		*T. tonsurans* (KCCM 11866)		0.50 mg/mL		

*Cinnamomum zeylanicum* (cinnamon)	ADM	*Trichophyton* spp. (nail isolate)	DMSO	0.31 *μ*L/mL	NCR	[232]
		*Trichophyton* spp. (scalp isolate)		0.16 *μ*L/mL		
		*Microsporum* spp.				

*Citrus bergamia* (bergamot)	MIC	*T. erinacei* (KCCM 60411)	Tween 80	4.00 mg/mL	NCR	[264]
		*T. mentagrophytes* (KCCM 11950)		2.00 mg/mL		
		*T. rubrum* (ATCC 6345)		1.00 mg/mL		
		*T. schoenleinii* (KCCM 60477)		2.00 mg/mL		
		*T. soudanense* (KCCM 60448)		0.50 mg/mL		
		*T. tonsurans* (KCCM 11866)		1.00 mg/mL		
		*T. mentagrophytes* (20 isolates)	Tween 80	0.16–1.30% v/v	NCR	[265]
		*T. rubrum* (18 isolates)		0.16–0.63% v/v		
		*T. interdigitale* (15 isolates)		0.31–1.30% v/v		
		*T. tonsurans* (2 isolates)		2.50% v/v		
		*M. canis* (24 isolates)		0.16–0.63% v/v		
		*M. gypseum* (1 isolate)		2.50% v/v		
		*E. floccosum* (12 isolates)		0.16–0.31% v/v		

*Citrus limon* (lemon)	MIC	*M. canis* (11 clinical isolates)	*Prunus dulcis* (sweet almond oil)	4.60–7.50%	Limonene (59.20%), *β*-pinene (13.70%), *γ*-terpinene (10.80%)	[266]
	ADM	*Trichophyton* spp. (nail isolate)	DMSO	2.50 *μ*L/mL	NCR	[232]
		*Trichophyton* spp. (scalp isolate)				
		*Microsporum* spp.		1.25 *μ*L/mL		

*Cymbopogon citratus* (lemongrass)	MIC	*T. erinacei* (KCCM 60411)	Tween 80	0.25 mg/mL	NCR	[264]
		*T. mentagrophytes* (KCCM 11950)		<0.13 mg/mL		
		*T. rubrum* (ATCC 6345)				
		*T. schoenleinii* (KCCM 60477)				
		*T. soudanense* (KCCM 60448)				
		*T. tonsurans* (KCCM 11866)				
	ADM	*T. mentagrophytes* (SP-12)	Sodium taurocholate	0.25 *μ*g/mL	Citral (72.80%)	[125, 126]

*Cymbopogon martinii* (palmarosa)	ADM	*T. mentagrophytes* (SP-12)	Sodium taurocholate	1.50 *μ*g/mL	Geraniol (61.60%)	[125, 126]

*Cymbopogon winterianus* (citronella)	MIC_90_	*T. rubrum* (15 LM strains), *T. rubrum* strain (ATCC 1683)	Tween 80	312.00 *μ*g/mL	NCR	[84]

*Daucus carota* (carrot seed)	MAC	*E. floccosum* (clinical isolate FF9)	2% DSMO	0.32–0.64 *μ*L/mL	Sabinene (28.30–33.80%), limonene (6.50–11.80%), elemicin (6.20–26.00%)	[241]
		*T. mentagrophytes* (clinical isolate FF7)		0.16–0.64 *μ*L/mL		
		*M. canis* (clinical isolate FF1)	2% DSMO	0.32–0.64 *μ*L/mL		
		*T. rubrum* (CECT 2794)				
		*M. gypseum* (CECT 2905)				
		*E. floccosum* (clinical strain FF9)	DMSO	1.25 *μ*L/mL (v/v)	*α*-Pinene (37.90%), geranyl acetate (15.00%)	[242]
		*T. mentagrophytes* (clinical strain FF7)				
		*M. canis* (clinical strain FF1)				
		*T. rubrum* CECT 2794				
		*M. gypseum* (CECT 2908)				
		*E. floccosum* (clinical strain FF9)	DMSO	0.64 *μ*L/mL (v/v)	Geranyl acetate (65.00%)	
		*T. mentagrophytes* (clinical strain FF7)				
		*M. canis* (clinical strain FF1)				
		*T. rubrum* CECT 2794				
		*M. gypseum* (CECT 2908)				
		*E. floccosum* (clinical strain FF9)	DMSO	0.16–0.32 *μ*L/mL (v/v)	*β*-Bisabolene (17.60%), carotol (25.10%), 11*α*H-himachal-4-en-1*β*-ol (21.60%)	
		*T. mentagrophytes* (clinical strain FF7)	DMSO	0.32 *μ*L/mL (v/v)		
		*M. canis* (clinical strain FF1)				
		*T. rubrum* CECT 2794				
		*M. gypseum* (CECT 2908)				
		*E. floccosum* (clinical strain FF9)	DMSO	0.64 *μ*L/mL (v/v)	*β*-Bisabolene (51.00%), (*E*)-methyl isoeugenol (10.00%)	
		*T. mentagrophytes* (clinical strain FF7)				
		*M. canis* (clinical strain FF1)				
		*T. rubrum* CECT 2794				
		*M. gypseum* (CECT 2908)				

*Eucalyptus camaldulensis* (river red gum eucalyptus)	ADM	*Trichophyton* spp. (nail isolate)	DMSO	5.00 *μ*L/mL	NCR	[232]
		*Trichophyton* spp. (scalp isolate)		2.50 *μ*L/mL		
		*Microsporum* spp.		5.00 *μ*L/mL		

*Eucalyptus globulus* (eucalyptus)	ADM	*T. mentagrophytes* (SP-12)	Sodium taurocholate	0.25 *μ*g/mL	Cineole (23.20%)	[125, 126]
	MIC	*T. erinacei* (KCCM 60411)	Tween 80	0.25 mg/mL	NCR	[264]
		*T. mentagrophytes* (KCCM 11950)				
		*T. rubrum* (ATCC 6345)		<0.13 mg/mL		
		*T. schoenleinii* (KCCM 60477)		0.25 mg/mL		
		*T. soudanense* (KCCM 60448)				
		*T. tonsurans* (KCCM 11866)		<0.13 mg/mL		
	MIC	*M. canis* (ATCC 32903)	Tween 80: DMSO	500.00 *μ*g/mL	1,8-Cineole (72.20%)	[267]
		*M. gypseum* (ATCC 14683)		1000.00 *μ*g/mL		
		*T. mentagrophytes* (ATCC 9533)		250.00 *μ*g/mL		
		*T. mentagrophytes* (ATCC 11480)		<7.80 *μ*g/mL		
		*T. mentagrophytes* (ATCC 11481)		125.00 *μ*g/mL		
		*T. rubrum* (CCT 5507)		62.50 *μ*g/mL		

*Illicium verum* (star anise)	MIC	*M. canis* (11 clinical isolates)	*Prunus dulcis* (sweet almond oil)	1.00–5.00%	*E*-anethole (93.70%)	[266]

*Juniperi aetheroleum* (juniper)	MAC_80_	*M. gypseum* (MFBF)	n.m.	2.00% v/v	*α*-Pinene (29.17%), *β*-pinene (17.84%), sabinene (13.55%)	[135]
		*T. mentagrophytes* (MFBF)		1.00% v/v		
		*T. rubrum* (MFBF)		0.39% v/v		

*Juniperus communis* (juniper)	MIC	*T. erinacei* (KCCM 60411)	Tween 80	0.50 mg/mL	NCR	[264]
		*T. mentagrophytes* (KCCM 11950)		4.00 mg/mL		
		*T. rubrum* (ATCC 6345)		1.00 mg/mL		
		*T. schoenleinii* (KCCM 60477)		2.00 mg/mL		
		*T. soudanense* (KCCM 60448)		0.50 mg/mL		
		*T. tonsurans* (KCCM 11866)		2.00 mg/mL		

*Juniperus communis* ssp. *alpina* (juniper), berries	MAC	*E. floccosum* (clinical strain FF9)	2% DMSO	1.25 *μ*L/mL	*α*-Pinene (77.40%)	[243]
		*T. rubrum* (clinical strain FF5)				
		*T. mentagrophytes* (clinical strain FF7)				
		*M. canis* (clinical strain FF1)				
		*M. gypseum* (clinical strain FF3)				

*Juniperus communis* subsp. *alpina* (juniper)	MAC	*E. floccosum* (clinical strain FF9)	2% DMSO	0.64 *μ*L/mL	Sabinene (26.20%), *α*-pinene (12.90%), limonene (10.40%)	[244]
		*T. rubrum* (clinical strain FF5)		0.32 *μ*L/mL		
		*T. mentagrophytes* (clinical strain FF7)		0.64 *μ*L/mL		
		*M. canis* (clinical strain FF1)		0.32 *μ*L/mL		
		*M. gypseum* (clinical strain FF3)		0.64 *μ*L/mL		
		*T. mentagrophytes *var.* interdigitale* (CECT 2958)		1.25 *μ*L/mL		
		*T. verrucosum* (CECT 2992)		2.5 *μ*L/mL		

*Juniperus turbinata* (juniper), berries	MAC	*E. floccosum* (clinical strain FF9)	2% DMSO	0.64 *μ*L/mL	*α*-Pinene (66.70%)	[243]
		*T. rubrum* (clinical strain FF5)		1.25 *μ*L/mL		
		*T. mentagrophytes* (clinical strain FF7)				
		*M. canis* (clinical strain FF1)		0.32 *μ*L/mL		
		*M. gypseum* (clinical strain FF3)		1.25 *μ*L/mL		
*Juniperus turbinata* (juniper), leaf	MAC	*E. floccosum* (clinical strain FF9)	2% DMSO	0.64 *μ*L/mL	*α*-Pinene (48.20%), *β*-phellandrene (23.10%)	
		*T. rubrum* (clinical strain FF5)		0.64–1.25 *μ*L/mL		
		*T. mentagrophytes* (clinical strain FF7)		1.25 *μ*L/mL		
		*M. canis* (clinical strain FF1)		0.64–1.25 *μ*L/mL		
		*M. gypseum* (clinical strain FF3)		1.25 *μ*L/mL		
*Juniperus oxycedrus* ssp. *oxycedrus* (cade), leaf	MAC	*E. floccosum* (clinical strain FF9)	2% DMSO	0.08–0.16 *μ*L/mL	*α*-Pinene (65.50%)	
		*T. rubrum* (clinical strain FF5)		0.08 *μ*L/mL		
		*T. mentagrophytes* (clinical strain FF7)		0.16 *μ*L/mL		
		*M. canis* (clinical strain FF1)		0.08 *μ*L/mL		
		*M. gypseum* (clinical strain FF3)		0.16 *μ*L/mL		
*Juniperus oxycedrus* ssp. *oxycedrus* (cade), berries	MAC	*E. floccosum* (clinical strain FF9)	2% DMSO	0.32 *μ*L/mL	*α*-Pinene (54.70%), myrcene (17.80%), germacrene D (10.30%)	
		*T. rubrum* (clinical strain FF5)				
		*T. mentagrophytes* (clinical strain FF7)				
		*M. canis* (clinical strain FF1)				
		*M. gypseum* (clinical strain FF3)				

*Kunzea ericoides* (Kānuka)	MAC	*E. floccosum* (clinical isolate)	Tween 80	1.10% v/v	*α*-Pinene (61.60%)	[137]
		*T. rubrum* (clinical isolate)				

*Lavandula angustifolia* (lavender)	MIC	*T. erinacei* (KCCM 60411)	Tween 80	0.50 mg/mL	NCR	[264]
		*T. mentagrophytes* (KCCM 11950)		2.00 mg/mL		
		*T. rubrum* (ATCC 6345)		0.50 mg/mL		
		*T. schoenleinii* (KCCM 60477)		2.00 mg/mL		
		*T. soudanense* (KCCM 60448)		0.25 mg/mL		
		*T. tonsurans* (KCCM 11866)		1.00 mg/mL		

*Lavandula pedunculata* (French lavender)	MAC	*T. mentagrophytes* (clinical strains isolated FF7)	2% DMSO	0.64–1.25 *μ*L/mL	1,8-Cineole (2.40–55.50%), fenchone (1.30–59.70%), camphor (3.60–48.00%)	[245]
		*T. rubrum* (CECT 2794)		0.32–1.25 *μ*L/mL		
		*E. floccosum* (clinical strains isolated FF9)		0.32–0.64 *μ*L/mL		
		*M. canis* (clinical strains isolated FF1)		0.32–1.25 *μ*L/mL		
		*M. gypseum* (CECT 2905)		0.64–2.50 *μ*L/mL		

*Lavandula stoechas* (Spanish lavender)	MAC	*E. floccosum* (clinical isolate FF9)	2% DMSO	0.32 *μ*L/mL	Fenchone (37.00%), camphor (27.30%)	[246]
		*T. mentagrophytes *var.* interdigitale* (CECT 2958)		0.64 *μ*L/mL		
		*T. mentagrophytes* (clinical isolate FF7)				
		*M. canis* (clinical isolate FF1)				
		*T. rubrum* (CECT 2794)				
		*T. verrucosum* (CECT 2992)				
		*M. gypseum* (CECT 2908)				

*Lavandula viridis* (yellow lavender)	MAC	*E. floccosum* (clinical isolate FF9)	2% DMSO	0.32–0.64 *μ*g/mL	1,8-Cineole (34.50% and 42.20%), camphor (13.40%)	[247]
		*T. mentagrophytes *var.* interdigitale* (CECT 2958)		0.32 *μ*g/mL		
		*T. mentagrophytes* (clinical isolate FF7)		0.32–0.64 *μ*g/mL		
		*M. canis* (clinical isolate FF1)		0.32 *μ*g/mL		
		*T. rubrum* (CECT 2794)				
		*T. verrucosum* (CECT 2992)		0.64 *μ*g/mL		
		*M. gypseum* (CECT 2908)		0.32 *μ*g/mL		

*Leptospermum scoparium* (manuka)	MAC	*E. floccosum* (clinical isolate)	Tween 80	0.40% v/v	(−)-(*E*)-Calamenene (14.50%), leptospermone (17.60%)	[137]
		*T. rubrum* (clinical isolate)		0.30% v/v		

*Melaleuca alternifolia* (tea tree)	MIC	*M. furfur* (10 clinical isolates)	Tween 80	0.03–0.12% v/v	NCR	[268]
		*M. sympodialis* (10 clinical isolates)		(0.16 × 10^−1^)–0.12% v/v		
		*E. floccosum*		(0.08 × 10^−1^)–0.03% v/v		
		*M. canis*		(0.04 × 10^−1^)–0.03% v/v		
		*M. gypseum*		(0.16 × 10^−1^)–0.03% v/v		
		*T. interdigitale*		(0.08 × 10^−1^)–0.03% v/v		
		*T. mentagrophytes*		(0.08 × 10^−1^)–0.06% v/v		
		*T. rubrum*		(0.08 × 10^−1^)–0.03% v/v		
		*T. tonsurans*		(0.04 × 10^−1^)–(0.16 × 10^−1^)% v/v		
		*T. erinacei* (KCCM 60411)	tween 80	0.50 mg/mL	NCR	[264]
		*T. mentagrophytes* (KCCM 11950)		1.00 mg/mL		
		*T. rubrum* (ATCC 6345)				
		*T. schoenleinii* (KCCM 60477)		2.00 mg/mL		
		*T. soudanense* (KCCM 60448)		8.00 mg/mL		
		*T. tonsurans* (KCCM 11866)		1.00 mg/mL		
	MAC	*Madurella mycetomatis* (34 clinical isolates)	DMSO	(0.08 × 10^−1^)–0.25% v/v	NCR	[108]
		*E. floccosum* (clinical isolate)	Tween 80	0.70% v/v	*α*-Terpinene (11.40%), *γ*-terpinene (22.50%), terpinen-4-ol (35.20%)	[137]
		*T. rubrum* (clinical isolate)		0.60% v/v		

*Melaleuca cajuputi* (cajuput)	MAC	*E. floccosum* (clinical isolate)	Tween 80	0.60% v/v	1,8-Cineole (55.50%)	[137]
		*T. rubrum* (clinical isolate)				

*Melaleuca quinquenervia* (niaouli)	MAC	*E. floccosum* (clinical isolate)	Tween 80	0.60% v/v	1,8-Cineole (61.20%)	[137]
		*T. rubrum* (clinical isolate)				

*Mentha piperita* (peppermint)	ADM	*T. mentagrophytes* (SP-12)	Sodium taurocholate	3.00 *μ*g/mL	Menthol (36.40%)	[125, 126]
	MIC	Dermatophytes clinical isolates (*M. canis, E. floccosum, T. rubrum, T. mentagrophytes*, *T. tonsurans*)	Tween 80	1.00–2.50 *μ*L/mL	Menthol (37.40%), menthyl acetate (17.40%), menthone (12.70%)	[91]

*Mentha spicata* (spearmint)	MIC	Dermatophytes clinical isolates (*M. canis, E. floccosum, T. rubrum, T. mentagrophytes*, *T. tonsurans*)	Tween 80	0.075–2.25 *μ*L/mL	Carvone (49.50%), menthone (21.90%)	[91]
	MIC_90_	*M. furfur* (clinical isolate)	1% DMSO	125.00 *μ*g/mL	NCR	[269]

*Myrtus nivellei* (Sahara myrtle)	MAC	*E. floccosum* (clinical isolate FF9)	2% DMSO	0.64 *μ*L/mL	1,8-Cineole (37.50%), limonene (25.00%)	[251]
		*T. mentagrophytes* (clinical isolate FF7)		1.25 *μ*L/mL		
		*M. canis* (clinical isolate FF1)		0.64 *μ*L/mL		
		*T. mentagrophytes *var.* interdigitale* (CECT 2958)		1.25 *μ*L/mL		
		*T. rubrum* (CECT 2794)		0.64 *μ*L/mL		
		*T. verrucosum* (CECT 2992)		1.25 *μ*L/mL		
		*M. gypseum* (CECT 2908)				

*Ocimum basilicum* (basil)	ADM	*T. mentagrophytes*	n.m.	8.30 *μ*g/*μ*L	Estragole (45.80%), linalool (24.20%)	[253]
		*T. tonsurans*		8.00 *μ*g/*μ*L		
		*T. rubrum*		8.30 *μ*g/*μ*L		
		*E. floccosum*		15.00 *μ*g/*μ*L		
		*M. canis*		15.20 *μ*g/*μ*L		

*Ocimum gratissimum* (African basil)	ADM	*M. gypseum* (5 clinical isolates)	Tween 80 + DMSO	250.00 *μ*g/mL	NCR	[270]
		*T. rubrum* (10 clinical isolates)		250.00 *μ*g/mL		
		*T. mentagrophytes* (10 clinical isolates)		250.00 *μ*g/mL		
		*M. canis* (5 clinical isolates)		500.00 *μ*g/mL		

*Origanum vulgare* (oregano)	ADM	*T. mentagrophytes*	n.m.	1.00 *μ*g/*μ*L	Carvacrol (61.30%), thymol (13.90%)	[253]
		*T. tonsurans*				
		*T. rubrum*		1.20 *μ*g/*μ*L		
		*E. floccosum*		2.00 *μ*g/*μ*L		
		*M. canis*				
		*M. canis* (11 clinical isolates)	*Prunus dulcis* (sweet almond oil)	0.03–0.05%	*p*-Cymene (14.30%), *γ*-terpinene (11.20%), thymol (45.00%)	[266]

*Pelargonium graveolens* (geranium)	MIC	*T. erinacei* (KCCM 60411)	Tween 80	0.50 mg/mL	NCR	[264]
		*T. mentagrophytes* (KCCM 11950)				
		*T. rubrum* (ATCC 6345)				
		*T. schoenleinii* (KCCM 60477)				
		*T. soudanense* (KCCM 60448)		0.25 mg/mL		
		*T. tonsurans* (KCCM 11866)		0.50 mg/mL		

*Pimpinella anisum* (anise fruits)	MAC_80_	*T. rubrum*	n.m.	0.20% v/v	Anethole, anisaldehyde, linalool	[256]
		*T. mentagrophytes*		0.78% v/v		
		*M. canis*		0.10% v/v		
		*M. gypseum*		0.20% v/v		

*Pogostemon patchouli* (patchouli)	MIC	*T. erinacei* (KCCM 60411)	Tween 80	8.00 mg/mL	NCR	[264]
		*T. mentagrophytes* (KCCM 11950)		>32.00 mg/mL		
		*T. rubrum* (ATCC 6345)		2.00 mg/mL		
		*T. schoenleinii* (KCCM 60477)				
		*T. soudanense* (KCCM 60448)		0.50 mg/mL		
		*T. tonsurans* (KCCM 11866)		8.00 mg/mL		

*Rosmarinus officinalis* (rosemary)	MIC	*T. erinacei* (KCCM 60411)	Tween 80	4.00 mg/mL	NCR	[264]
		*T. mentagrophytes* (KCCM 11950)		8.00 mg/mL		
		*T. rubrum* (ATCC 6345)				
		*T. schoenleinii* (KCCM 60477)		4.00 mg/mL		
		*T. soudanense* (KCCM 60448)		0.50 mg/mL		
		*T. tonsurans* (KCCM 11866)		8.00 mg/mL		
		*T. rubrum*	n.m.	1.38 mg/mL	NCR	[171]
		*M. gypseum*		2.75 mg/mL		
		*M. canis* (11 clinical isolates)	*Prunus dulcis* (sweet almond oil)	2.34–7.50%	1,8-Cineole (27.50%), *α*-pinene (23.40%)	[266]
	MIC_90_	*M. furfur* (clinical isolate)	1% DMSO	260.00 *μ*g/mL	NCR	[269]

*Syzygium aromaticum* (clove)	MAC	*E. floccosum* (clinical isolate FF9)	DMSO	0.16 *μ*g/mL	Eugenol (85.30%)	[258]
		*T. rubrum* (clinical isolate FF5)				
		*T. mentagrophytes*		0.100 *μ*g/mL		
		(clinical isolate FF7)				
		*M. canis* (clinical isolate FF1)		0.08–0.16 *μ*g/mL		
		*M. gypseum*		0.16 *μ*g/mL		
		(clinical isolate FF3)				

*Thymus herba-barona* (thyme)	MAC	*E. floccosum* (clinical isolate FF9)	2% DMSO	0.16 *μ*L/mL	Carvacrol (54.00%), thymol (30.20%)	[246]
		*T. mentagrophytes *var.* interdigitale* (CECT 2958)				
		*T. mentagrophytes* (clinical isolate FF7)				
		*M. canis* (clinical isolate FF1)				
		*T. rubrum* (CECT 2794)				
		*T. verrucosum* (CECT 2992)				
		*M. gypseum* (CECT 2908)				

*Thymus magnus* (thyme)	MIC	*T. rubrum* (ATCC 6345)	Ethanol + Tween 80	0.09 *μ*g/mL	Thymol (39.80%)	[260]

*Thymus quinquecostatus* (thyme)	MIC	*T. rubrum* (ATCC 6345)	Ethanol + Tween 80	0.04 *μ*g/mL	Thymol (41.70%), *γ*-terpinene (16.00%), *p*-cymene (13.00%)	[260]

*Thymus schimperi* (thyme)	ADM	*Trichophyton* spp. (nail isolate)	DMSO	0.31 *μ*L/mL	NCR	[232]
		*Trichophyton* spp. (scalp isolate)		0.08 *μ*L/mL		
		*Microsporum* spp.				

*Thymus serpyllum* (thyme)	MIC	*M. canis* (11 clinical isolates)	*Prunus dulcis* (sweet almond oil)	0.025–0.10%	Carvacrol (72.00%)	[266]

*Thymus tosevii* (thyme)	MIC	Dermatophytes clinical isolates (*M. canis, E. floccosum, T. rubrum, T. mentagrophytes*, *T. tonsurans*)	Tween 80	(0.25–0.75) × 10^−1 ^*μ*L/mL	Geranyl acetate (17.90%), *α*-terpinyl acetate (12.30%), carvacrol (12.80%), thymol (10.40%), *cis*-myrtanol (11.20%)	[91]

*Thymus vulgaris* (thyme)	MIC	*T. erinacei* (KCCM 60411)	Tween 80	0.50 mg/mL	NCR	[264]
		*T. mentagrophytes* (KCCM 11950)		1.00 mg/mL		
		*T. rubrum* (ATCC 6345)				
		*T. schoenleinii* (KCCM 60477)		0.50 mg/mL		
		*T. soudanense* (KCCM 60448)				
		*T. tonsurans* (KCCM 11866)		1.00 mg/mL		
		*T. mentagrophytes*	n.m.	2.20 *μ*g/*μ*L	thymol (47.90%)	[253]
		*T. tonsurans*				
		*T. rubrum*		2.00 *μ*g/*μ*L		
		*E. floccosum*		4.00 *μ*g/*μ*L		
		*M. canis*		2.20 *μ*g/*μ*L		
		Dermatophytes clinical isolates (*M. canis, E. floccosum, T. rubrum, T. mentagrophytes*, *T. tonsurans*)	Tween 80	0.03–0.50 *μ*L/mL	Thymol (48.90%), *p*-cymene (19.00%)	[91]
		*T. rubrum* (clinical isolate)		72.00 *μ*g/mL	Thymol (44.71%), *γ*-terpinene (26.01%), *α*-cymene (21.22%)	[271]

*Thymus zygis* subsp. *sylvestris* (thyme)	MAC	*T. rubrum* (CECT 2794) *T. mentagrophytes* (clinical isolate FF7) *M. canis* (clinical isolate FF1) *M. gypseum* (CECT 2908)	2% DMSO	0.16–0.32 *μ*L/mL	*p*-Cymene (11.00–17.00%), *γ*-terpinene (3.80–11.50%), linalool (3.50–30.0%), geraniol (0.10–19.80%), thymol (5.20–23.80%), carvacrol (1.30–25.00%), geranyl acetate (0.50–20.80%)	[263]

^a^Scientific name (common name), part of plant (if applicable).

^b^MIC: microdilution method; MAC: macrodilution method; ADM: agar dilution method; CTA: contact time assay.

^c^American Type Culture Collection, Rockville, USA (ATCC), Colección Espanõla de Cultivos Tipo (CECT), collection of microorganisms of the Department of Microbiology (MFBF), Korean Culture Center of Microorganisms (KCCM).

^d^DMSO concentration was not included; n.m.: not mentioned.

^e^NI: no inhibition.

^f^NCR: no composition results reported.

**Table 9:** Essential oil with essential oil combinations against skin pathogens.

Essential oil	Species strain	FIC^a^	Result^b^	Reference
*Aniba rosaeodora* (rosewood) + *Thymus vulgaris* (thyme)	*E. coli* (ATCC 25922)	0.23	S	[85]

*Boswellia papyrifera *(frankincense) + *Commiphora myrrha* (myrrh)	*E. coli* (ATCC 8739)	0.65	A	[117]
	*C. albicans* (ATCC 10231)	1.21	I	
	*S. aureus* (ATCC 12600)	0.82	A	
	*P. aeruginosa* (ATCC 27858)	0.77		

*Boswellia neglecta* (frankincense) + *Commiphora guidotti *(myrrh)	*E. coli* (ATCC 8739)	1.46	I	[117]
	*C. albicans* (ATCC 10231)	0.59	A	
	*S. aureus* (ATCC 12600)	2.5	I	
	*P. aeruginosa* (ATCC 27858)	1.04		
	*E. coli* (ATCC 8739)	0.67	A	

*Boswellia neglecta* (frankincense) + *Commiphora myrrha *(myrrh)	*C. albicans* (ATCC 10231)	1.19	I	[117]
	*S. aureus* (ATCC 12600)	3.65		
	*P. aeruginosa* (ATCC 27858)	0.6	A	[117]

*Boswellia papyrifera* (frankincense) + *Commiphora guidotti *(myrrh)	*E. coli* (ATCC 8739)	0.91	A	[117]
	*C. albicans* (ATCC 10231)	1.21	I	
	*S. aureus* (ATCC 12600)	0.5	S	
	*P. aeruginosa* (ATCC 27858)	0.91	A	

*Boswellia rivae* (frankincense) + *Commiphora guidotti* (myrrh)	*E. coli* (ATCC 8739)	1.3	I	[117]
	*C. albicans* (ATCC 10231)	1.38		
	*S. aureus* (ATCC 12600)	2		
	*P. aeruginosa* (ATCC 27858)	1.2		

*Boswellia rivae* (frankincense) + *Commiphora myrrha* (myrrh)	*E. coli* (ATCC 8739)	0.67	A	[117]
	*C. albicans* (ATCC 10231)	2.14	I	
	*S. aureus* (ATCC 12600)	1.27		
	*P. aeruginosa* (ATCC 27858)	0.58	A	

*Cinnamomum zeylanicum* (cinnamon) + *Syzygium aromaticum* (clove)	*S. aureus* (ATCC 29213)	1.8	I	[313]
	*E. coli* (ATCC 29252)	4.2	An	

*Cinnamomum zeylanicum* (cinnamon) + *Thymus vulgaris* (thyme)	*S. aureus* (ATCC 25923)	0.26	S	[85]

*Cymbopogon citratus* (lemongrass) + *Cymbopogon giganteus* (lemongrass)	*E. coli* (CIP 105182)	0.5	S	[124]
	*S. aureus* (ATCC 9144)	0.4		

*Cuminum cyminum* (cumin) + *Coriandrum sativum* (coriander) seed	*S. aureus* (7 clinical isolates)	0.5	S	[122]
	*E. coli* (7 clinical isolates)			

*Juniperus communis* (juniper berry) + *Thymus vulgaris* (thyme)	*S. aureus* (ATCC 25923)	0.74	A	[85]

*Lavandula angustifolia* (lavender) + *Angelica archangelica* (angelica) root	*C. albicans* (ATCC 10231)	0.42	S	[99]
	*S. aureus* (ATCC 6538)	1.07	I	
	*P. aeruginosa* (ATCC 27858)	0.67	A	

*Lavandula angustifolia* (lavender) + *Anthemis nobilis* (chamomile)	*C. albicans* (ATCC 10231)	0.33	S	[99]
	*S. aureus* (ATCC 6538)	0.84	A	
	*P. aeruginosa* (ATCC 27858)	0.54		

*Lavandula angustifolia* (lavender) + *Citrus aurantium* (petitgrain)	*C. albicans* (ATCC 10231)	0.42	S	[99]
	*S. aureus* (ATCC 6538)	1.13	I	
	*P. aeruginosa* (ATCC 27858)	0.51	A	

*Lavandula angustifolia* (lavender) + *Citrus grandis* (grapefruit)	*C. albicans* (ATCC 10231)	0.42	S	[99]
	*S. aureus* (ATCC 6538)	1.67	I	
	*P. aeruginosa* (ATCC 27858)	0.52	A	

*Lavandula angustifolia* (lavender) + *Citrus sinensis* (orange)	*C. albicans* (ATCC 10231)	0.42	S	[99]
	*S. aureus* (ATCC 6538)	0.38		
	*P. aeruginosa* (ATCC 27858)	0.51	A	

*Lavandula angustifolia* (lavender) + *Citrus medica limonum* (lemon)	*C. albicans* (ATCC 10231)	0.42	S	[99]
	*S. aureus* (ATCC 6538)	2.5	I	
	*P. aeruginosa* (ATCC 27858)	0.52	A	

*Lavandula angustifolia* (lavender) + *Abies balsamea* (fir)	*C. albicans* (ATCC 10231)	0.63	A	[99]
	*S. aureus* (ATCC 6538)	2.5	I	
	*P. aeruginosa* (ATCC 27858)	0.52	A	

*Lavandula angustifolia* (lavender) + *Andropogon muricatus* (vetiver)	*C. albicans* (ATCC 10231)	0.45	S	[99]
	*S. aureus* (ATCC 6538)	0.92	A	
	*P. aeruginosa* (ATCC 27858)	1.02	I	

*Lavandula angustifolia* (lavender) + *Angelica archangelica* (angelica) seed	*C. albicans* (ATCC 10231)	0.83	A	[99]
	*S. aureus* (ATCC 6538)	2	I	
	*P. aeruginosa* (ATCC 27858)	0.75	A	

*Lavandula angustifolia* (lavender) + *Artemisia dracunculus* (tarragon)	*C. albicans* (ATCC 10231)	0.42	S	[99]
	*S. aureus* (ATCC 6538)	1.67	I	
	*P. aeruginosa* (ATCC 27858)	0.51	A	

*Lavandula angustifolia* (lavender) + *Cananga odorata* (ylang-ylang)	*C. albicans* (ATCC 10231)	1.25	I	[99]
	*S. aureus* (ATCC 6538)	1.5		
	*P. aeruginosa* (ATCC 27858)	1.02		

*Lavandula angustifolia* (lavender) + *Cananga odorata* heads (ylang-ylang)	*C. albicans* (ATCC 10231)	0.83	A	[99]
	*S. aureus* (ATCC 6538)	1.13	I	
	*P. aeruginosa* (ATCC 27858)	1.02		

*Lavandula angustifolia* (lavender) + *Canarium luzonicum* (elemi)	*C. albicans* (ATCC 10231)	0.25	S	[99]
	*S. aureus* (ATCC 6538)	3.33	I	
	*P. aeruginosa* (ATCC 27858)	0.53	A	

*Lavandula angustifolia* (lavender) + *Carum carvi* (caraway)	*C. albicans* (ATCC 10231)	0.42	S	[99]
	*S. aureus* (ATCC 6538)	1	A	
	*P. aeruginosa* (ATCC 27858)	0.56		

*Lavandula angustifolia* (lavender) + *Cinnamomum zeylanicum* (cinnamon)	*C. albicans* (ATCC 10231)	0.4	S	[99]
	*S. aureus* (ATCC 6538)	0.5		
	*P. aeruginosa* (ATCC 27858)	0.53	A	

*Lavandula angustifolia* (lavender) + *Commiphora myrrha* (myrrh)	*C. albicans* (ATCC 10231)	0.29	S	[99]
	*S. aureus* (ATCC 6538)	1	A	
	*P. aeruginosa* (ATCC 27858)	1.03	I	

*Lavandula angustifolia* (lavender) + *Cupressus sempervirens* (cypress)	*C. albicans* (ATCC 10231)	0.15	S	[99]
	*S. aureus* (ATCC 6538)	0.58	A	
	*P. aeruginosa* (ATCC 27858)	0.53		

*Lavandula angustifolia* (lavender) + *Cymbopogon citratus* (lemongrass)	*C. albicans* (ATCC 10231)	6.67	An	[99]
	*S. aureus* (ATCC 6538)	0.55	A	
	*P. aeruginosa* (ATCC 27858)	0.52		

*Lavandula angustifolia* (lavender) + *Cymbopogon nardus* (citronella)	*C. albicans* (ATCC 10231)	0.42	S	[99]
	*S. aureus* (ATCC 6538)	0.75	A	
	*P. aeruginosa* (ATCC 27858)	0.53		

*Lavandula angustifolia* (lavender) + *Daucus carota* (carrot seed)	*C. albicans* (ATCC 10231)	0.5	S	[99]
	*S. aureus* (ATCC 6538)			
	*P. aeruginosa* (ATCC 27858)	0.56	A	

*Lavandula angustifolia* (lavender) + *Eucalyptus globulus* (eucalyptus)	*C. albicans* (ATCC 10231)	0.38	S	[99]
	*S. aureus* (ATCC 6538)	1.5	I	
	*P. aeruginosa* (ATCC 27858)	0.53	A	

*Lavandula angustifolia* (lavender) + *Foeniculum dulce* (fennel)	*C. albicans* (ATCC 10231)	0.45	S	[99]
	*S. aureus* (ATCC 6538)	2	I	
	*P. aeruginosa* (ATCC 27858)	0.52	A	

*Lavandula angustifolia* (lavender) + *Hyssopus officinalis* (hyssop)	*C. albicans* (ATCC 10231)	0.33	S	[99]
	*S. aureus* (ATCC 6538)	1.67	I	
	*P. aeruginosa* (ATCC 27858)	0.52	A	

*Lavandula angustifolia* (lavender) + *Juniperus virginiana* (juniper)	*C. albicans* (ATCC 10231)	0.5	S	[99]
	*S. aureus* (ATCC 6538)			
	*P. aeruginosa* (ATCC 27858)	0.55	A	

*Lavandula angustifolia* (lavender) + *Juniperus virginiana *berries (juniper)	*C. albicans* (ATCC 10231)	0.21	S	[99]
	*S. aureus* (ATCC 6538)	1.25	I	
	*P. aeruginosa* (ATCC 27858)	0.52	A	

*Lavandula angustifolia* (lavender) + *Laurus nobilis* (bay)	*C. albicans* (ATCC 10231)	0.83	A	[99]
	*S. aureus* (ATCC 6538)	1.7	I	
	*P. aeruginosa* (ATCC 27858)	0.6	A	

*Lavandula angustifolia* (lavender) + *Litsea cubeba* (May Chang)	*C. albicans* (ATCC 10231)	0.19	S	[99]
	*S. aureus* (ATCC 6538)	1.17	I	
	*P. aeruginosa* (ATCC 27858)	0.52	A	

*Lavandula angustifolia* (lavender) + *Matricaria chamomilla* (German chamomile)	*C. albicans* (ATCC 10231)	1.17	I	[99]
	*S. aureus* (ATCC 6538)			
	*P. aeruginosa* (ATCC 27858)	0.54	A	
*Lavandula angustifolia* (lavender) + *Melaleuca alternifolia* (tea tree)	*C. albicans* (ATCC 10231)	0.5	S	[99]
	*S. aureus* (ATCC 6538)	0.63	A	
	*P. aeruginosa* (ATCC 27858)	0.51		

*Lavandula angustifolia* (lavender) + *Melaleuca viridiflora* (niaouli)	*C. albicans* (ATCC 10231)	0.9	A	[99]
	*S. aureus* (ATCC 6538)	2	I	
	*P. aeruginosa* (ATCC 27858)	0.51	A	

*Lavandula angustifolia* (lavender) + *Mentha piperita* (peppermint)	*C. albicans* (ATCC 10231)	0.63	A	[99]
	*S. aureus* (ATCC 6538)	0.75		
	*P. aeruginosa* (ATCC 27858)	0.51		

*Lavandula angustifolia* (lavender) + *Myrtus communis* (myrrh)	*C. albicans* (ATCC 10231)	0.5	S	[99]
	*S. aureus* (ATCC 6538)	4	An	
	*P. aeruginosa* (ATCC 27858)	0.51	A	

*Lavandula angustifolia* (lavender) + *Ocimum basilicum* (basil)	*C. albicans* (ATCC 10231)	0.67	A	[99]
	*S. aureus* (ATCC 6538)	0.58		
	*P. aeruginosa* (ATCC 27858)	0.63		

*Lavandula angustifolia* (lavender) + *Origanum majorana* (marjoram)	*C. albicans* (ATCC 10231)	0.42	S	[99]
	*S. aureus* (ATCC 6538)	4	An	
	*P. aeruginosa* (ATCC 27858)	0.52	A	

*Lavandula angustifolia* (lavender) + *Pelargonium odoratissimum* (geranium)	*C. albicans* (ATCC 10231)	1.04	I	[99]
	*S. aureus* (ATCC 6538)	1.17		
	*P. aeruginosa* (ATCC 27858)	0.52	A	

*Lavandula angustifolia* (lavender) + *Pinus sylvestris* (pine)	*C. albicans* (ATCC 10231)	0.5	S	[99]
	*S. aureus* (ATCC 6538)	0.75	A	
	*P. aeruginosa* (ATCC 27858)	1		

*Lavandula angustifolia* (lavender) + *Piper nigrum* (black pepper)	*C. albicans* (ATCC 10231)	0.42	S	[99]
	*S. aureus* (ATCC 6538)	1	A	
	*P. aeruginosa* (ATCC 27858)	0.57		

*Lavandula angustifolia* (lavender) + *Pogostemon patchouli* (patchouli)	*C. albicans* (ATCC 10231)	0.5	S	[99]
	*S. aureus* (ATCC 6538)	1.17	I	
	*P. aeruginosa* (ATCC 27858)	0.51	A	

*Lavandula angustifolia* (lavender) + *Rosmarinus officinalis* (rosemary)	*C. albicans* (ATCC 10231)	0.42	S	[99]
	*S. aureus* (ATCC 6538)	0.75	A	
	*P. aeruginosa* (ATCC 27858)	0.51		

*Lavandula angustifolia* (lavender) + *Salvia sclarea* (clary sage)	*C. albicans* (ATCC 10231)	0.73	A	[99]
	*S. aureus* (ATCC 6538)	1		
	*P. aeruginosa *(ATCC 27858)	0.51		

*Lavandula angustifolia* (lavender) + *Santalum album *(sandalwood)	*C. albicans* (ATCC 10231)	0.42	S	[99]
	*S. aureus *(ATCC 6538)	2.25	I	
	*P. aeruginosa *(ATCC 27858)	0.51	A	

*Lavandula angustifolia* (lavender) + *Styrax benzoin* (benzoin)	*C. albicans* (ATCC 10231)	0.42	S	[99]
	*S. aureus *(ATCC 6538)	1	A	
	*P. aeruginosa *(ATCC 27858)	0.58		

*Lavandula angustifolia* (lavender) + *Syzygium aromaticum *(clove)	*C. albicans* (ATCC 10231)	0.58	A	[99]
	*S. aureus *(ATCC 6538)	1.17	I	
	*P. aeruginosa *(ATCC 27858)	0.53	A	

*Lavandula angustifolia* (lavender) + *Tagetes patula* (French marigold)	*C. albicans* (ATCC 10231)	0.42	S	[99]
	*S. aureus* (ATCC 6538)	0.75	A	
	*P. aeruginosa* (ATCC 27858)	0.51		

*Lavandula angustifolia* (lavender) + *Thymus vulgaris* (thyme)	*C. albicans* (ATCC 10231)	0.67	A	[99]
	*S. aureus* (ATCC 6538)	0.4	S	
	*P. aeruginosa* (ATCC 27858)	0.51	A	

*Melaleuca alternifolia* (tea tree) + *Backhousia citriodora* (lemon myrtle)	*S. aureus*	n.m.	I	[115]
	*E. coli*			
	*P. aeruginosa*			
	*C. albicans*		A	

*Melissa officinalis* (lemon balm) + *Thymus vulgaris* (thyme)	*E. coli* (ATCC 25922)	0.34	S	[85]

*Mentha piperita* (peppermint) + *Ocimum basilicum* (basil)	*E. coli* (CIP 105182)	0.29	S	[156]
	*S. aureus* (ATCC 9144)	0.36		

*Mentha piperita* (peppermint) + *Thymus vulgaris* (thyme)	*E. coli* (ATCC 25922)	0.55	A	[85]

*Ocimum basilicum* (basil) + *Citrus bergamia *(bergamot)	*S. aureus* (ATCC 6538)	0.38	S	[62]

*Origanum vulgare* (oregano) + *Cinnamomum zeylanicum* (cinnamon)	*E. coli* (ATCC 25922)	n.m.	A	[231]
	*E. coli* (10 clinical isolates, 2 extended-spectrum *β*-lactamase producing)			
	*P. aeruginosa* (ATCC 27853)		I	
	*P. aeruginosa* (clinical isolate)		A	

*Origanum vulgare* (oregano) + *Citrus bergamia* (bergamot)	*S. aureus* (ATCC 6538)	0.38	S	[62]

*Origanum vulgare* (oregano) + *Melissa officinalis* (lemon balm)	*P. aeruginosa* (ATCC 27853)	1.38	I	[314]
	*E. coli* (ATCC 25922)	1.17		

*Origanum vulgare* (oregano) + *Ocimum basilicum* (basil)	*E. coli* (ATCC 8739)	0.75	A	[62]
	*S. aureus* (ATCC 6538)	0.38	S	
	*P. aeruginosa* (ATCC 27853)	1	A	[314]
	*E. coli *(ATCC 25922)			

*Origanum vulgare* (oregano) + *Origanum majorana* (marjoram)	*P. aeruginosa* (ATCC 27853)	1.75	I	[314]
	*E. coli* (ATCC 25922)	0.83	A	

*Origanum vulgare* (oregano) + *Rosmarinus officinalis* (rosemary)	*P. aeruginosa* (ATCC 27853)	1.5	I	[314]
	*E. coli* (ATCC 25922)	1.83		

*Origanum vulgare* (oregano) + *Salvia triloba* (sage)	*P. aeruginosa* (ATCC 27853)	1.5	I	[314]
*Origanum vulgare* (oregano) + *Thymus vulgaris* (thyme)	*P. aeruginosa* (ATCC 27853)	0.88	A	[314]
*Origanum vulgare* (oregano) + *Rosmarinus officinalis* (rosemary)	*S. aureus* (5 clinical isolates)	0.5	S	[315]
*Salvia officinalis* (sage) + *Thymus numidicus* (thyme)	*E. coli* (clinical strain)	1.03	I	[176]

*Syzygium aromaticum* (clove) + *Rosmarinus officinalis* (rosemary)	*S. aureus* (ATCC 6538)	n.m.	A	[169]
	*S. epidermidis* (ATCC 12228)			
	*C. albicans* (ATCC 10231)			
	*P. aeruginosa* (ATCC 27853)		I	
	*E. coli* (ATCC 8739)		A	

*Thymus vulgaris* (thyme) + *Pimpinella anisum* (anise)	*E. coli*	n.m.	A	[168]
	*P. aeruginosa*		S	
	*S. aureus*		A	

^a^n.m.: not mentioned.

^b^S: synergy; A: additive; I: indifference; An: antagonism.

**Table 10:** Essential oil studies demonstrating synergistic interactions in combination with conventional antimicrobials.

Antimicrobial	Essential oil	Microbial species studied	IFIC^a^	Result^b^	Reference
Amoxicillin	*Origanum vulgare* (oregano)	*E. coli*	0.75	A	[324]
	*Elettaria cardamomum* (cardamom)	*S. aureus* (ATCC 25923)	1.06–2.00	I	[325]
		MRSA (clinical isolate)	0.56–1.11	A-I	
			0.61–1.50		

Amphotericin B	*Melaleuca alternifolia* (tea tree)	*C. albicans* (NRRL y-12983, ATCC 14053, NRRL y-869, NRRL y-22077, ATCC 10231)	0.13–1.75	S	[249]
	*Origanum vulgare* (oregano)		0.03–0.35		
	*Pelargonium graveolens* (geranium)		0.04–0.18		
	*Thymus broussonetii* (thyme)	*C. albicans* (CCMM L4)	0.37	S	[259]
	*Thymus maroccanus* (thyme)		0.49		
	*Thymus vulgaris* (thyme)	*C. albicans* (ATCC 90029)	n.m.		[326]

Ampicillin	*Cinnamomum verum* (cinnamon)	*E. coli*	0.75	A	[327]
	*Lavandula angustifolia* (lavender)		2	I	
	*Melaleuca alternifolia* (tea tree)		0.75	A	
	*Mentha piperita* (peppermint)	*E. coli*	1	A	[328]
	*Origanum majorana* (marjoram)		0.63		[327]

Carbenicillin	*Cinnamomum verum* (cinnamon)	*E. coli*	2	I	[327]
	*Lavandula angustifolia *(lavender)				
	*Melaleuca alternifolia* (tea tree)	*E. coli*	0.56	A	[327]
	*Mentha piperita* (peppermint)		0.75		
	*Origanum majorana* (marjoram)	*E. coli*	1.06	I	[327]

Cefazolin	*Cinnamomum verum* (cinnamon)	*E. coli*	0.63	A	[327]
	*Lavandula angustifolia* (lavender)	*E. coli*	2	I	[327]
	*Melaleuca alternifolia* (tea tree)	*E. coli*	1.5		
	*Mentha piperita* (peppermint)	*E. coli*	2	I	[327]
	*Origanum majorana* (marjoram)	*E. coli*			

Cefixime	*Thymus broussonetii* (thyme)	*E. coli*	0.5	S	[329]
		*P. aeruginosa*			
		*S. aureus*			
	*Thymus maroccanus* (thyme)	*E. coli*	0.5	S	[329]
		*P. aeruginosa*	0.75	A	
		*S. aureus*	0.18	S	

Ceftazidime	*Cinnamomum verum* (cinnamon)	*E. coli* (J53 pMG321)	2	I	[327]
	*Lavandula angustifolia* (lavender)	*E. coli* (J53 pMG321)	1	A	[327]
	*Melaleuca alternifolia* (tea tree)				
	*Mentha piperita* (peppermint)	*E. coli* (J53 pMG321)	2	I	[327]
	*Origanum majorana* (marjoram)				

Ceftiofur	*Origanum vulgare* (oregano)	*E. coli*	0.63	A	[324]
Ceftriaxone					

Cefuroxime	*Cinnamomum verum* (cinnamon)	*E. coli*	2	I	[327]
	*Lavandula angustifolia* (lavender)		0.53	A	
	*Melaleuca alternifolia* (tea tree)		1.5	I	
	*Mentha piperita* (peppermint)		0.56	A	
	*Origanum majorana* (marjoram)		0.63		

Chlorhexidine	*Cinnamomum burmannii* (cinnamon)	*S. epidermidis* (clinical isolate)	0.3	S	[211]
		*S. epidermidis* (clinical isolate 64)	0.35		
		*S. epidermidis* (clinical isolate)	0.3		
		*S. epidermidis* strains (ATCC 35984)	0.15		
		*S. epidermidis* (ATCC 12228)	0.45		

Chlorhexidine digluconate	*Eucalyptus globulus* (eucalyptus)	*S. epidermidis* (RP62A)	2	I	[213]
		*S. epidermidis* (clinical isolate)			
	*Melaleuca alternifolia* (tea tree)	*S. epidermidis* (RP62A)			
	*Melaleuca alternifolia* (tea tree)	*S. epidermidis* (clinical isolate TK1)			

Ciprofloxacin	*Melaleuca alternifolia* (tea tree)	*S. aureus*	1.58–7.70	I-An	[304]
	*Mentha piperita* (peppermint)		0.75–1.40	A-I	
	*Pelargonium graveolens* (geranium)	*S. aureus* (ST2)	0.38	S	[330]
	*Rosmarinus officinalis* (rosemary)	*S. aureus*	1.03–1.30	I	[304]
	*Thymus broussonetii* (thyme)	*E. coli*	0.37	S	[329]
		*P. aeruginosa*	0.14		
		*S. aureus*	0.5		
	*Thymus maroccanus* (thyme)	*E. coli*	0.12	S	[329]
		*P. aeruginosa*	0.15		
		*S. aureus*	0.26		
	*Thymus vulgaris* (thyme)	*S. aureus*	0.80–2.59	A-I	[304]
	*Elettaria cardamomum* (cardamom)	*S. aureus* (ATCC 25923)	0.62–1.12	A-I	[325]
		MRSA (clinical isolate)	1.01–2	I	
			1.01–1.50		
	*Salvia officinalis* (sage)	*E. coli* (ATCC 25922)	1.03	I	[176]

Doxycycline	*Origanum vulgare* (oregano)	*E. coli*	0.38	S	[324]
Erythromycin	*Mentha piperita* (peppermint)	*E. coli*	1	A	[328]

Florfenicol	*Origanum vulgare* (oregano)	*E. coli*	0.38	S	[324]

Fluconazole	*Ocimum sanctum* (holy basil)	*C. albicans* (29 clinical isolates)	0.24–0.50	S	[255]
		*C. albicans* (3 clinical isolates)	0.63–0.93	A	
		*C. albicans* (ATCC 90028)	0.48	S	[255]
		*C. albicans* (ATCC 10261)	0.47		
		*C. albicans* (ATCC 44829)	0.48		
	*Thymus broussonetii* (thyme)	*C. albicans* (CCMM L4)	0.3	S	[259]
	*Thymus maroccanus* (thyme)		0.27		
	*Thymus vulgaris* (thyme)	*T. rubrum* (clinical isolate)	0.25		[271]

Gentamicin	*Cinnamomum burmannii* (cinnamon)	*S. epidermidis* (clinical isolate 46)	1.5	I	[211]
		*S. epidermidis* (clinical isolate 64)	0.23	S	
		*S. epidermidis* (clinical isolate 236)	0.15		
		*S. epidermidis* (ATCC 35984)	1.1	I	
		*S. epidermidis* (ATCC 12228)	1.2		
	*Melaleuca alternifolia* (tea tree)	MRSA (2 clinical isolates)	n.m.	I	[144]
	*Mentha piperita* (peppermint)	*E. coli*	1.25		[328]
	*Thymus broussonetii* (thyme)	*E. coli*	0.37	S	[329]
		*P. aeruginosa*	0.28		
		*S. aureus*	0.5		
	*Thymus maroccanus* (thyme)	*E. coli*	0.28	S	[329]
		*P. aeruginosa*	0.18		
		*S. aureus*	0.5		

Kanamycin	*Origanum vulgare* (oregano)	*E. coli*	1.5	I	[324]

Ketoconazole	*Pelargonium graveolens* (geranium)	*T. erinacei* (KCCM 60411)	0.56	A	[264]
		*T. schoenleinii* (KCCM 60477)	0.31	S	[264]
		*T. soudanense* (KCCM 60448)	0.18		
	*Ocimum sanctum* (holy basil)	*C. albicans* (26 clinical isolates)	0.25–0.50	S	[255]
		*C. albicans* (6 clinical isolates)	0.52–0.71	A	
		*C. albicans* (ATCC 90028)	0.42	S	[255]
		*C. albicans* (ATCC 10261)	0.41		
		*C. albicans* (ATCC 44829)	0.5		
	*Thymus magnus* (thyme)	*T. rubrum* (ATCC 6345)	0.37	S	[260]
	*Thymus quinquecostatus* (thyme)		0.35		

Levofloxacin	*Origanum vulgare* (oregano)	*E. coli*	0.5	S	[324]
Lincomycin			0.75	A	

Lysostaphin	*Melaleuca alternifolia* (tea tree)	MRSA (2 clinical isolates)	n.m.	I	[144]
Maquindox	*Origanum vulgare* (oregano)	*E. coli*	0.5	S	[324]

Meropenem	*Cinnamomum verum* (cinnamon)	*E. coli* (clinical isolate)	0.75	A	[327]
	*Lavandula angustifolia* (lavender)		1.5	I	
	*Melaleuca alternifolia* (tea tree)		1	A	
	*Mentha piperita* (peppermint)		0.26	S	
	*Origanum majorana* (marjoram)		1	A	

Mupirocin	*Melaleuca alternifolia* (tea tree)	MRSA (2 clinical isolates)	n.m.	I	[144]

Norfloxacin	*Pelargonium graveolens* (geranium)	*E. coli* (ATCC 35218)	0.57	A	[157]
		*S. aureus* (ATCC 6538)	0.37	S	[157]
		*S. aureus* (ATCC 29213)	0.38		

Nystatin	*Melaleuca alternifolia* (tea tree)	*C. albicans* (ATCC 14053)	>0.5	A	[61]
	*Origanum vulgare* (oregano)	*C. albicans* (ATCC 14053)	0.04	S	[61]
			0.04–0.35		
	*Pelargonium graveolens* (geranium)		0.01–0.06		

Oxytetracycline	*Mentha piperita* (peppermint)	*E. coli*	0.5	S	[328]

Piperacillin	*Cinnamomum verum* (cinnamon)	*E. coli* (J53 R1)	0.5	S	[327]
	*Lavandula angustifolia* (lavender)		0.26		
	*Melaleuca alternifolia* (tea tree)	*E. coli* (J53 R1)	0.56	A	[327]
	*Mentha piperita* (peppermint)		0.31	S	
	*Origanum majorana* (marjoram)		0.75	A	

Polymycin	*Origanum vulgare* (oregano)	*E. coli*	0.75	A	[324]

Pristinamycin	*Thymus broussonetii* (thyme)	*E. coli*	0.37	S	[329]
		*P. aeruginosa*	0.75	A	
		*S. aureus*	0.5	S	[329]
		*E. coli*			
		*P. aeruginosa*	0.75	A	[329]
		*S. aureus*	0.62		

Sarafloxacin	*Origanum vulgare* (oregano)	*E. coli*	0.38	S	[324]

Tobramycin	*Melaleuca alternifolia* (tea tree)	*E. coli* (ATCC 25922)	0.37	S	[97]
		*S. aureus* (ATCC 29213)	0.62	A	

Triclosan	*Cinnamomum burmannii* (cinnamon)	*S. epidermidis* (clinical isolate 46)	1.2	I	[211]
		*S. epidermidis* (clinical isolate 64)	1.5		
		*S. epidermidis* (clinical isolate 236)	1.2		
		*S. epidermidis* (ATCC 35984)	0.03	S	[211]
		*S. epidermidis* (ATCC 12228)	0.06		

Vancomycin	*Melaleuca alternifolia* (tea tree)	MRSA (2 clinical isolates)	>0.50	A	[144]

^a^n.m.: not mentioned.

^b^S: synergy; A: additive; I: indifference; An: antagonism.

**Table 11:** Essential oil studies showing efficacy against viral pathogens associated with skin infections.

Essential oil	Type^a^	CC_50_^b^	IC_50_^c^	SI^d^	[EO]^e^	Reference
*Citrus limon* (lemon)	HSV-1	n.d.	n.m.	n.m.	1.00%	[331]
*Cupressus sempervirens* (cypress)					>1.00%	
*Cupressus sempervirens* ssp. *pyramidalis* (cypress)		>1000.00 *µ*g/mL	>1000.00 *µ*g/mL	>1	n.a.	[332]
*Cymbopogon citratus* (lemongrass)		n.d.	n.m.	n.m.	0.10%	[331]
*Eucalyptus caesia* (eucalyptus)		0.2540%	0.01%	38.81	n.a.	[333]

*Eucalyptus globulus* (eucalyptus)	HSV-1	n.d.	n.m.	n.m.	1.00%	[331]
	HSV-1/HSV-2	0.0300%	0.0090/0.0080%	38.81	n.a.	[334]

*Hyssopus officinalis* (hyssop)	HSV-1/HSV-2	0.0075%	0.001/0.0006%	7.5/13	n.a.	[335, 336]
	HSV-1	0.0075%	0.00%	75		[337]

*Illicium verum* (anise)	HSV-1	160.00 *µ*g/mL	40.00 *µ*g/mL	4	n.a.	[338]
	HSV-2	0.0160%	0.00%	5		[336]

*Juniperus communis* (juniper)	HSV-1	n.d.	n.m.	n.m.	>1.00%	[331]
*Juniperus oxycedrus *ssp. *oxycedrus* (juniper)		1000.00 *µ*g/mL	200.00 *µ*g/mL	5	n.a.	[332]
*Laurus nobilis* (bay)		500.00 *µ*g/mL	60.00 *µ*g/mL	8.3		
*Lavandula latifolia* (lavender)		n.d.	n.m.	n.m.	1.00%	[331]

*Leptospermum scoparium* (manuka)	HSV-1/HSV-2	28.80 *µ*g/mL	0.96 *µ*g/mL/0.58 *µ*g/mL	30/50	n.a.	[339]
*Matricaria recutita* (chamomile)	HSV-1	30.00 *µ*g/mL	0.30 *µ*g/mL	100		[338]
	HSV-2	0.0030%	0.00%	20		[336]

*Melaleuca alternifolia* (tea tree)	HSV-1/HSV-2	0.0250%	>0.0250%	>1	n.a.	[340]
	HSV-1	568.40 *µ*g/mL	13.20 *µ*g/mL	44		[341]
		n.d.	n.m.	n.m.	1.00%	[331]
	HSV-1/HSV-2	0.0060%	0.0009/0.0008%	7/7.5	n.a.	[334]

*Melissa officinalis* (lemon balm)	HSV-1/HSV-2	0.0030%	0.0004%/0.00008%	7.5/37.5	n.a.	[342]

*Mentha piperita* (peppermint)	HSV-1	n.d.	n.m.	n.m.	1.00%	[331]
	HSV-1/HSV-2	0.0140%	0.0020/0.0008%	7/17.5	n.a.	[343]

*Mentha suaveolens* (apple mint)	HSV-1	343.60 *µ*g/mL	5.10 *µ*g/mL	67	n.a.	[341]
*Ocimum basilicum album* (basil)		n.d.	n.m.	n.m.	>1.00%	[331]
*Origanum majorana* (marjoram)					1.00%	

*Pinus mugo* (dwarf pine)	HSV-2	0.0160%	0.00%	5	n.a.	[335]
	HSV-1	40.00 *µ*g/mL	7.00 *µ*g/mL	6		[338]
*Pistacia palaestina* (terebinth)		500.00 *µ*g/mL	500.00 *µ*g/mL	1		[332]

*Rosmarinus officinalis* (rosemary)	HSV-1	0.2580%	0.01%	46.12	n.a.	[333]
		n.d.	n.m.	n.m.	1.00%	[331]

*Salvia officinalis* (sage)	HSV-1	>1000.00 *µ*g/mL	>1000.00 *µ*g/mL	>1	n.a.	[332]
*Santalum album* (sandalwood)	HSV-1/HSV-2	0.0015%	0.0002/0.0005%	7.5/3		[335, 336]
		60.00 *µ*g/mL	2.005 *µ*g/mL/>60.00 *µ*g/mL	2.4/>1		[344]
	HSV-1	0.0015%	0.00%	7		[337]

*Santolina insularis* (santolina)	HSV-1/HSV-2	112.00 *µ*g/mL	0.88/0.70 *µ*g/mL	127/160	n.a.	[345]

*Thymus vulgaris* (thyme)	HSV-1/HSV-2	0.0070%	0.0010/0.0007%	10/14	n.a.	[335, 336]
	HSV-1	0.0070%	0.00%	7		[337]
*Zingiber officinale* (ginger)	HSV-1/HSV-2	0.0040%	0.0002/0.0001%	20/40		[335, 336]
	HSV-1	0.0040%	0.00%	20		[337]

^a^HSV: herpes simplex virus, type 1 or 2.

^b^CC: cytotoxic concentration.

^c^IC: inhibitory concentration.

^d^Selectivity index > 4.

^e^Essential oil concentration at 100% plaque reduction.
